# Supplementary material for: Postpartum blood pressure self-management following hypertensive pregnancy: protocol of the Physician Optimised Post-partum Hypertension Treatment (POP-HT) trial
Source: BMJ Open. 2022 Feb 23;12(2):e051180. doi: 10.1136/bmjopen-2021-051180 (PMC8867381; doi:10.1136/bmjopen-2021-051180)
Supplement: Supplementary data [file bmjopen-2021-051180supp001.pdf]

APPENDIX A: TRIAL FLOW CHART

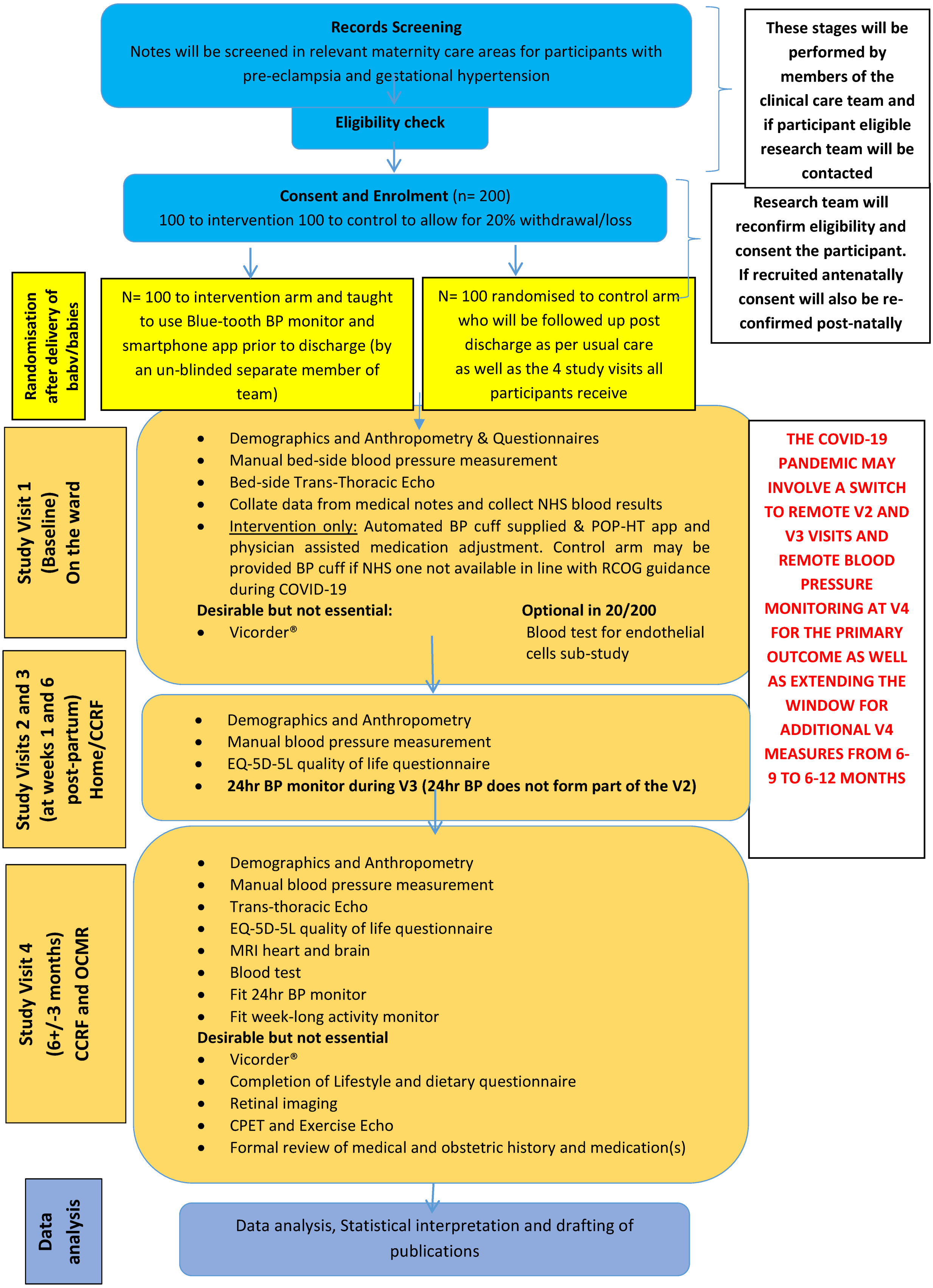

APPENDIX B: SCHEDULE OF PROCEDURES (MAIN STUDY)

**Procedures**	**Visit 0: Consent**		**Visit 1: Baseline**		**Visit 2**		**Visit 3**		**Visit 4**	
	Intervention arm	Control arm	Intervention arm	Control arm	Intervention arm	Control arm	Intervention arm	Control arm	Intervention arm	Control arm
Eligibility assessment	X	X								
Informed consent	X	X								
Clinic/Bed-side BP measurement			X	X	X	X	X	X	X	X
Demographics & anthropometry			X	X	X	X	X	X	X	X
Echocardiogram			X	X					X	X
Data collection: medical notes and NHS blood results			X	X						
Lifestyle & Diet questionnaire			X	X					X	X
Vicorder ®(vascular assessment)			X	X					X	X

Intervention:<br>Automated BP cuff provision and Smartphone app installation			X	During pandemic cuff may be provided						
Home BP self-monitoring and physician assisted medication adjustment post hospital discharge					X		X		X	
EQ-5D-5L questionnaire					X	X	X	X	X	X
**Fitting and performing 24hr ABPM (V3 & V4 only)**							X	X	X	X
MRI of heart and brain									X	X
Blood test			X<br>(if in cells sub-study)	X<br>(if in cells sub-study)					X	X
Fit accelerometer									X	X
Retinal imaging									X	X

Review of medical and obstetric history									X	X
CPET with exercise echo at 40% workload									X	X

APPENDIX C: PROCEDURES FOR ENDOTHELIAL CELLS SUB-STUDY

Procedures	Visit 1: Baseline		Visit 2	
Eligibility assessment	X	X		
Informed consent	X	X		
BP measurement	X	X	X	X
Demographics & anthropometry	X	X	X	X
Blood test	X	X	X	X

APPENDIX D: POP-HT Tele-monitoring system software and network architecture

The software and network architecture of the POP-HT INTERVENTION can be summarised as follows (from left to right in Figure D.1 below): the participants first take BP readings using a Bluetooth-low-energy (BLE) enabled Blood Pressure device (OMRON Evolv®). The data are transmitted to their POP-HT App, available on both Android and iOS mobile devices, via BLE. The App communicates with the POP-HT web-application, available from a public domain on the internet, over encrypted HTTPS, using Representational state transfer (REST) Application Programming Interfaces (APIs), secured by the JSON Web Token protocol. The web-application runs on a web-server hosted in an authorised and secured virtual machine from Oxford University Hospitals (OUH). It includes rule-based algorithms (based on the on/off treatment BP tables below) that process the participants' BP readings and output: (i) the BP level, (ii) the participants' next action, and (iii) the suggested frequency of BP readings. The web-application uses an App notification service (Firebase, Google, USA, https://firebase.google.com) to send messages to the participants' Apps, such as missing data warnings and medication changes. An SMS gateway service (Esendex, UK, https://www.esendex.co.uk) is also used, as a backup, to send messages in case the notification service becomes temporarily unavailable. The SMS system is also responsible for sending and recovering login credentials. E-mails are sent twice daily to clinical researchers, using NHS SMPT servers, summarising the BP and medication status, including triggers to down-titrate, of all study participants in the POP-HT system. The web-application allows participants to review all of their data while e.g., only the last 2 weeks or messages and BP are displayed on the App. Finally, the web-application also allows clinical researchers to register new participants, create and manage their treatment plan, review the participants dashboard, resolve abnormal BP and missing data flags, and export pseudo-anonymised data (identified only by the study IDs) to carry out statistical analysis.

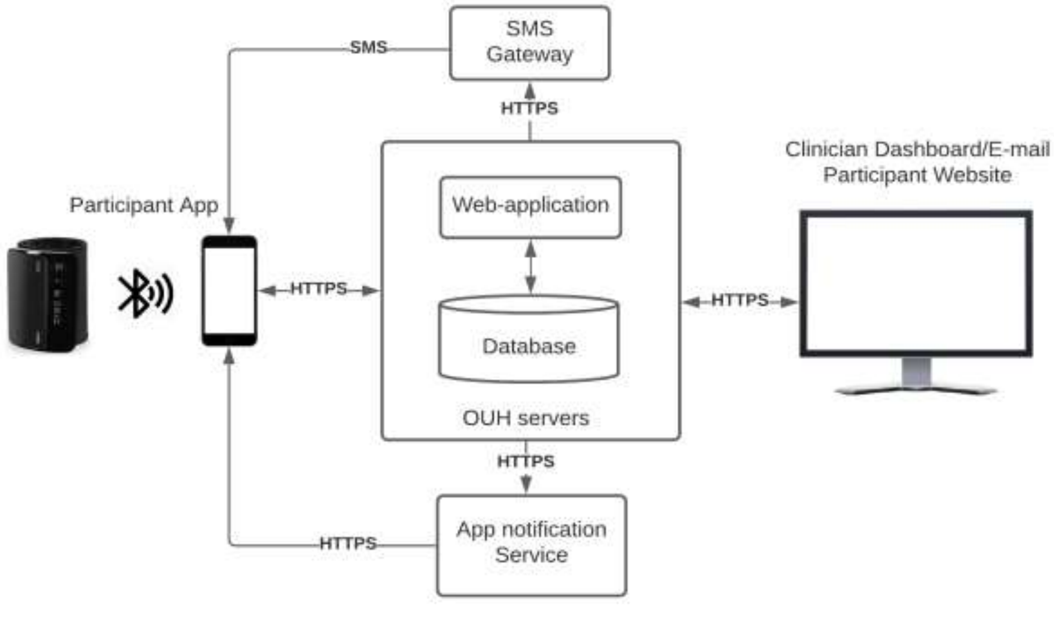

Figure D.1: Illustration of the POP-HT tele-monitoring system software and network architecture

Process of Self-Management

The decisions outlined below in Figure D.2 are based on the participant taking two consecutive BP readings (2 minutes apart), and the automated algorithm decision being made on the second reading. On this note, if more than 2 readings are taken by the participant, all readings are synchronised from the monitor and submitted to the OUH-NHS server, but the decision is made only on the last reading.

Figure D.2 Traffic light table of BP ranges, BP classification and pre-programmed actions whilst ON treatment

Colour	Level	BP	Action
Red	Very high	Sys 160 or more<br>OR<br>Dia 110 or more<br>OR<br>Symptoms	Repeat BP in 5 minutes.<br>If this is a repeat reading: contact local maternity unit immediately for urgent assessment today.
Orange	High	Sys 150-159<br>OR<br>Dia 100-109	Repeat BP in 5 minutes.<br>If this is a repeat reading: Call from study physician 9-5pm AND to see own GP/midwife for an URGENT (same-day) appointment.* Switch to twice daily readings until back in yellow/green
Yellow	Raised	Sys 140-149<br>OR<br>Dia 90-99	No action.
Green	High normal	Sys 130-139<br>OR<br>Dia 80-89	No action
Blue	Low normal	Sys 100-129<br>AND<br>Dia < 80	Switch to twice daily readings and if in this zone for 2 consecutive days, medication titration will be signed off by study doctor and instructions sent via app to participant
Purple	Low	Sys < 100<br>AND<br>Dia < 80	Repeat BP in 5 minutes.<br>If this is a repeat reading: option of opting for a call from study physician 9-5pm vs. opting to see own GP/midwife for an URGENT appointment*. Switch to twice daily readings until back in yellow/green

*During COVID-19, NHS clinicians may not see the patient in person the same day but a tele-medicine review at the minimum will be advised.

If the participant has no 3G/4G signal or Wifi when they synchronise the OMRON readings to the phone, and/or there is an error in the transmission of the readings to the OUH-NHS server, the readings are deemed as 'valid' by the App for 4 hours. After 4 hours, if the participant enters the 'Enter my readings' section and tries to re-sync with the server, they will be asked to take new readings with the OMRON. Once the synchronisation with the server is re-established, all readings, regardless of their timestamp will be sent to the server. The SMS system is also a backup to allow the participants to notify the study doctors of their readings. The messages used are essentially the same, small changes only being required to map the same App functionality to the more "limited" SMS system.

The frequency of readings requested of the participant via the app will be adjusted according to whether the readings are high, low normal or low. If they are in the orange, blue or purple

zones above (high, low normal or low), they will be asked by the app to submit readings twice daily.

If the reading is abnormal, as outlined in the table they will be asked to 'Repeat the BP in 5 minutes'.

If the repeat reading submitted is in the red zone i.e.>160/110mmHg they will be sent a notification asking them to contact their local maternity unit immediately for urgent assessment (see 'Safety Netting' section below for more detail).

If the repeat reading is in the orange or purple BP ranges above, participants will be notified via an app notification and will have the option of:

- Selecting via the app to be called by a 'physician' (who will be a clinician within the research team) to discuss and make medication adjustment over the telephone,
- And seeing their GP/Midwife/maternity unit on an urgent basis to have their medications adjusted, which they will then update on the app.

Medication titration will be done in line with the updated NICE guidance NG133 in the intervention arm. During COVID-19 given the difficulties in seeing their own GP/mid-wives, it is expected more women will want remote physician support. The software will send a notification to the participant using more 'participant friendly' wording as agreed in several rounds of PPI and as tested on real-life volunteers as part of the PPI process: For example, if the blood pressure reading submitted is 152/100mmHg it will trigger this notification:

'*Your blood pressure reading is a little higher than we would ideally like it and the repeat reading was also a little high. Select* ***Yes*** *to be called by a specialist doctor to have your medication adjusted or* ***No*** *to see your own GP/midwife/maternity unit urgently?*

They then have the option of selecting '**YES'** or '**No'** on the app which triggers the following actions to be taken

- **Yes**: The study team will call them the same working day (if out of hours, at the beginning of the next working day) to review their symptoms and to adjust their medications. If the doctor calling has any concern or if the participant feels unwell prior to the call then they will be aware of the need to see a health professional from the information sheet, the app and/or the call, as appropriate. During the call, they will be advised of the need to have blood tests checked if this reading is in the 1st 2 weeks' post-partum in the orange zone. This should be done over the next 24 hours by the GP/local maternity unit. They will also be asked to monitor twice daily until 2 sets of readings are back in the normal (yellow/green zones). The medication schedule will be updated on the web-platform after the call and synchronise to the app so it will be written down for the participant too. The next day the participant will be asked to confirm their new medication schedule on the app with a YES/NO as a safety check. If it is NO they will be asked to enter, what they are taking and any discrepancy will be clarified with the research team over the phone.
- **No**: The app will notify them that urgent midwife/GP assessment is advised and the team will call the same day to check this is in progress and enquire about any medication adjustment. They will also be asked to monitor twice daily until 2 sets of readings are back in the normal (yellow/green zones).

'Safety netting' built into the system

The app has been tested for in detailed simulation scenarios e.g. high BP, low BP, fluctuant BP, non-compliers, anxious and for all eventualities based on our experience from SNAP-HT [7] and it has proved robust in these 'test scenarios'. Automatic e-mail alerts will be triggered

to the clinical members of the study team and flags will generate on the website next to that participant's study ID, for those BP readings 'out of target range', and/or if readings are consistently not being recorded and/or uploaded via the app by a particular participant. These secure e-mails will be sent (as explained further in appendix E) so that appropriate action can be taken in a timely fashion to adjust medications for those readings out of range as explained above. For those readings in the red zone, a phone call will also be made that same working day (9-5pm) to ensure arrangements have been made for assessment by an NHS provider, and in the event they cannot be contacted, their GP/midwife will be notified.

Once participants have switched to once weekly readings i.e. they have been off-treatment for 5 days with readings in the normal range, and have been notified by the app to make this switch, motivational reminders will be sent on a weekly basis. If the readings go up or down outside of the normal range whilst doing once weekly readings i.e. if they are not in the green or blue zones, they will be asked to repeat the reading and if the repeat reading is also outside of these zones, the relevant actions will be triggered as outlined below in figure D.3

Figure D.3 Traffic light table of BP ranges classification and pre-programmed actions whilst OFF treatment

Colour	Level	BP	Action
Red	Very high	Sys 160 or more<br>OR<br>Dia 110 or more	Repeat BP in 5 minutes.<br>If this is a repeat reading*: contact your local maternity unit immediately for urgent assessment today. Switch to once daily readings
Orange	High	Sys 140-159<br>OR<br>Dia 90-109	Repeat BP in 5 minutes.<br>If this is a repeat reading and the value is in this range for 2 or more days in a row option of opting for a call from study doctors between 9-5pm vs. opting to see own GP/midwife in next 48hrs. Switch to once daily readings until further notification
Green	Normal	Sys < 140<br>OR<br>Dia < 90	No action. Continue weekly readings
Blue	Low normal	Sys 100-129<br>AND<br>Dia < 80	No action unless symptoms (report via the app/website) e.g. light-headed/dizzy/faint in which case please notify study team who will call you within 48 hours
Purple	Low	Sys < 100<br>AND<br>Dia < 80	Repeat BP in 5 minutes.<br>If this is a repeat reading and the value is in this range for 2 or more days in a row option of opting for a call from study doctors between 9-5pm vs. opting to see own GP/midwife in next 48hrs. Switch to once daily readings until further notification

*If the repeat reading is 'Red' a flag/notification will also be sent to the study team to call the participant to check they have followed the action just as for the on-treatment algorithm. For orange and purple zones, the research team will also be notified and the participants will be asked to switch back to daily readings. If the readings remain outside of the green/blue zones for >2 days the app will offer the option of being contacted shortly by the study team or opting

to see their own GP/midwife, as per the pathways for the on-treatment algorithm. It is unlikely that women will need to-restart treatment based on the pilot data from SNAP-HT [4], where only 1/91 women needed to re-start medication after stopping.

In the case of failure to submit readings, automatic 'motivating' notifications will be sent at 24hours to the participants, and notifications are also sent to the study team after 36 hours without a reading during the first 2 weeks following discharge. This is to prompt a call to the participant to discuss any problems. If the participant repeatedly fails to submit a reading for >36 hours during the first 2 weeks, then they may be withdrawn from the study at the discretion of the PI/CI. Their GP will also be called and mailed; and the participant will be called, messaged and e-mailed advising urgent medical review.
Similar 'safety alerts' will be sent out if a participant records any side-effects/SAEs (which can be accessed and reviewed by the study team via a secure log-on portal). If there is anything deemed to require further action the PI/CI, GP and participant will be notified urgently.

APPENDIX E: MODIFICATIONS TO STUDY DESIGN TO MITIGATE THE IMPACT OF COVID-19

On March 17th 2020, the COVID-19 pandemic meant that all research across Oxford University Hospitals (OUH) NHS Foundation Trust had to be halted. A minor amendment to perform remote V2 and V3 visits allowed us to continue the follow up of 18/200 women already recruited as of 06/05/2020. A further two amendments allowed the POP-HT RCT to restart safely during the COVID-19 pandemic as the study received a Stage 3 exception from OUH NHS Foundation Trust, due to its contribution to the clinical care of these women. Recruitment to the trial recommenced in early June 2020.
The following changes are explained in more detail below to explain how the study will run safely and in line with local COVID-19 guidance:
Remote study visits were established early to allow entirely remote follow up visits for week 1 and week 6, equivalent to visits 2 and 3 (non-substantial amendment 2.0 25/03/20201). The 1st 18 participants' recruited pre-COVID had been followed up remotely successfully, demonstrating the technique to be both effective and feasible for the remaining 182/200. None of the 1st 18 participants required face-face contact for review of their medications and obstetric history, or for use the POP-HT app and the solving of any technical issues for those in the intervention arm. Remote blood pressure measurement (both clinic and ambulatory 24hr blood pressure monitoring) was achieved successfully for all of the 1st 18 participants recruited pre-COVID 19 using ZOOM® or MS TEAMS®. During this period of follow up, no new baseline visits were performed during the 1st wave of the COVID-19 pandemic.
When the study restarted recruitment in June 2020 the following amendments were made to the baseline visit with re-design of the recruitment, consent and enrolment process to minimise direct patient contact and risk of virus transmission:

a. Provision of documents: PIS, flyer and additional information sheet provided by the clinical team to the participant on a Tablet/Ipad® (sterilised with CLINELL® wipes). The participants can then review them in this format (and a copy e-mailed to them for their records once consent has been obtained and they are enrolled).
b. Consent: Consent forms will be placed in wipe down wallets, which will be handed to the participant for signing wearing sterile gloves. Once signed the form will be photocopied whilst wearing gloves. The copy will then be placed

back into a sterile wallet for the participant and the original will be placed in a second wallet in their notes. Both will be wiped down with CLINELL® wipes and the research teams' copy will be kept securely in a wipe-down file/ring-binder in 'quarantine' before moving them to CCRF after 48hours.

c. The Vicorder® was made an optional measurement to reduce the amount of time in direct patient contact
d. The Echo and Vicorder® will be performed by a single investigator at the bedside. PPE will be worn (the level of which will be in line with hospital policy). Adequate training in donning and doffing of PPE has been undertaken by the study team via OXSTAR®. This will not affect OUH trust protocols for female chaperones, which can still be provided if needed by existing clinical staff on the ward.
e. **In line with RCOG guidance, produced during COVID-19 in June 2021, the control arm will also provided with a home monitor on discharge for the first 'few weeks'**
f. The V2 and V3, at weeks 1 and 6 respectively, will be continued remotely for participant 19 onwards once recruitment restarted.
g. V4 can also be done remotely for the primary outcome measures if a national lockdowns necessitate such a step to be taken.

APPENDIX F: AMENDMENT HISTORY

All amendments have been submitted to; and approved by SPONSOR, the REC and HRA, the local hospital (OUH) trial management authority; and other relevant parties are notified where needed.

Amendment No.	Protocol Version No.	Date issued	Author(s) of changes	Details of Changes made
01 (minor)	N/A	21/01/2020	J Kitt	Correction to wording of consent form clauses<br><br>Correction of version listed in flyer footer<br><br>IRAS form updated to list PI for OUH site
02 (minor)	V2.0	19/03/2020	P Kemp	Add option for Visit 2 (Weeks 1) and Visit 3 (Week 6) to be conducted remotely by video call.<br><br>Add the option of sending a sterile OMRON EVOLV BP monitor to participants prior to Visit 3 (Week 6).<br><br>Make the "fitting of a home blood pressure monitor" procedure optional for Visit 3 and add the option

				of conducting this at a later time point.
03 (minor)	V3.0	22/04/2020	J Kitt and P Kemp	Changes to visits during the COVID-19 pandemic:<br>Add provision of BP monitor to control arm;<br><br>Consent process to be modified to reduce risk of transmissions of COVID-19;<br><br>Extend the time point for Visit 4 to 6-12 months from 6-9;<br><br>Change the randomisation and blinding process so that only one member of staff (wearing PPE where necessary) is present at Visit 1;<br><br>Minimise participant contact during the baseline visit via use of tablets/iPads for reviewing the PIS/flyer;<br><br>Vicorder is now an optional measurement during the baseline visit.
04 (minor)	V4.0	06/10/2020	A Frost and P Kemp	Add blood test to baseline visit in both control and intervention hypertensive groups.<br><br>Include a sub population of 20 normotensive postnatal women for a new blood validation sub study.
05 (minor)	V5.0	05/01/2021	J Kitt and P Kemp	Add option for Visit 4/Final visit to be performed remotely by video call for the primary study outcome and BP based secondary outcome measures
06 (major)	V6.0	08/03/2021	J Kitt and P Kemp	Addition of an extra sequence during the MRI scan at 6-12months to allow the kidneys to be evaluated at the same time as the heart and brain sequences are being performed. Scan duration will still remain under 60 minutes

APPENDIX G: COPIES OF PARTICIPANT INFORMATION SHEET (PIS) AND INFORMED CONSENT FORMS (ICF)

Division of Cardiovascular Medicine
Radcliffe Department of Medicine &
Nuffield Department of Primary Care Health Sciences,
Oxford Heart Centre, John Radcliffe Hospital,
Oxford, OX3 9DU Tel: 01865 572833
Email: jamie.kitt@cardiov.ox.ac.uk

Physician Optimised Post-partum Hypertensive Treatment (POP-HT) Study

Participant Information Sheet (PIS): Information about a study you are invited to join

We would like to invite you to take part in a research study. Joining is entirely up to you. This information sheet explains why the research is being conducted and what it would involve if you did decide to take part. If you have any questions, please do not hesitate to ask. Please feel free to talk to others about the study.

Summary of the study

- **High blood pressure occurs in ~1 in 10 women during pregnancy** and **remains elevated in 50% even after the baby is born requiring medication at home after discharge.**
- This **study** is **investigating** whether, **'self- management' of blood pressure (BP) at home**, **after discharge**, can improve your blood pressure control and reduce the longer-term impact on your heart and brain.
- **50%** of participants (**the self-management group**) will be asked to **measure their blood pressure at home** using a **Wireless monitor** and **upload the readings via** a **smart-phone/tablet app.** If readings are abnormal then a specialist doctor can guide your medication adjustment accordingly (you will still have option of seeing your own GP/midwife if you prefer).
- **50%** (**the control group**) **will be looked after by their GP/ midwife/other NHS services as normal**. The group you are in will be decided by random. During the COVID-19 pandemic a BP monitor will also be provided to the control arm to allow home readings to be measured as per updated national guidelines. See page 11 for other amendments during the COVID-19 pandemic.
- The **study starts after giving birth** and **runs up-to 12 months** after discharge from hospital and **both the intervention and control group will have 4 study visits** as part of the trial.
- The team are aware that you will have a new born baby/babies so **study visits will be very flexible** and can stop-start around feeding, nappy changes and other important baby needs.

N.B. All women will continue to receive routine NHS care throughout.

Chief Investigator:	Prof Paul Leeson	Version/date:	6.0 23.03.2021
Study Short Title:	POP-HT STUDY	Ethics Ref:	19/LO/1901
		IRAS ID:	273353
Document:	Participant Information Sheet		

Why we are doing this study?

High blood pressure disorders in pregnancy are associated with an increased risk of high blood pressure, heart attack and stroke in later life. The risks associated with high blood pressure in pregnancy can be mitigated by early recognition and treatment of raised blood pressure (and other traditional risk factors e.g. lack of regular exercise, an unhealthy diet. This trial is looking at the impact that blood pressure control has on these long-term risks. We plan to assess whether blood pressure self-management can improve blood pressure control and whether this reduction in blood pressure in the months after birth can reduce the long-term effects these conditions have on your heart, brain and blood vessels, in turn reducing the risk of the events listed below, which include:

1 in 5 women having another hypertensive pregnancy

1 in 7 women having another pre-eclamptic pregnancy

A 4x increase in risk of having long term high blood pressure

A 2x increase in risk of experiencing both cardiovascular death or a heart attack

A 1.5x increase in risk of having a stroke

(Figure adapted from NICE NG 133: Hypertension in pregnancy July 2019)

Why have I been invited?

If you have pre-eclampsia or gestational hypertension and still require medication after delivering your baby/babies at the time of discharge.

Do I have to take part?

No, this is a voluntary study, and it is your decision to participate. You are free to withdraw from the study at any time without giving a reason. This would not affect the standard of care you receive. If you decide that you no longer wish to continue with the study, we would still retain any data already obtained from you unless you request otherwise.

What will happen if you take part in the POP-HT study:

Both groups receive the same number of study visits i.e. 4 visits for all participants.

Visit 1 (90 minutes) will take place in the first days after giving birth whilst you remain on the post-natal ward in the Women's centre. We will **measure your blood pressure**, **scan your heart (using an ultra-sound scan like you had of the baby), take a blood test and review your medical notes and blood tests** and e-mail a **questionnaire about your lifestyle and diet,** which can be completed later to

Chief Investigator:	Prof Paul Leeson	Version/date:	6.0 23.03.2021
Study Short Title:	POP-HT STUDY	Ethics Ref:	19/LO/1901
		IRAS ID:	273353
Document:	Participant Information Sheet		

shorten the visit**. At the end of visit 1** you will either be **allocated at random** to the **intervention group** or **the control group.** If allocated to the **intervention** group a separate member of the team will come and provide you with the blood pressure monitor and install the app on your smartphone/tablet and teach you how to use it. You will have plenty of time to practice whilst still in hospital!

Visits 2 and 3 (30 minutes) take place at **weeks 1 and 6 weeks after discharge respectively** and are to measure your blood pressure, take some simple measurements e.g. waist, left arm and hip circumference, and to complete a brief questionnaire. These can be done as a **home visit**, or as a visit to us in the Cardiovascular Clinical Research Facility (CCRF) at the **John Radcliffe Hospital**. At the end of **Visit 3** (week 6) there will be a 24 hour blood pressure monitor fitted, programmed to be silent to minimise disruption to you and your baby, and we will provide you with a stamped, addressed envelope to post it back to us at CCRF.

At 6-12 months there will be a **slightly longer visit to our research facility** (CCRF) in the **John Radcliffe Hospital. Visit 4** (up to 4 hours) will involve measuring your blood pressure again, doing another scan of your heart by ultra-sound, doing an MRI (magnet scan) of your heart and brain, and taking a blood test. There will also be a brief review of your medical and obstetric history and medications, and a few other tests (not absolutely mandatory), which include: taking photos of the blood vessels in the back of your eyes, and doing some gentle exercise on a bike (akin to walking up a hill at a fast pace) during which we measure your heart rate, blood pressure and, scan your heart briefly with the ultra-sound machine. The study finishes with another period of 24hr blood pressure monitoring and a wrist-watch (accelerometer) you wear for 1 week. ***The additional PIS you have also been given provides more detail specifically about this 4th visit. We will run through this again at the end of your 3rd visit at 6 weeks so you can have a chance to discuss any questions and we can help plan child care for the 4th and final longer visit at the John Radcliffe Hospital.***

We would like to follow you up for up to 10 years. For this longer term follow up, the research team in Oxford will ask for information about your health from NHS Digital (we will send your name, date of birth, NHS number and postcode to NHS Digital and ONS (or other central NHS bodies) who can link this information to your centrally held records to allow your blood pressure records to be reviewed if needed. We will access these records so that we can assess long term health outcomes and in particular monitor your long term blood pressure control in line with one of the study objectives.

We may also want to measure your blood pressure again in the future (up to 10 years from enrolment) as a home visit/visit to the hospital. This will be an extension to this study and we would like to contact you again to ask you to consider further participation.

Further details of the individual study procedures over the 6-month period are as follows:

Kitt J, *et al. BMJ Open* 2022; 12:e051180. doi: 10.1136/bmjopen-2021-051180

1. Bed-side blood pressure measurement (10 minutes): Three blood pressure readings will be taken at intervals of 1 minute from your left arm (unless there is a medical reason not to use the left) using an automated blood pressure monitor. This will require you sitting at rest for 5 minutes prior to doing any measurements.
2. Echocardiogram scan (15 minutes): We will perform an ultrasound (echocardiogram/echo) of your heart. This is a safe and painless procedure and you will be asked to lie on a couch on your left side. A probe is placed on your chest and lubricating jelly is used so the probe makes good contact with the skin. Ultrasound waves then create images of your heart on the scanner monitor. It normally takes 15 minutes to acquire these images. A female sonographer/scanner will be provided wherever possible and if not available, a female chaperone will be available.
3. Vicorder® (Vascular Measures and Central Blood Pressures, 10 minutes): This involves lying flat on a couch and having two blood pressure cuffs fitted, one to the right arm and one to the right leg. These are inflated and deflated three times at 1-2 minute intervals. This is now an optional measure during the COVID-19 pandemic.
4. Lifestyle and diet questionnaire (25 minutes): The questionnaire combines validated questions used in previous studies. Information will be collected on factors that affect blood pressure including: smoking frequency, alcohol and salt intake, exercise and family history. Questionnaires can be completed either during a study visit or at a later date and posted back to the study team (pre-paid envelopes will be provided). Some of these questions may not seem relevant as they are taken from validated questionnaires used across a range of ages and in both males and females. The team will explain any such questions.
5. EQ-5D-5L Quality of Life questionnaire (5 minutes): You will be provided with an EQ-5D-5L questionnaire, a widely used and validated way of assessing quality of life. A trained study investigator will run through the structured questionnaire during the visit with you.
6. MRI of the Heart, Brain, aorta and kidneys (1 hour including break): As part of this study you will have an MRI scan of your heart and brain. The MRI scanner is shaped like a polo mint, the hole inside measuring about 60 centimetres wide. MRI is safe and non-invasive and does not involve any ionising radiation (x-rays). However, because they use a large magnet to work, MRI scans are not suitable for everybody. Because of this, you will be asked pre-screening safety questions to help determine if you are able to take part. More detail about the MRI scan is provided on the *Supplementary Information Sheet* given to you before the 4th study visit. This additional information sheet also provides more detail about the optional sub-study of having gadolinium given (a commonly used contrast drug for MRI) to acquire an extra few images if you are no longer breast-feeding at that time-point. Additional consent will be sought and obtained for this sub-study prior to the scan being performed.
7. Blood test (10 minutes): The equivalent of 5 teaspoons of blood will be collected by a trained member of staff. We will try our best to time it with blood tests requested by your clinical care team whilst you are an inpatient. You do not need to be fasted for this test. We use very small

'butterfly' needles to minimise any discomfort. You may experience some bruising and discomfort at the site where you have your blood taken. Our staff are highly trained in blood taking and we will make sure you are as comfortable as possible.

8. **Fitting of a home 24 hour blood pressure monitor (5 minutes, to be worn for 24 hours) will be done only during V3 at week 6 and during the final visit (V4). The activity monitor (to be worn for 7 days and nights) is only fitted during V4**:
   The 24hr monitor consists of a blood pressure cuff, which will be fitted on the left arm (right if a specific medical reason precludes use of the left) and a BP monitor. A small bag will also be provided that is worn around the waist or shoulders, in which the monitor is placed. You will be shown how to re-attach the cuff to the monitor e.g. after a bath/shower. The BP monitor will be silent and automatically inflate hourly during the day and every other hour at night to minimise inconvenience at this busy time. Participants will be asked to wear the monitor for 24 hours. We provide you with an information sheet about how to use the monitor and deal with frequent problems and this also has a brief blood pressure monitoring diary on the back for you to detail the time you went to sleep/woke up and any periods of activity that may have put your blood pressure up e.g. running for the bus/cycling to work. The activity monitor (wrist-watch) is waterproof and shock-proof and will be worn continually on the wrist for one week. Stamped, addressed envelopes will be provided to return both devices after use.
9. Retinal imaging (10 minutes): Photos will be taken of the back of your eye just like at an optician. More information about this is provided in the *Supplementary Information Sheet*. To help keep your information confidential, your images will be 'de-identified' and assigned a study code. However, your retinal images are unique to you so they can never be completely anonymous.
10. Cardiopulmonary Exercise Testing (CPET) with exercise echo (30 minutes): This involves gentle cycling on a stationary bicycle and doing a short ultrasound (echocardiogram) of your heart whilst exercising. More information about this is provided in the *Supplementary Information Sheet*.

The Self-management (intervention) group: Taking your blood pressure at home

This section only applies to participants who are allocated to the intervention group and an additional intervention group information sheet will be provided for you as a paper copy, on the app and on the website.

You will be asked to **start measuring your blood pressure** on the **day of discharge** from hospital using the OMRON Evolv monitor provided. This will mean taking **2 readings, 1 minute apart** every morning after discharge. **We will ask you to do this every morning, until you have had 5 consecutive days**

with blood pressure readings in the normal range (off medication). We anticipate this to take approximately **2-3 weeks following discharge** based on our experience in our pilot study.

After you measure your blood pressure, you will **open up the POP-HT app** on your smartphone/tablet, confirm it was you that took the blood pressure readings and **click 'SYNC'.** The app will then 'synchronise' with the OMRON EVOLV® monitor to **upload the reading to the app and the secure study website** hosted on the hospital's secure intranet (*not the internet*). **See figure one below for an illustration of how this is done**

Your smartphone/tablet will notify you if your readings are too high, too low or in the normal range. If they are high or low the app will ask you to repeat one more reading. If the reading is still high/low the app will notify you and the specialist study doctors. **You can then opt to receive a call from them to help adjust your medication and will be advised to make an urgent/same-day appointment with your own GP/midwife if necessary** (further detailed information about this is available in the self-management information sheet). Medication will be gradually titrated down until you are off all medication.

Once you have 5 days in a row with readings in the normal range (off medication) you will be sent a notification/message reading "Thank you. Your blood pressure readings have all been normal since stopping treatment. Please **change to once weekly readings** for the remainder of the study." This will be **until 6 months** after the delivery of your baby. The **reason** for the **longer period of weekly monitoring** is to **ensure you do not have a late rise in your blood-pressure readings** that requires further treatment.

Figure 1: Illustration of how self-monitoring will be performed in the intervention group

Kitt J, *et al. BMJ Open* 2022; 12:e051180. doi: 10.1136/bmjopen-2021-051180

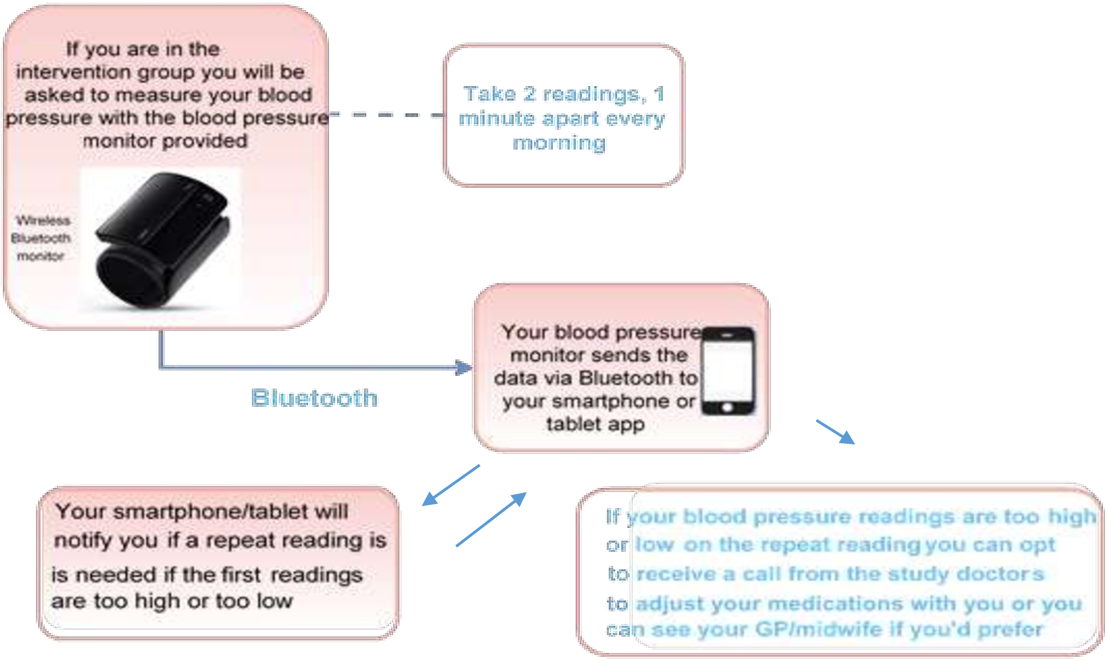

The control group

If you are allocated to the control group, you will be looked after by your GP/midwife/Health visitor as per usual NHS care in addition to the 4 study visits described above. **You will also be given a blood pressure monitor for the first few weeks, during the COVID-19 pandemic, as per updated national guidance**. This is to facilitate care from your GP and mid-wife who may not be able to do home visits as they normally would during the pandemic. Instead, your self-monitored readings can be reviewed by a tele-medicine appointment (when a face-face review is not possible due to COVID-19) and your medications adjusted as needed.

What will I need to do if I want to take part?

You will have plenty of time to consider your participation after reading this. If you decide you want to take part, the first step is to contact the research team who will arrange your first study visit.

Contact: Dr Jamie Kitt or Mrs Yvonne Kenworthy Tel: 01865 572833 or study mobile 07713 782185

E-mail: Jamie.kitt@cardiov.ox.ac.uk or Yvonne.Kenworthy@cardiov.ox.ac.uk

You can contact us by calling/SMS/e-mail to arrange a study visit, or we will contact you after giving you at least one hour to consider this document. If you are willing to participate in the study, then we will take your written consent.

Chief Investigator:	Prof Paul Leeson	Version/date:	6.0 23.03.2021
Study Short Title:	POP-HT STUDY	Ethics Ref:	19/LO/1901
		IRAS ID:	273353
Document:	Participant Information Sheet		

What should I consider?

You will not be able to take part in this study if the chief investigator deems your taking part would be unsafe and will already have been screened against strict exclusion criteria. Should you have any concerns however, please discuss this with the study team. Due to the fact that the nature of this research study involves home visits, all members of the research team have undertaken the relevant and stipulated safe-guarding training.

Are there any possible disadvantages or risks from taking part?

Your decision on whether you will participate or not **will not affect in any way your clinical care** now or in future. All of the study procedures/assessments are safe but as with any medical procedure, there are some minor risks. More details on the risks associated with certain procedures e.g. cardiac MRI and blood taking are detailed in the ***Supplementary Information Sheet provided with this main PIS***. At all times, an experienced study investigator will be with you and will address any issues that may arise. As per routine clinical advice women in both arms of the trial should continue to monitor their babies for drowsiness, lethargy, pallor, cold peripheries or poor feeding when discharged home.

What are the possible benefits of taking part?

This study is designed to test whether self-management can improve blood pressure control in the period immediately after birth. As part of the study all participants receive 4 additional visits above and beyond usual NHS care and so will be more closely monitored than you otherwise would be. This may result in earlier access to treatment should any abnormal BP readings be detected.

Will my taking part in this study be kept confidential?

Yes. The data collected from the study will be de-identified so that you will be known only by a unique study specific ID. You would not be identifiable from this. Responsible members of the University of Oxford, Oxford University Hospitals NHS Trust and Regulatory Authorities may be given access to data for monitoring and/or audit of the study to ensure that the research is complying with applicable regulations. As part of our commitment to maximise participant involvement in research, participants can give consent (optional) for their contact details to be retained (see section below). As mentioned above, details will be shared with NHS digital to allow longer-term follow up of your blood pressure up to 10 years. NHS digital is regulated by the same strict criteria as this study. In the event that you lose capacity to consent whilst taking part in the study you will be withdrawn from the study and no further data will be collected nor any further assessments/procedures undertaken. Any data that has already been collected will be retained.

What will happen to the samples I give?

Blood samples collected will be analysed for this study, but your samples may also be used for other studies with appropriate ethical approval in the future. Samples collected will be de-identified and stored in secured facilities within the University of Oxford. If you withdraw from the study for any reason, we will retain any

blood samples and data collected up to that point for use in research as detailed in this participant information sheet. If you agree to your samples being used in future research, your consent form will be held until the samples have been depleted or destroyed.

Will my General Practitioner/family doctor (GP) be informed of my participation?

Your GP will be notified of your study participation and will be provided with a letter or study information sheet. There may also be instances where GPs will be contacted to follow up incidental findings that may be of clinical significance or if you withdraw/are withdrawn from the study.

What will happen to my data?

Data protection regulation requires that we state the legal basis for processing information about you. In the case of research, this is 'a task in the public interest.' The University of Oxford is the data controller and is responsible for looking after your information and using it properly. We will be using information from you, your medical notes and NHS Digital (and other central NHS bodies) in order to undertake this study and will use the minimum amount of personally-identifiable information possible. We will store any research documents with personal/traceable data, such as consent forms and your retinal images, securely at the University of Oxford for 10 years after the end of the study as part of the research record. If you have consented to your samples being retained for future research, a copy of your consent is retained for the duration of sample storage. We keep any other identifiable information about you for up to 12 months after the study is finished. All documents containing personal information such as your informed consent form will be stored securely and only accessible by study staff and authorised personnel only. The Oxford University Hospitals NHS Foundation Trust will use your name, NHS number, date of birth and contact details (address and telephone number) to contact you about the research study, to make sure that the relevant information about the study is recorded for your care, and to oversee the quality of the study. They will keep identifiable information about you from this study for up to 12 months after the study has finished. As part of your participation in the study, in addition to the information you provide about your health, the research team in Oxford will ask for information about your health from NHS Digital (including, but not limited to, NHS Digital). We will send your name, date of birth, NHS number and postcode to NHS Digital (or other central NHS bodies) who can link this information to your centrally held records. Data protection regulation provides you with control over your personal data and how it is used. When you agree to your information being used in research, however, some of those rights may be limited in order for the research to be reliable and accurate. Further information about your rights with respect to your personal data is available at http://www.admin.ox.ac.uk/councilsec/compliance/gdpr/individualrights. You can find out more about how we use your information by contacting jamie.kitt@cardiov.ox.ac.uk.

Chief Investigator:	Prof Paul Leeson	Version/date:	6.0 23.03.2021
Study Short Title:	POP-HT STUDY	Ethics Ref:	19/LO/1901
		IRAS ID:	273353
Document:	Participant Information Sheet		

Will I be reimbursed for taking part?

If you visit us at CCRF we will reimburse travel and parking expenses to and from the CCRF at the John Radcliffe Hospital site, if you provide receipts and/or mileage details. **You will also receive £30 thank-you** for your participation in the study after the final study visit. The app notifications and usage are free when in WIFI zone but if using 3G/4G they may be charged depending on your network-provider. If this is the case, then any cost incurred will be reimbursed to you on production of the relevant bill.

What will happen if I don't want to carry on with the study?

You can withdraw from the study at any time without giving a reason. If you decide you no longer wish to take part in our study, you can phone, write to, or e-mail Dr Jamie Kitt using the contact details listed in the header on page one. Should you wish to withdraw, please let us know if we can keep the information we have collected about you so far as we may be unable to destroy the data if it has already been de-identified, as outlined in the confidentiality section. Data and samples already collected would not be used in the final study analysis except where analysis of their data or samples has already been integrated into interim results.

What will happen to the results of the study?

Summarised results will be published in scientific journal/s and also summarised on our website, after completion of the study, for you to read: https://www.rdm.ox.ac.uk/about/our-clinical-facilities-and-mrc-units/cardiovascular-clinical-research-facility/ongoing-clinical-studies.

What if we find something unexpected?

If your blood pressure readings are very high (**above 160/110mmHg**), we will inform you of this finding immediately with instructions on what to do next and we will also call you to inform you to seek medical assistance. In the unlikely event that we detect any structural abnormalities during the scan of your heart (echocardiogram) or MRI of your heart or brain, then with your permission we will refer you for assessment by contacting your GP and/or a specialist hypertension clinic, who can arrange instigate any necessary investigations and treatment.

What if there are any problems?

The University of Oxford, as Sponsor, has appropriate insurance in place in the unlikely event that you suffer any harm as a direct consequence of your participation in this study. NHS indemnity operates in respect of the clinical treatment which is provided. If you wish to complain about any aspect of the way in which you have been approached/treated during this study, you should contact, the Chief Investigator, Prof Paul

Leeson on +44 (0)1865 572846 or e-mail: paul.leeson@cardiov.ox.ac.uk. You may contact the University of Oxford Clinical Trials and Research Governance (CTRG) office on 01865 616480, or the head of CTRG, email ctrg@admin.ox.ac.uk.

How have patients and the public been involved in this study?

Members of the public, who have been through similar pregnancy related medical problems, have been involved in the design of this study, testing and refining of the intervention arm being trialled and several of the documents including the poster, logo and this patient information leaflet.

Who is organising and funding the research?

This study has been designed and organised by investigators of the University of Oxford, Division of Cardiovascular Medicine and Nuffield Department of Primary Care Health Sciences (namely Prof Paul Leeson, Dr Jamie Kitt, Professor Richard McManus, Dr Lucy Mackillop and Dr Adam Lewandowski). If you wish to know more about any aspect of the study, please contact Jamie Kitt on 01865 (5)72833 or jamie.kitt@cardiov.ox.ac.uk. Dr Jamie Kitt is conducting this research as part of his doctoral studies and results from this study may be used in anonymous form to support this. The research is being financed by the British Heart Foundation. The sponsor of the study is the University of Oxford.

Who has reviewed the study?

All research in the NHS is looked at by an independent group of people called a Research Ethics Committee, to protect your safety, rights, wellbeing and dignity. This study has been reviewed by London Surrey REC [19/LO/1901].

Participation in future research:

If you consent to be considered for future studies, a copy of this consent and your contact details will be kept securely and independently of the study records in a separate, secure database in order that we can contact you if further research in this area is being under-taken. You are under no obligation to consent to being contacted again and we understand you may want to participate only in this study in which case your details will not be kept as explained in the data confidentiality section above. You have the right to ask for your personal information to be removed from this database at any time.

Further information and contact details:

Lead Study Investigator: Dr Jamie Kitt; Tel: 01865 572833 Email: jamie.kitt@cardiov.ox.ac.uk

Chief Investigator: Prof Paul Leeson; Tel: 01865 572846 Email: paul.leeson@cardiov.ox.ac.uk

Amendments during COVID-19 pandemic

Chief Investigator:	Prof Paul Leeson	Version/date:	6.0 23.03.2021
		Ethics Ref:	19/LO/1901
Study Short Title:	POP-HT STUDY	IRAS ID:	273353
Document:	Participant Information Sheet		

During the COVID-19 pandemic, this PIS and the flyer will be provided electronically via a tablet, which the clinical team will give you, although there will be less time to consider this than normal due to expedited discharge processes during the pandemic. Consent forms will be placed in wipe down wallets, which will be handed to you for signing and then photocopied whilst wearing gloves. The copy will be placed back into a sterile wallet for you and the original will be placed in a second wallet. Both will be wiped down with CLINELL® wipes and our copy will be kept securely in quarantine before moving them to CCRF. The baseline visit has been adjusted to reduce the amount of direct patient contact to the blood pressure measurements, the ultra-sound (echo), and the Vicorder test is now optional. There will not be a second female chaperone from the research team (this will not affect normal hospital chaperone rules). Questionnaires are e-mailed out and can be completed on a tablet/computer at a later date. All direct contact will be done in PPE where necessary in line with hospital policy. The control arm will also be provided with a blood pressure monitor, when NHS monitors are not available prior to discharge to allow home monitoring by NHS GPs/mid-wives in line with updated RCOG guidance. The study will be performing remote follow up visits for week 1 and week 6 (visits 2 and 3). The final (4th visit) is being extended from 6-9 months, to 6-12 months by which time 'normality' will hopefully have been resumed to allow the final visit to take place at the John Radcliffe Hospital. In extenuating circumstances, such as COVID-19 national lockdowns some part of the V4 will also be done remotely, the 24hr BP monitor fitting, the accelerometer/wrist-watch fitting, the review of the medical history, demographics, questionnaires, and manual blood pressure measurements may also be conducted remotely via video or phone call. The remaining procedures of the final visit will be done when it is safe to do within the 12 month time-window.

Tel: 01865 572833
Email: jamie.kitt@cardiov.ox.ac.uk
Fax: +44(0)1865 572840

Study Code: *Participant identification number:*

P	O			

Physician Optimised Post-partum Hypertensive Treatment (POP-HT) Study

CONSENT FORM

Name of Researcher:

Participant Name:

If you agree, please initial each box

1. I confirm that I have read the information sheet dated V3.0 06/05/20 for this study. I have had the opportunity to consider the information, ask questions and have had these answered satisfactorily.	
2. I understand that my participation is voluntary and that I am free to withdraw at any time without giving any reason, without my medical care or legal rights being affected.	
3. I understand that relevant sections of my medical records and data collected during the study may be looked at by individuals from University of Oxford, hosting NHS organisations and regulatory authorities, where it is relevant to my taking part in this research. I give permission for these individuals to have access to my records.	
4. MRI/Echocardiography/other research tests: I understand that these are research scans/tests that are not useful for medical diagnosis, and that scan/test results are not routinely looked at by a doctor. If a concern is raised about a possible abnormality on my scan/research test, I will only be informed if a doctor thinks it is medically important such that the finding has clear implications for my current or future health.	
5. I agree for my GP to be informed of any results of medical tests performed as part of the research that may be important for my health care.	
6. If I withdraw/I am withdrawn from the study we will contact your GP to inform them, in order to ensure that any on-going care you require is reinstated.	
7. I agree that the information held and maintained by NHS Digital/ Office for National Statistics (ONS) may be used to provide information about my health	

Chief Investigator:	Prof Paul Leeson	Version/date:	6.0 23.03.2021
Study Short Title:	POP-HT STUDY	Ethics Ref:	19/LO/1901
		IRAS ID:	273353
Document:	Participant Information Sheet		

status. I understand that my name, NHS number, date of birth and postcode may be shared securely to obtain such information and allow contact for blood pressure, and other relevant measurements over the next 10 years.		
8. I agree to donate blood samples. I consider these samples a gift to the University of Oxford and I understand I will not gain any direct personal or financial benefit from them.		
9. I understand that retinal images will be taken as part of the study and will be stored in a de-identified format on the high compliance (secure) server of the University of Oxford for up to 10 years		
10. I agree to take part in this study		
Additional:	Yes	No
11. I agree for my anonymised samples to be used in future research, here or abroad, which has ethics approval. I understand this research may involve commercial organisations.		
12. I agree to be contacted about ethically approved research studies for which I may be suitable. I understand that agreeing to be contacted does not oblige me to participate in any further studies.		
13. I have been approached about the optional additional measure: Vicorder ® assessment of the aortic stiffness and aortic blood pressure		
14. I have been approached about the optional additional measure: Laser Speckle Tracking of the skin on my fore-arm and agree to partake in this additional measure (being done in 48 participants out of 200)		

________________________	__________________	____________________________
Name of Participant	*Date*	*Signature*

________________________	__________________	____________________________
Name of Person taking Consent	*Date*	*Signature*

**** For researchers please tick (✓) to document:***

The original signed form will be placed in the medical notes and a further copy will be retained at the trial site and one given to the participant *( )*

Chief Investigator:	Prof Paul Leeson	Version/date:	6.0 23.03.2021
Study Short Title:	POP-HT STUDY	Ethics Ref:	19/LO/1901
		IRAS ID:	273353
Document:	Participant Information Sheet		

Chief Investigator:	Prof Paul Leeson	Version/date:	6.0 23.03.2021
Study Short Title:	POP-HT STUDY	Ethics Ref:	19/LO/1901
		IRAS ID:	273353
Document:	Participant Information Sheet		
