# Supplementary material for: Postpartum blood pressure self-management following hypertensive pregnancy: protocol of the Physician Optimised Post-partum Hypertension Treatment (POP-HT) trial
Source: BMJ Open. 2022 Feb 23;12(2):e051180. doi: 10.1136/bmjopen-2021-051180 (PMC8867381; doi:10.1136/bmjopen-2021-051180)
Supplement: Author's manuscript [file bmjopen-2021-051180.draft_revisions.pdf]

BMJ Open is committed to open peer review. As part of this commitment we make the peer review history of every article we publish publicly available.

When an article is published we post the peer reviewers' comments and the authors' responses online. We also post the versions of the paper that were used during peer review. These are the versions that the peer review comments apply to.

The versions of the paper that follow are the versions that were submitted during the peer review process. They are not the versions of record or the final published versions. They should not be cited or distributed as the published version of this manuscript.

BMJ Open is an open access journal and the full, final, typeset and author-corrected version of record of the manuscript is available on our site with no access controls, subscription charges or pay-per-view fees (http://bmjopen.bmj.com).

If you have any questions on BMJ Open's open peer review process please email info.bmjopen@bmj.com

BMJ Open

A Randomised control trial of post-partum blood pressure self-management following hypertensive pregnancy: Physician Optimised Post-partum Hypertension Treatment (POP-HT) trial protocol paper

Journal:	*BMJ Open*
Manuscript ID	bmjopen-2021-051180
Article Type:	Protocol
Date Submitted by the Author:	15-Mar-2021

Complete List of Authors:	Kitt, Jamie; University of Oxford<br>Frost, Annabelle; University of Oxford<br>Mollison, Jill; University of Oxford, Nuffield Department of Primary Care Health Sciences<br>Tucker, Katherine; University of Oxford, Nuffield Department of Primary Care<br>Suriano, Katie; University of Oxford<br>Kenworthy, Yvonne; University of Oxford, Oxford Cardiovascular Clinical Research Facility<br>McCourt, Annabelle; University of Oxford<br>Woodward, William; University of Oxford<br>Tan, Cheryl; University of Oxford<br>Lapidaire, Winok; University of Oxford<br>Mills, Rebecca; University of Oxford, Radcliffe Department of Medicine<br>Khan, Maryam; University of Oxford, Radcliffe Department of Medicine<br>Tunnicliffe, Elizabeth M; University of Oxford, Radcliffe Department of Medicine<br>Raman, Betty; University of Oxford, Radcliffe Department of Medicine<br>Santos, Mauro; University of Oxford, Institute of Biomedical Engineering, Department of Engineering Science<br>Roman, Cristian; University of Oxford, Institute of Biomedical Engineering, Department of Engineering Science<br>Hanssen, Henner; University of Basel<br>Mackillop, Lucy; University of Oxford, Nuffield Department of Women's and Reproductive Health<br>Cairns, Alexandra; University of Oxford, Nuffield Department of Women's and Reproductive Health<br>Thilaganathan, Basky; University of London Saint George's, Obstetrics and Gynecology<br>Chappell, Dr Lucy; King's College London, Women's Health Academic Centre<br>Aye, Christina; University of Oxford, Nuffield Department of Women's and Reproductive Health<br>Lewandowski, Adam; University of Oxford, Division of Cardiovascular Medicine, Radcliffe Department of Medicine<br>McManus, Richard; University of Oxford, Dept of Primary Care Health Sciences<br>Leeson, Paul; University of Oxford, Division of Cardiovascular Medicine

Keywords:	Hypertension < CARDIOLOGY, Echocardiography < CARDIOLOGY, Cardiology < INTERNAL MEDICINE, Vascular medicine < INTERNAL MEDICINE, Maternal medicine < OBSTETRICS

SCHOLARONE™
Manuscripts

A Randomised control trial of post-partum blood pressure self-management following hypertensive pregnancy: Physician Optimised Post-partum Hypertension Treatment (POP-HT) trial protocol paper

Dr Jamie Kitt[1] BM BCh MA MRCP, Dr Annabelle Frost[1] MChB MRCOG, Dr Jill Mollison[5] PhD, Dr Katherine Tucker[5] BSc (Hons) PhD, Dr Katie Suriano[1] PhD, Mrs Yvonne Kenwothy[1] BSc, Mrs Annabelle McCourt[1], Mr William Woodward BSc[1], Mrs Cheryl Tan[1] BSc(Hons) MRes, Dr Winok Lapidaire[1] MSc PhD, Mrs Rebecca Mills MSc[6], Ms Maryam Khan BSc[6], Dr Elizabeth M Tunnicliffe[6] PhD, Dr Betty Raman[6] MBBS DPhil, Dr Mauro Santos[7] D Phil, Dr Cristian Roman[7] D Phil, Professor Henner Hanssen[8] MD, Dr Lucy Mackillop[2] BM Bch MA, Dr Alexandra Cairns BM BCh MRCOG[2], Professor Basky Thilaganathan[3] FRCOG, Professor Lucy Chappell[4] FRCOG, Dr Christina Aye[2] MRCOG DPhil, Dr Adam Lewandowski[1] BSc (Hons) DPhil, Professor Richard McManus[4] FRCGP PhD and Professor Paul Leeson[1] PhD FCRP
1 Cardiovascular Clinical Research Facility (CCRF), Division of Cardiovascular Medicine, University of Oxford, Oxford, UK
2 Nuffield Department of Women's and Reproductive Health, University of Oxford, Oxford, UK
3 Maternal-fetal Medicine Unit, St George's University Hospitals NHS Foundation Trust
4 King's College London
5 Nuffield Department of Primary Care, University of Oxford, Oxford, UK
6 Oxford Centre for Clinical Magnetic Resonance Research, Division of Cardiovascular Medicine, Radcliffe Department of Medicine, University of Oxford, Oxford, UK.
7 Oxford Institute for Biomedical Engineering, University of Oxford, Oxford, UK
8 Department of Sport, Exercise and Health, University of Basel, Switzerland

Correspondence to:
Dr Jamie Kitt, Oxford Cardiovascular Clinical Research Facility, Division of Cardiovascular Medicine, Radcliffe Department of Medicine, University of Oxford, John Radcliffe Hospital, Oxford. OX3 9DU, UK.
Tel: +44 1865 572833. Fax +44 1865 221111. E-mail: jamie.kitt@cardiov.ox.ac.uk

Date: 11/03/2021

Word Count: 6, 904 (excluding title page, abstract, figures, tables, references and appendices)

Protocol version: V 6.0 08/03/2021

Sponsor: University of Oxford Clinical Trials and Research Governance, Joint Research Office, 1st floor, Boundary Brook House, Churchill Drive, Headington. Oxford OX3 7GB Tel: 01865 61648
E-mail : ctrg@admin.ox.ac.uk

Funders: The research is being financed by a British Heart Foundation Clinical Research Training Fellowship (BHF Grant number FS/19/7/34148).

For peer review only

ABSTRACT

Introduction

New onset hypertension affects approximately 10% of pregnancies and is associated with a significant increase in risk of cardiovascular disease in later life, with blood pressure measured six weeks post-partum predictive of blood pressure 5-10 years later. A pilot trial has demonstrated that improved blood pressure control via self-management during the puerperium was associated with lower blood pressure for up to three years. POP-HT will formally evaluate whether improved blood pressure control in the puerperium results in lower blood pressure at six months post-partum, and improvements in cardiovascular and cerebrovascular phenotypes.

Methods and analysis

POP-HT is an open label, parallel arm, randomised controlled trial involving 200 women aged 18 years or over, with a diagnosis of pre-eclampsia or gestational hypertension, and requiring anti-hypertensive medication at discharge. Women are recruited by open recruitment and direct invitation around time of delivery and randomised 1:1 to either an intervention comprising physician-optimised self-management of post-partum blood pressure or usual care. Women in the intervention group upload blood pressure readings to a 'smartphone' app that provides algorithm-driven individualised medication-titration. Medication changes are approved by physicians, who review blood pressure readings remotely. Women in the control arm follow assessment and medication adjustment by their usual health care team. The primary outcome is 24 hour average ambulatory diastolic blood pressure at 6-9 months post-partum. Secondary outcomes include: additional blood pressure parameters at baseline, week 1 and week 6; multi-modal cardiovascular assessments (CMR and echocardiography); parameters derived from multi-organ magnetic resonance imaging including brain and kidneys; peripheral macrovascular and microvascular measures; angiogenic profile measures taken from blood samples and levels of endothelial circulating and cellular biomarkers; and objective physical activity monitoring and exercise assessment. An additional 20 women will be recruited after a normotensive pregnancy as a comparator group for endothelial cellular biomarkers.

Ethics and dissemination:

The Investigator will ensure that this trial is conducted in accordance with the principles of the Declaration of Helsinki and follow good clinical practice guidelines. The Investigators will be involved in reviewing drafts of the manuscripts, abstracts, press releases and any other publications arising from the study. Authors will acknowledge that the study was funded by the British Heart Foundation Clinical Research Training Fellowship (BHF Grant number FS/19/7/34148). Authorship will be determined in accordance with the ICMJE guidelines and other contributors will be acknowledged.

Trial Registration: Clinicaltrials.gov registration number (NCT04273854), registered 18TH February 2020.

Keywords: Blood pressure, Hypertension, Pre-eclampsia, Gestational hypertension, Hypertensive pregnancy, Randomised trial, Self-management, Cardiac imaging, Cardiac remodelling, Cerebrovascular health

For peer review only

STRENGTH AND LIMITATIONS OF THIS STUDY

- POP-HT is the first randomised trial that is powered to detect whether post-partum blood pressure self-management can significantly improve blood pressure control at 6-9 months post-partum, in women affected by new onset hypertension in pregnancy.
- POP-HT will also be the first randomised trial to investigate whether this improved blood pressure control translates into beneficial cardiovascular, vascular and cerebrovascular modelling, and will help elucidate the mechanisms behind adverse remodelling.
- The technology used to facilitate self-management in the study is readily translatable into widespread clinical practice, once it has been subject to the relevant regulatory approval and further validation required.
- The risk of drop-out, amplified by the COVID-19 global pandemic, could affect the ability to remain adequately powered to test the secondary outcome measures of the trial.
- The trial requires high levels of participant motivation and engagement during a busy time of the participants' lives. Patients in the intervention arm are required to submit daily readings when on treatment and adjust their medication as instructed by the app. Weekly readings are required thereafter for the duration of the study. This is in addition to the two periods of 24hr ABPM over the 6-month period. The degree of engagement required may limit recruitment into the trial and/or result in high drop-out/withdrawal rate.

For peer review only

ABBREVIATIONS

AE	Adverse event
ABPM	Ambulatory Blood pressure monitoring
AHA	American Heart Association
CI	Chief Investigator
CCRF	Cardiovascular Clinical Research Facility
CPET	Cardiopulmonary exercise test
CT	Clinical Trials
CTRG	Clinical Trials and Research Governance
DIC	Disseminated Intra-vascular Coagulation
DMC/DMSC	Data Monitoring Committee / Data Monitoring and Safety Committee
DSUR	Development Safety Update Report
ECV	Extra-cellular volume
ESC	European Society of Cardiology
GCP	Good Clinical Practice
GP	General Practitioner
HRA	Health Research Authority
ICF	Informed Consent Form
LV	Left ventricle
MHRA	Medicines and Healthcare products Regulatory Agency
NICE	National institute for clinical excellence
OCMR	Oxford Centre for Cardiovascular Magnetic Resonance Imaging
OIBME	Oxford Institute for Biomedical Engineering
NHS	National Health Service
NIHR	National Institute for Health Research
PI	Principal Investigator
PIS	Participant/ Patient Information Sheet
PPE	Personal Protective Equipment
PW	Pulse wave
R&D	NHS Trust R&D Department
REC	Research Ethics Committee
SAE	Serious Adverse Event
SOP	Standard Operating Procedure
TMF	Trial Master File

For peer review only

INTRODUCTION

Hypertensive disorders of pregnancy affect 10% of pregnancies, which equates to >80,000 women per year in the UK [1]. One study showed that 50% of women with pre-eclampsia have persistent significant hypertension on day five following delivery [2], with blood pressure control remaining an issue for up to six weeks after childbirth, often requiring multiple medications and careful titration. At the same time, competing demands on mothers, not least from her newborn baby, are associated with poor adherence and/or poor levels of clinical contact. Drug titration can therefore be sporadic exacerbating poorly controlled hypertension. The risk of poor blood pressure control during this period may extend into later life. Hypertensive disorders of pregnancy are associated with a two-fold increase in risk of subsequent cardiovascular disease [3, 4] with a third presenting with chronic hypertension within 10 years [5]. We observed that high blood pressure during the first six weeks post-partum is related to the risk of higher blood pressure in the five to 10 years following a hypertensive pregnancy [6]. Furthermore, the SNAP-HT randomised controlled pilot study [7] showed blood pressure control in the post-partum period can be improved through self-management, with a mean improvement of 5.2mmHg in systolic and 6mmHg in diastolic blood pressure at six weeks post-partum. However, the most striking finding was that mean diastolic blood pressure remained 4.5mmHg lower in the intervention group at six months' post-partum; even after all but two mothers had stopped medication. Long-term follow-up of the women involved in the trial demonstrated diastolic blood pressure remained persistently lower in the intervention group for up to three years post-partum (*under review)*.

In the general population, high blood pressure is strongly related to long-term risk of cardiovascular disease and is the leading risk factor for loss of disability-adjusted life years (DALYs) in high and low-middle income countries. Every 10 systolic/5 diastolic mmHg of blood pressure reduction associates with a ~40% reduction in lifetime stroke risk and ~20% reduction in coronary heart disease risk [8]. If the difference observed in SNAP-HT could be translated to all women who have a hypertensive pregnancy, significant reductions in disease burden could be achieved in the population. However, several questions remain to determine the potential clinical translational benefits of self-monitoring in the management of post-partum blood pressure. Firstly, can a similar reduction in blood pressure be achieved with updated technology in a trial powered to detect diastolic BP differences at 6 months? Bluetooth-enabled home blood pressure monitors allow for automated upload of readings to the app, reducing the need for manual entry during a busy period in the patients' lives. This also facilitates telemonitoring by physicians who can review and advise on readings [9-11]. Secondly, does improved post-partum blood pressure control also result in reduced end organ damage in the cardiac, vascular and cerebrovascular systems? Hypertensive pregnancies are associated with early changes in cardiac, vascular and brain structure and function, which are disproportionate to their cardiovascular risk profile [12-14]. This may explain why this population have an increased risk of later cardiovascular and cerebrovascular disease. It is possible these changes may emerge during pregnancy and persist long-term, independent of post-partum blood pressure variability. Although the significant cardiovascular adaptations that emerge during pregnancies complicated by hypertensive disorders of pregnancy are known to reverse to some extent during the post-partum period [15-18], an alternative hypothesis it that the long-term risk reflects a failure of the cardiac, vascular or cerebral systems to 'normalise' after pregnancy [19, 20]. If so, improved post-partum blood pressure control may offer a new approach to modify these long-term end organ changes, in addition to any beneficial effects on blood pressure.

For peer review only

HYPOTHESES

The primary hypothesis is that blood pressure self-management with clinician oversight will reduce diastolic blood pressure at six to nine months post-partum; in women requiring anti-hypertensive medication in the puerperium after a hypertensive pregnancy.

The secondary hypotheses are that blood pressure lowering in the puerperium and strict control within predefined target ranges during this time period will improve cardiovascular, and cerebrovascular and vascular phenotypes in this cohort including:

- MRI indices of cardiovascular structure & function,
- MRI indices of cerebral perfusion, white matter integrity, subcortical volumes,
- MRI assessment of aortic compliance,
- Echo measures of cardiovascular structure and function, especially diastolic function and left atrial volume.
- Improved cardiovascular adaptation to exercise, assessed by exercise ejection fraction during cardiopulmonary exercise testing (CPET).

  In addition, it is hypothesised the intervention will lead to improvements in peripheral vascular function, including integrity of the retinal vasculature and markers of vascular stiffness. The tertiary hypotheses are that self-management will be associated with higher quality of life scores once discharged from hospital, fewer post-natal readmissions to hospital, improved endothelial function; and fewer signs of kidney injury and fibro-inflammation.

METHODS

Study design

POP-HT is a single centre, open label, two-arm parallel, randomised controlled trial in women who develop hypertensive disorders of pregnancy, who require anti-hypertensive treatment at the time of discharge. This study will investigate the effectiveness of post-partum physician assisted self-management of blood pressure compared to standard care over the first six months post-partum.

We will recruit 200 participants, who will be randomly allocated in a 1:1 fashion to either the intervention or control arm. The intervention arm will comprise app-based home blood pressure monitoring (including periods of home 24hr ABPM) coupled with physician-assisted self-management. The control arm will receive 'standard' levels of NHS care from their GP and midwives and health visitors. All participants will be recruited from the Oxford Women's Centre at the John Radcliffe Hospital, which sees approximately 25 patients with hypertensive disorders of pregnancy per month.

A trial flow chart is presented in appendix A and a schedule of procedures in appendix B. The expected duration of participant involvement will be up to 12 months. Participants will attend four study visits after pre-screening and enrolment: baseline, week 1, week 6 and at 6-12 months.

Endothelial Cell Sub-study

The purpose of the sub study of 20 women is to provide a reference population of women not affected by hypertensive disease. In the sub study, 20 healthy postnatal women will undergo measurements of specific characteristics of blood cells and circulating factors involved in inflammation and endothelial dysfunction. This small sub-cohort population will validate how

For peer review only

blood cells and circulating factors vary naturally and may be affected by external factors such as mode of delivery and blood pressure. These 20 normotensive participants will be compared to 20/200 participants who have had a hypertensive pregnancy and that are in the main study. These 20/200 will provide additional consent for blood sampling for endothelial cells at baseline and at 6-12 months post-partum. The 20 normotensive patients will be recruited from the postnatal ward in the Oxford Women's Centre and the normotensive participants will be recruited directly to the sub-study and, not be expected to participate in the main POP-HT study. (See appendix C for a schedule of procedures for the sub-study).

Study intervention

The intervention is the provision of a wireless Bluetooth® enabled OMRON Evolv® blood pressure monitor, validated for widespread clinical use, including in pregnancy [29], alongside the installation of a proprietary smartphone app. The app will assist the self-management of post-partum blood pressure, with physician oversight of medication adjustment. Following the baseline visit, those randomised to the intervention arm will be provided with an OMRON Evolv® monitor and the app installed on the participant's phone and its use demonstrated. The participants will have ample time to become familiar with the device and app prior to discharge, as in SNAP-HT [4]. They will also be provided with an 'intervention-arm information sheet,' which contains information about the self-monitoring process as well as a frequently asked questions (FAQ) section and contact number for any technical issues. The system was developed by the Oxford Institute of Biomedical engineering (IBME) who have successfully developed apps for several other blood pressure studies including SNAP-HT [7], TASMIN [21] and BUMP (NCT03334149). Participants in the intervention arm will be asked to start home blood pressure readings on the day of discharge. Figure 1 below illustrates the method of blood pressure self-management.

The medications prescribed to each participant at discharge will be decided by their clinical care team. The medication schedule will be uploaded to a secure NHS hosted web-based platform which syncs automatically with the app. This approach was trialled successfully in the SNAP-HT pilot trial [7] and this study will be using the same approach to develop these dose titration schedules based on discharge medication, but in line with the updated NICE guidance NG133 [9]. Following discharge from hospital, participants will be asked to upload their home blood pressure readings in a standardised manner. These readings are in turn uploaded to the secure NHS hosted web-based platform. The readings are automatically cross-checked against pre-defined algorithms and an appropriate notification will be generated in response. Further explanation and illustrations of the study intervention are presented in appendix D.

Patient and public involvement(PPI)

The study team hosted regular PPI meetings prior to the study commencing to understand the experiences of patients participating in prior self-management and pre-eclampsia studies, and assist with study design and methodology. This included consulting participants and investigators from other related studies conducted by this group. Participant-facing information documents, were reviewed by PPI members with experience of raised blood pressure in pregnancy. PPI members also helped design the intervention to ensure it was acceptable for participants. The PPI group will also assist in the drafting of study results for dissemination to participants.

Study aims and objectives

	Objectives	Outcome Measures	Timepoint(s)
Primary	To compare post-partum diastolic BP in the intervention arm to the control arm.	24 hour average diastolic BP measured by assessed by SPACELAB 90217 24hr Ambulatory blood pressure monitoring (ABPM)	6-9 months post-partum
Secondary	To compare the effect of the intervention on cardiovascular, cerebrovascular and vascular phenotypes	**BP based**<br>a) 24 hr average systolic blood pressure assessed by SPACELAB 90217 24hr ABPM<br>b) Mean diurnal diastolic blood pressure assessed by SPACELAB 90217 ABPM<br>c) Mean diurnal systolic blood pressure assessed by SPACELAB 90217 ABPM<br>d) Mean nocturnal diastolic blood pressure assessed by SPACELAB 90217 24hr ABPM<br>e) Mean nocturnal systolic blood pressure assessed by SPACELAB 90217 24hr ABPM<br>f) Mean bedside diastolic blood pressure measured during study visit (mean of 2+3)<br>g) Mean bedside systolic blood pressure measured during study visit (mean of 2+3)<br><br>**Cardiac MRI**<br>h) Left ventricular (LV) mass indexed to end-diastolic volume and body surface area (BSA)<br>i) LV EDV indexed to BSA<br>j) LV wall thickness – septum, posterior and RWT<br>k) LA volume indexed to BSA<br>l) Right ventricular (RV) mass indexed to end-diastolic volume and body surface area (MRI)<br>m) RV EDV indexed to BSA<br>n) RA volume indexed to BSA<br>o) LV ejection fraction (EF) & RV EF<br>p) LV and RV stroke volumes indexed to BSA<br>q) Myocardial fibrosis<br>r) ECV (extra-cellular volume)	Week 6 and 6-9 months for the 24 hr ABPM<br><br>Baseline, week 1, week 6 and 6-9 months for the bedside blood pressures<br><br>For Cardiac MRI at 6-12 months post-partum

For peer review only

		**Echo**<br>s) LV Diastolic function: E/E' average, E/A ratio, E deceleration time<br>t) Global longitudinal strain (GLS)<br>u) LV systolic function (EF by Biplane Simpson's)<br>v) LA volume by Biplanar assessment<br><br>**Vascular**:<br>w) Pulse wave velocity<br>x) Augmentation index<br>y) Aortic BP<br>z) Aortic distensibility (MRI)<br><br>**Cerebrovascular**<br>aa) Total white matter hyperintensity volume<br>bb) Cerebral blood flow<br>cc) Mean vessel thickness of the middle and posterior cerebral arteries and internal carotid artery<br><br>**Retinal**<br>dd) the corrected central retinal arteriolar equivalent<br>ee) the corrected central retinal venular equivalent<br>ff) corrected central retinal arteriolar equivalent/corrected central retinal venular equivalent ratio.	At baseline and at 6-12 months post-partum for Echo outcome measures<br><br>PWV, Aortic BP and AI at baseline and at 6-12 months Aortic distensibility at 6-12 months<br><br>6 -12 months post-partum for all Brain MRI measures.<br><br>6-12 months post-partum for all retinal measures
Tertiary:	To explore in-vitro vascular function in	**Exercise Echo**<br>Exercise ejection fraction (echo) at 50% of peak workload during a bicycle cardio-pulmonary exercise test (CPET)<br>Exercise LA volume at 50% peak workload<br><br>**CPET**<br>VO2 at VT1<br><br>Assessment of endothelial cell function and circulating biomarker	6-12 months post-partum<br><br>6-12 months post-partum

For peer review only

	a sub-study of 20 women	levels associated with vascular angiogenesis and inflammation in normotensive and hypertensive women to determine if BP improvement can affect vascular function	From baseline to 6-12 months post-partum
	To explore presence/absence of kidney injury and fibro-inflammatory status	T1 mapping of the kidneys to look at cortico-medullary differentiation	6-12 months post-partum
	Quality of life assessment	EQ-5D-5L health questionnaire results	Baseline, week 1, week 6 and 6-12 months post-partum
	Participant experience: assessment of individual experience following intervention	Qualitative semi-structured interviews in subset of individuals	6-12 months post-partum
	Number of readmissions in intervention vs control arm	Readmission number in each arm	0-12 months post-partum
	Side-effect impact	Number and frequency of side-effects reported (intervention via the app and control during follow up calls/SMS)	0-12 months post-partum
Intervention(s)	The intervention will consist of physician-optimised self-management of post-partum BP. Women will follow a 'smartphone' app-based algorithm for medication titration, which will provide individualised dose titration advice. This is overseen and any change is approved by physicians who review the uploaded readings and respond to tele-monitored abnormal readings in a timely fashion.		
Comparator	The control arm will be managed as per usual NHS-led care with assessment by their own health care professionals and adjustment of medications as required. The BP of this group will be monitored and recorded at the same time-points and in the same manner as the intervention arm as with all other secondary outcome measures.		

For peer review only

Eligibility and recruitment

The procedures for each study visit and the estimated time each will take are listed in appendices B and C respectively.

All trial participants will be recruited locally from the Women's Centre at the John Radcliffe Hospital, Oxford. Screening will be carried out by the patient's clinical care team. Consent will be performed by the research team, once verbal consent is given to the clinical team for them to be approached.

Main Trial Participants

All participants will be females of childbearing age >18 years of age. Entry into the trial will require a clinician confirmed diagnosis of either gestational hypertension or pre-eclampsia defined by NICE NG 133 [9], that requires anti-hypertensive medication.

Inclusion Criteria

- Participant is willing and able to give informed consent for participation in the trial.
- Female, aged 18 years or above.
- Clinician confirmed diagnosis of either gestational hypertension or pre-eclampsia defined by NICE NG 133 and remains in hospital after delivery.
- Requiring anti-hypertensive medication at the point of discharge from secondary care.
- Participant has clinically acceptable laboratory results and clinical course post-partum with no other adverse complicating factor requiring prolonged admission post-partum that would make participation unfeasible as judged by the CI. Examples would include stroke sequalae, ongoing DIC, or other significant life-threatening co-morbidity
- In the Investigator's opinion, is able and willing to comply with all trial requirements including ownership of a smartphone or tablet and willing to use the smart-phone app if randomised to that arm.
- Sufficient competence in English language to follow the app instructions and partake in the study, as judged by the CI.

Exclusion Criteria (the participant may not enter the trial if ANY of the following apply):

- Significant renal or hepatic impairment that would affect safe medication titration and adjustment as part of the trial, as deemed by the Investigator.
- Participant with life expectancy of less than 6 months.
- Any other significant disease or disorder which, in the opinion of the Investigator, may either put the participant at risk through participation in the trial, influence the result of the trial, or impair the participant's ability to participate in the trial.
- Participants who have participated in another research trial involving an investigational product in the past 12 weeks.
- Women with pre-existing hypertension will be excluded, as this is a separate pathology that would affect the efficacy of the study intervention and affect the primary and secondary outcomes of the study.

ENDOTHELIAL CELLS SUB-STUDY

Dysregulation of the vascular endothelium and endothelial cells has been observed in the pathogenesis and progression of several cardiovascular diseases, including hypertension and hypertensive pregnancy disorders [22-28]. Studies have demonstrated that significant peripartum

For peer review only

inflammation, endothelial dysfunction and angiogenic imbalance extends beyond delivery, and persists to 5-10 years post-partum [29-31]. Endothelial colony-forming cells (ECFCs) represent a highly proliferative subtype of endothelial progenitor cells (EPCs), which play a vital role in the regulation of vascular homeostasis [32-34].
Herein, we investigate endothelial cell function using peripheral blood derived-ECFCs and angiogenic biomarkers in the blood at baseline and at 6-12 months post-partum

The inclusion criteria for the blood validation sub-study include:

- Participant is willing and able to give informed consent for participation in the trial.
- Female, aged 18 years or above.
- Normotensive (BP <140/90mmHg) throughout antenatal and postnatal period (except the 20/200 recruited from the main study)

The exclusion Criteria for POP-HT blood validation sub-study include:

- A hypertensive disorder of pregnancy (for the 20 normotensive recruits required)
- Use of beta blockers such as atenolol or equivalent
- BMI>35
- Evidence of cardiomyopathy, inherited cardiac conduction abnormalities, congenital heart disease or significant chronic disease relevant to cardiovascular status
- Folic acid or folate supplementation in the third trimester

Randomisation

Randomisation will be performed as soon as possible following the baseline visit. Randomisation will be carried out using a secure web-based randomisation software (embedded within Castor®). Participants will be randomised on a 1:1 basis and two minimisation factors will be used to ensure that the groups are matched as well as possible:

- Primary factor: Gestational age at the time of presentation with pre-eclampsia/gestational hypertension (agreed on as a surrogate marker of disease severity).
- Secondary factor: Prescription of ACE inhibitor (Enalapril) at the time of randomisation.

The full trial protocol (available on request) details more information on allocation concealment and implementation of the randomisation.

Assessments during study visits

A flow chart of the proposed study visits is included below (figure 2). All data will be recorded directly into CASTOR® electronic data capture forms where possible in real-time. Any data requiring post-processing will be entered into CASTOR following such analysis. During the study visits, all procedures are performed on participants in both the intervention and control arms with the exception of provision of the intervention; which is reserved only for those randomised to that arm.

As figure 2 illustrates, there will be ‘essential’ components to each study visit. However, as the research is being carried out on women with newborn babies, some components have been

For peer review only

classed as 'desirable' to allow for shortening of the study visits, if required, without affecting the primary outcome. **A number of modifications to the original study design,** and the means of performing the above assessments, have also been made to mitigate the impact of the COVID-19 global pandemic (**please see appendix E for further detail** in the supplementary file).

Baseline visit (week 0):

Demographics and anthropometry

Assessment will include recording of the ante-natal booking height, weight and BMI (obtained from notes) and the mid-left arm circumference.

Bed-side Blood pressure measurement

Participants will have their blood pressure checked after 5 minutes' rest using the automated mode of a validated sphygmomanometer. Three blood pressure readings will be taken at intervals of 1 minute. The measurement technique advised by the British Heart Foundation and NICE NG133 will be strictly followed.

Echocardiogram (cardiac ultrasound) scan

Cardiac ultrasound imaging will be performed by a trained sonographer to evaluate cardiac structure and function. British Society of Echocardiography guidelines will be followed for collection of a standard clinical imaging dataset

Collate data from medical notes and review blood results

A study team member will review the medical notes (paper and electronic) to document relevant medical history as stated in the full trial protocol

Quality of Life questionnaire (EQ-5D-5L)

Participants will be provided with an EQ-5D-5L questionnaire via e-mail

Desirable: Vicorder® (Vascular Measures and Central Blood Pressures)

Resting measures of vascular stiffness including pulse wave velocity and central blood pressure will be collected using a non-invasive device (Vicorder®).

Desirable: Lifestyle and diet questionnaire

The questionnaire, sent via e-mail, combines validated questions piloted or used in previous studies. Information will be collected on factors that affect blood pressure including: smoking frequency, alcohol and salt intake, exercise and family history.

Intervention provision: Automated blood pressure cuff provided and POP-HT app installed (those randomised to intervention arm only)

At the end of the baseline visit, those individuals that are randomised to the intervention arm will be issued with an OMRON EVOLV® automated blood pressure cuff (validated for use in pregnancy [35]). They will also download the POP-HT smartphone app and their use will be demonstrated. The participant will then have the remainder of their stay in hospital to practice. This is to ensure all parties are confident and competent prior to discharge home, at which point the intervention will start. Participants will be able to contact the study team via telephone or email for any technical problems.

For peer review only

Control arm during COVID-19: During the COVID-19 pandemic, the RCOG recommends 'self-monitoring 2-3 times in the first week after discharge' for women who have had a hypertensive pregnancy. Therefore, those women allocated to the control arm, who are unable to obtain a monitor from the Oxford Women's centre/NHS service, will be provided with a validated BP home-monitoring device by the trial team to ensure they can adhere to this RCOG guidance during week one. These monitors will be provided to enable the control arm participants to monitor their own blood pressure and in turn liaise with their own GP/mid-wife to adjust their management based on their readings. The study team will not be offering remote management to the control arm or providing them with an app, interpretation of the readings and management decisions are to be taken by their own GPs/clinicians. These monitors will also be used to allow remote BP measurements during the study visits at weeks one and six.

Subsequent Visits

- During the week one and six follow-up, the research team will perform all procedures for participants in both arms. As a result of the COVID-19 pandemic, all aspects of these follow-ups will be conducted remotely via video (and/or phone call).

Visits 2 and 3

Weeks 1 and 6 (± 5 days) post-discharge: blood pressure will be measured as per the baseline visits, up-to-date anthropometry will be measured, and an ED-5D-5L questionnaire will be completed.

Visit 3 only: 24hr ambulatory blood pressure monitor (to be worn for 24-hour period)

24-hour ambulatory blood pressure monitoring will be initiated at the end of the study visit using validated, calibrated, automated oscillometric, ambulatory devices (SPACELABS® 90217 or equivalent). Correct cuff size will be chosen based on arm circumference recorded at week 1 and 6.

Visit 4 (6 months (up to 12 months) post-partum

Participants will be invited to a final study visit. This is a more comprehensive visit with both essential and desirable components. A female chaperone will be offered and, where possible, all echocardiography will be performed by a female sonographer. In extenuating circumstances, such as COVID-19 national lockdowns, the 24hr BP monitoring and the procedures below can be conducted remotely via video call. Otherwise, these will be performed in person as described for the baseline visit.

- **Demographics and anthropometry:** Assessment will be performed as outlined above
- **Blood pressure:** Assessment will be performed as outlined above
- **Quality of Life questionnaire (EQ-5D-5L)**
- **Fitting of a 24 hour blood pressure monitor:** Assessment will be performed as outlined above
- **Fitting of an activity monitor:** For remote video calls, the accelerometer will be pre-programmed based on participant reported height, weight and hand dominance and then posted to the participant. For visits carried out in person, the accelerometer will be programmed during the visit, based on their height and weight recorded as part of the study visit and, fitted to their non-dominant wrist.
- **Review of medical and obstetric history and any medication side effects:** as described above

In cases where the above measures are performed remotely the additional procedures below, which cannot be done at remotely will be scheduled as soon as possible after, and within the 12-month time window defined in the protocol.

For peer review only

Echocardiogram cardiac ultrasound scan: will be performed as outlined above for baseline visit.
Vicorder® (Vascular Measures and Central Blood Pressure) assessment: will be performed as outlined above, but whilst in the MRI scanner, in order that the central/aortic pressure can be correlated with the aortic distensibility images obtained during the MRI scan.

Retinal imaging

Retinal photography of the right eye (3 single shot images centred on the optic disc) will be completed using a digital camera and imaging software following an established protocol.

MRI

A 3Tesla (3T) Siemens PRISMA scanner will be used to quantify brain structure and volume, followed by cardiac structure and function, cardiac mapping, measurement of aortic distensibility; and T1 maps of the kidneys.

Gadolinium contrast (optional): Gadolinium will be offered as an additional optional component to the MRI to those women who are not breast feeding, as part of exploratory work that may feed into a larger future trial. The additional PIS explains the Gadolinium procedure and risks/benefits in more details. Separate informed consent will be obtained for those women who wish to participate in this aspect of the study prior to the MRI being performed.

Blood: A venous blood sample (approximately 25mls) will be taken at rest and include samples for a) whole blood, plasma and serum lipid and inflammatory marker analysis and b) analysis of biochemistry and metabolism.

Cardiopulmonary Exercise Testing with exercise echo: Desirable

Cardiac function and oxygen requirements in response to an incremental increase in workload will be measured via a cardiopulmonary exercise test (CPET). The exercise protocol is a validated incremental protocol with established use in clinical and research practice. The exercise protocol is currently utilised in ongoing ethically approved studies conducted by the Division of Cardiovascular Medicine, and is performed on a stationary bike. The test commences with resting measures of spirometry. Participants will then exercise with an incrementally increasing workload up to 40-60% of their estimated peak exercise capacity. A brief focused echo will be performed at rest and at 40% of their maximal predicted exercise intensity (whilst on the bike) to enable measurement of exercise ejection fraction.

Sub- study Visits: Circulating biomarker validation and evaluation

20 of the hypertensive pregnancy patients from the main trial will also have a blood test taken at baseline as part of their main study visit. For the normotensive participants, the following study procedures and visits listed below remain separate to the main study. Study procedures will be the same during both visits.

The baseline visit will be carried out on the postnatal ward, in the Women's Centre, prior to discharge. Normotensive participants will be invited back for a second visit at the John Radcliffe Hospital between 6-12 months postpartum, and for those 20 hypertensive participants taking part in the main trial, the repeat blood test for the sub-study will be performed during the main V4/final visit when they have other study blood tests performed.

For peer review only

Procedures for all sub-study patients include: Demographics and anthropometry, Bed-side Blood pressure measurement and a blood test for analysis of biomarkers associated with inflammation, angiogenesis and endothelial activation as well as endothelial colony forming cells (ECFCs). Blood tests will be taken, where possible, at the same time as clinically-indicated venepuncture.

Data Analysis

Power calculations to determine adequate sample sizes for this trial are summarised below:

Primary outcome measure: **24-hour average diastolic blood pressure (mmHg) at 6-9 months post-partum as assessed by SPACELAB 90217 24hr Ambulatory blood pressure monitor**

Sample size calculation: The detection of BP differences between the 2 arms of this trial is based on the mean diastolic blood pressure difference detected in the pilot SNAP-HT study at 6 months. The mean BP difference detected between the intervention and control arm at the 6 month time-point was -4.5mmHg [7]. We have used a more conservative standard deviation (SD) of 10mmHg in each arm (in SNAP-HT the SD was 8.2mmHg in the intervention arm and 9.8 mmHg in the standard care arm) and 10mmHg SD is in keeping with pooled SDs for ambulatory diastolic blood pressure readings from other studies. To detect a treatment effect on diastolic blood pressure of -4.5mmHg, powered to 80% at p=0.05 requires a total sample size of 158 and with 1:1 randomisation this would require 79 in each arm. We have adjusted our power calculations to determine the final sample size, to allow for up to 20% loss to follow up/withdrawal based on prior experience. Thus, we aim to recruit 100 to the intervention and 100 to the control arm.

Secondary outcome hypothesis: Improved blood pressure control in the post-partum period (0-9 months) in POP-HT will result in improved cardiac, vascular and cerebrovascular phenotypes at 6-12 months post-partum

Secondary outcome power calculations

a) Cardiac structure*:* Studies using echocardiography by our collaborators have compared BP and LV mass in pre-eclampsia patients and control patients, at 1 year post-partum[14, 36][14, 37][14, 37][14, 36][14, 35] [14-15; 26, 36]. They found that a difference in BP at 1 year of 10mmHg in diastolic BP corresponded to significant differences in LV mass. SNAP-HT appeared to achieve a 50% reduction of anticipated BP difference seen between pre-eclamptic and normotensives at 1 year by 6 months i.e. ~5mmHg. If it is assumed that the structural/phenotypic benefit results from the BP benefit, as we are hypothesising, then we must power to detect 50% of the phenotypic difference. In previous work by our group we have demonstrated significant differences in LV mass/EDV (g/ml) in a similar age and predominantly female population with similar mean diastolic BP differences between groups to that seen in SNAP-HT. The LV mass/EDV (g/ml) in the group with high normal blood pressure was 1.54g/ml vs. 1.22 g/ml in those with optimal blood pressure with a standard deviation of 0.33 and 0.27 respectively at P<0.001. Based on these assumptions, to observe a treatment effect of 0.16 (50% of the difference between 1.54g/ml and 1.22g/ml) on LV mass/EDV, requires 67 in the intervention arm and 67 in the control arm (132 total). This is calculated using the larger SD of 0.33 referenced above at a power of >80% to detect a difference between the groups at p=0.05. This number should take into account for the greater dropout rate we may see for the MRI outcomes.

b) Brain White matter integrity*:* Work by our group, on pre-eclamptic pregnancy, showed an increased burden of temporal lobe white matter lesion volume 5-10 years after a pre-eclamptic pregnancy (23.2±13 µl) vs matched individuals who had a normotensive pregnancy (10.9 ± 11.5 µl) at p<0.05.If we again assume we can detect a 50% of the phenotypic benefit with our intervention as outlined above, we would anticipate a 50% reduction in the burden of white matter lesions i.e. 6.15 µl (50% of 23.2 – 10.9 µl) in the intervention arm. With 71 in the intervention group and 71 in the control group (142 total), this will provide >80% power at p=0.05, even using the more conservative SD of 13ul to detect a 50% improvement in white matter lesion volume between the intervention and the control group. This number should take into account for the greater dropout rate we may

For peer review only

see for the MRI outcomes.

c) Aortic compliance

Several studies assessing the impact of blood pressure on aortic compliance have shown that even modest reductions in systolic/diastolic blood pressure increase aortic distensibility/compliance. One such study had a mean difference in systolic blood pressure of 4.6mmHg between the 2 drug treatment arms at 52 weeks, akin to the same mean difference in SNAP-HT at 6 months, albeit this was diastolic not systolic, although other studies have suggested diastolic BP may be even more important in influencing aortic compliance. In this study with a mean 4.6mmHg difference in systolic BP there was a treatment difference of 0.12 [(95% CI −0.35, 0.60), *P* = 0.60 in aortic compliance. Based on these assumptions, to observe a treatment effect from our intervention, with 100 in the intervention and 100 in the control arm we will be more than powered at >90% to detect a difference at P=0.05 in POP-HT.

d) Exercise ejection fraction

Within our group, Huckstep et al compared resting and exercise ejection fractions for young adults with high normal BP vs. a normotensive cohort[37]. The cohort was well matched demographically to our planned study cohort, albeit it included both males and females. Resting ejection fraction (by Biplane Simpson's) was similar between groups but at 40%-60% of peak exercise intensity, the higher blood pressure group had a lower exercise ejection fraction than the normotensive cohort (73.9±3.25 vs. 80.0±4.54%, p<0.001) and in keeping with this, a smaller increase in ejection fraction when going from baseline to 40% exercise intensity (10.4±5.92 vs. 19.0±6.90%, p<0.001). Assuming the ~5mmHg mean BP improvement achieved in SNAP-HT again translates to a 50% phenotypic benefit, when assessing exercise ejection fraction we anticipate a 4.3% improvement in exercise stress ejection fraction in the intervention arm vs. the control arm (4.3% is 50% of the difference i.e. 50% of 10.4-19%). With 43 participants in the intervention arm and 43 in the control arm (86 total) we will be powered at >80% to detect such a difference at p=0.05. This calculation also takes account of the lower number likely to undertake the CPET at the final study visit.

Statistical Analysis Plan (SAP)

The statistical aspects of the study are summarised here with details fully described in a statistical analysis plan that will be produced in due course.

Description of Statistical Methods

The analysis will be carried out on the basis of intention-to-treat (ITT). This is, after randomisation, participants will be analysed according to their allocated intervention group irrespective of what treatment they actually receive. Patient demographic characteristics and other baseline information will be summarised by treatment group. Numbers (with percentages) for binary and categorical variables and mean (standard deviation), or median (interquartile or full range) for continuous variables will be presented. The analysis of the primary outcome will be assessed using a mixed effects model with baseline value, minimisation factors used in the randomisation process, randomised group and time will be fitted as fixed effects with a random intercept for each participant. Results will be presented as adjusted mean difference in change in mean ambulatory diastolic blood pressure between randomised groups at 6 months with 95% confidence intervals (CI) and associated two-sided p value. Secondary blood pressure outcomes will be analysed using the same method. Other secondary outcomes will be analysed using analysis of covariance (ANCOVA) to establish a co-variant model to examine the effect of blood pressure control in the post-partum period on cardiac structure and function, vascular function and cerebrovascular structure and function. If the model assumptions are not met and evidence

For peer review only

of departure from normality is observed, transformations of the data will be employed or non-parametric tests will be carried out.

Descriptive statistics (mean, standard deviation, standard error, range, etc.) will also be calculated for each outcome for each group. Differences in the primary and secondary outcomes will be compared between intervention and control groups.

Mean changes in blood pressures will be compared across the population and correlated with cardiovascular endpoints including cardiac structure and function reported from cardiac MRI and echocardiogram. Demographic and physiological characteristics of the participants will be added to regression models as covariates to explore the determinants of change in blood pressure comparing intervention and control groups.

Analysis Populations

The participants that will be included in the analysis will be all of those randomised. All data will be included in the analysis as far as possible to allow full ITT analysis, though there will inevitably be the problem of missing data due to withdrawal, loss to follow-up or non-completion of questionnaire data.

Decision Points

There will be no formal interim analysis. The results once analysed will be reviewed by the research team, the Trial Steering Committee (TSC) and Data and Safety Monitoring Committee (DSMC) and the PI/CI and other collaborators.

The Level of Statistical Significance

Level of significance will be tested as a 5% two-sided significant level.

Procedure for Accounting for Missing, Unused, and Spurious Data.

Missing data: Missing data will be reported with reasons given where available and the missing data pattern will be examined. We will explore the mechanism of missing data, though the mixed effects model implicitly accounts for data missing at random. The need for a sensitivity analysis taking into account missing data using multiple imputation will be considered and outlined further in the SAP. Spurious data will be assessed using standard editing criteria.

Procedures for Reporting any Deviation(s) from the Original Statistical Analysis Plan (SAP)

The final statistical plan will be agreed prior to final data lock and prior to any analyses taking place. Any deviation thereafter will be reported in the final trial report.

TRIAL OVERSIGHT

The trial will be conducted in accordance with the current approved protocol, GCP, relevant regulations and standard operating procedures. A risk assessment and monitoring plan are not being prepared before the study opens, as it is a low risk intervention.

Trial committees

For peer review only

A trial steering committee (TSC) will convene prior to the study starting and half-yearly thereafter to review and address key aspects of the study including the following:

1. Recruitment
2. Safety/adverse event
3. Withdrawals
4. Data management
5. Statistical analysis plan

The TSC will also function as a data safety and monitoring committee (DSMC) for this particular study and there will be a smaller trial management committee as outlined in the study synopsis, which will focus more on the week-to-week running of the trial and will be on a more regular basis.

Monitoring

Direct access will be granted to authorised representatives from the Sponsor within the appropriate department and host institution for monitoring and/or audit of the study to ensure compliance with regulations. Following written standard operating procedures, the monitoring visits will verify that the clinical trial is conducted and data are generated, documented and reported in compliance with the protocol, GCP and the applicable regulatory requirements.

SAFETY REPORTING

Adverse Event Definitions

Serious Adverse Event (SAE)	A serious adverse event is any untoward medical occurrence that:<br>• results in death<br>• is life-threatening<br>• requires inpatient hospitalisation or prolongation of existing hospitalisation<br>• results in persistent or significant disability/incapacity<br>• consists of a congenital anomaly or birth defect.<br>Other 'important medical events' may also be considered a serious adverse event when, based upon appropriate medical judgement, the event may jeopardise the participant and may require medical or surgical intervention to prevent one of the outcomes listed above.<br>NOTE the term "life-threatening" in the definition of "serious" refers to an event in which the participant was at risk of death at the time of the event; it does not refer to an event, which hypothetically might have caused death if it were more severe.

NB: to avoid confusion or misunderstanding of the difference between the terms "serious" and "severe", the following note of clarification is provided: "Severe" is often used to describe intensity of a specific event, which may be of relatively minor medical significance. "Seriousness" is the regulatory definition supplied above.

For peer review only

Procedures for Reporting Adverse Events

A serious adverse event (SAE) occurring to a participant will be reported to the REC that gave a favourable opinion of the study where in the opinion of the Chief Investigator the event was 'related' (resulted from administration of any of the research procedures) and 'unexpected' in relation to those procedures. Reports of related and unexpected SAEs should be submitted within 15 working days of the Chief Investigator becoming aware of the event, using the HRA report of serious adverse event form (see HRA website). The severity of events will be assessed on the following scale: 1 = mild, 2 = moderate, 3 = severe.

Non-serious AEs considered related to the trial intervention as judged by a medically qualified investigator or the Sponsor will be followed up once the event is considered stable. It will be left to the Investigator's clinical judgment to decide whether or not an AE is of sufficient severity to require the participant's removal from the trial. A participant may also voluntarily withdraw from the trial due to what he or she perceives as an intolerable AE. If either of these occurs, the participant must undergo an end of trial assessment and be given appropriate care under medical supervision until symptoms cease, or the condition becomes stable.

Events exempt from immediate reporting as SAEs

There are a number of expected admissions/consultations with healthcare providers that will be expected take place as part of the natural history of pre-eclampsia and gestational hypertension during the trial period. These will be classed as 'Foreseeable Events' exempt from reporting as SAEs and include:

- Severe hypertension;
- Maternal morbidity: visual disturbance; pulmonary oedema; respiratory failure; myocardial ischaemia; hepatic dysfunction, hepatic haematoma or rupture; and acute kidney injury;
- Postpartum haemorrhage;
- Lower genital tract bleeding;
- Sepsis;
- Admission to hospital for pre-eclampsia, monitoring of hypertension, or symptoms of low blood pressure
- Pre-planned hospitalisation;
- Diagnostic and therapeutic procedures including blood transfusion;
- Worsening pruritis;
- A pre-existing maternal condition (such as renal disease), unless it causes increased clinical concern;
- Admission for psychiatric or social reasons;
- Retained placenta;
- Extended hospital stay of the mother due to the need to keep the baby/babies in hospital;
- Neonatal care unit admission for indications unrelated to pregnancy hypertension, such as neonatal hyperbilirubinaemia or unanticipated care for a fetal anomaly; or
- Fetal congenital anomaly

This list is not exhaustive and therefore any other 'minor medical significance symptom', as judged by the CI/PI, which does not require inpatient hospitalisation/prolongation of existing hospitalisation or result in persistent or significant disability/incapacity, and is not life-threatening and, does not result in death, will not be classed as an adverse event not an SAE.

DISCUSSION

Until recently, key evidence missing from trials of self-monitoring/tele-monitoring was whether it actually led to lower BP. In 2018, the TASMIN-H4 randomised trial [38] showed that GPs using self-monitored BP to titrate anti-hypertensives, with or without tele-monitoring, achieved better BP control for patients using tele-monitoring. As with previous trials, the mechanism of action appeared to be medication optimisation. More recent work shows that patient and clinician experience was largely positive and cost-effectiveness analysis suggests that self-monitoring in this context is cost-effective by NICE criteria [39]. Self-monitoring can be combined with self-titration of medication, a process known as self-management. The SNAP-HT trial [1] demonstrated that self-management postpartum following a hypertensive pregnancy offers great promise. The purpose of the POP-HT trial is to assess whether this BP reduction can be reproduced in a larger, randomised, single-blinded study powered to detect differences in BP as the primary outcome.

Our group have studied women 10 years after hypertensive and normotensive pregnancies to characterise their cardiovascular, vascular and cerebro-vascular systems. Consistent with previous reports [16, 17, 40], women who had been through hypertensive pregnancy were more likely to have LVH, increased LV mass and impaired diastolic function. In addition, cerebrovascular changes were evident including; lower grey matter volumes, and greater white matter lesion density compared to the control population [12, 41]. Vascular phenotypic changes included reduced capillary density and increased aortic stiffness [13, 42]. These phenotypic differences were not explained by differences in traditional cardiovascular risk factors at the time of assessment. It is possible such differences emerge during pregnancy and persist independent of postpartum BP variability but this is not really known[43] and, although the cardiovascular adaptations that emerge during hypertensive pregnancies reverse to some extent postpartum [15-18], an alternative hypothesis is that long term 'risk' reflects a failure of the cardiac, vascular or cerebral systems to 'normalise' after pregnancy [19, 20, 43, 44]. In POP-HT, the trial aims to determine if these phenotypic changes emerge as early as 6-9 months and if so, whether they can be mitigated by postpartum blood pressure optimization. If so, this may offer one approach to modify long-term end organ changes, in addition to any beneficial effects on blood pressure that are demonstrated in this trial.

ETHICS AND DISSEMINATION

Declaration of Helsinki

The Investigator will ensure that this trial is conducted in accordance with the principles of the Declaration of Helsinki.

Guidelines for Good Clinical Practice

For peer review only

The Investigator will ensure that this trial is conducted in accordance with relevant regulations and with Good Clinical Practice. The trial protocol and all accompanying documentation has been approved by the Sponsor, an external REC, the HRA (Ethics Ref: 19/LO/1901; IRAS Project ID: 273353) and OUH (the local NHS trust) trial management authority (TMA).

Consent

The participant must personally sign and date the latest approved version of the Informed Consent form(s) before any trial specific procedures are performed. Please see appendix E for details of how consent will be modified during the COVID-19 pandemic to reduce risk of the paper acting as a vector for transmission of Coronavirus. Full details on the consent process are in the fully study protocol.

Participant Confidentiality

The study will comply with the General Data Protection Regulation (GDPR) and Data Protection Act 2018, which require data to be de-identified as soon as it is practical to do so. Further details are outlined in the study protocol and participant information sheets.

Access to Data

Direct access will be granted to authorised representatives from the Sponsor, host institution and the regulatory authorities to permit trial-related monitoring, audits and inspections. Further detail is provided in the fully study protocol.

Data Management

The study will comply with the General Data Protection Regulation (GDPR) and Data Protection Act 2018. The University of Oxford, as sponsor will act as data controller for the study.

FUNDING

The research is being financed by a British Heart Foundation Clinical Research Training Fellowship (BHF Grant number FS/19/7/34148).

INSURANCE

The University has a specialist insurance policy in place, which would operate in the event of any participant suffering harm because of their involvement in the research (Newline Underwriting Management Ltd, at Lloyd's of London). NHS indemnity operates in respect of the clinical treatment that is provided.

PUBLICATION POLICY

The Investigators will be involved in reviewing drafts of the manuscripts, abstracts, press releases and any other publications arising from the study. Authors will acknowledge that the study was funded by the British Heart Foundation Clinical Research Training Fellowship (BHF Grant number

For peer review only

FS/19/7/34148). Authorship will be determined in accordance with the ICMJE guidelines and other contributors will be acknowledged. The summarised results will be published in a scientific journal/s and summarised on the CCRF website for participants to read. Should participants wish to have a copy of any papers published, they merely need to contact the study team, using the contact details provided on their PIS, and the team would be happy to provide one.

AUTHORS'S CONTRIBUTIONS

JK, AJL, RM, KT, YK, CA, AF, AC. LM and PL contributed to the design of the study, JK, AJL and RM and PL secured funding. JK, AJL, RM, YK, AMC, AF, WW, KS, LM, CA, AC, MS, CR, KT, WL and PL refined the overall study protocol and lead the project delivery. BT and LC have provided guidance and external refinement. JK, MS and CR designed and oversee the delivery of the intervention. JK, AMC, YK, and WW will contribute to 24hr BP data acquisition and analysis. AJL, WL, JK and RM contributed to the development of the Brain and Cardiac MRI protocols. JK, RM, MK, WL and AJL will contribute to MRI image acquisition and quality control. WL will lead brain MRI image processing and analysis. JK will lead the cardiac MRI analysis with support from AJL, MK and WW. BR and LT will lead renal MRI analysis. Echocardiography acquisition and analysis will be overseen by JK and performed by blinded study investigators. Cardiopulmonary exercise testing and peripheral cardiovascular risk assessment will be overseen by JK, AJL, WW, AMC and PL. HH will oversee retinal image acquisition and analysis. AF will run the sub-study on endothelial cells and, CT will oversee analysis of circulating biomarkers within the sub-study alongside AF.

COMPETING INTERESTS

LM is supported by the NIHR Oxford Biomedical Research Centre and is a part-time employee of Sensyne Health PLC and holds shares in this company. RJM has received BP monitors for research from Omron and is working with them to develop a telemonitoring system. Any fees / consultancy from this work are paid to his institution.

APPENDICES/SUPPLEMENTARY MATERIAL

See supplementary file

For peer review only

REFERENCES

1. Ananth, C.V., K.M. Keyes, and R.J. Wapner, *Pre-eclampsia rates in the United States, 1980-2010: age-period-cohort analysis.* BMJ, 2013. **347**:p.f6564.
2. Walters, B.N. and T. Walters, *Hypertension in the puerperium.* Lancet, 1987. **2**(8554):p.330.
3. McDonald, S.D., et al., *Cardiovascular sequelae of preeclampsia/eclampsia: a systematic review and meta-analyses.* Am Heart J, 2008. **156**(5):p.918-30.
4. Ray, J.G., et al., *Cardiovascular health after maternal placental syndromes (CHAMPS): population-based retrospective cohort study.* Lancet, 2005. **366**(9499):p.1797-803.
5. Behrens, I., et al., *Risk of post-pregnancy hypertension in women with a history of hypertensive disorders of pregnancy: nationwide cohort study.* BMJ, 2017. **358**:p.j3078.
6. Lazdam, M., et al., *PP032. Unique features of long-term cardiovascular phenotype in young women with early-onset pre-eclampsia.* Pregnancy Hypertens, 2012. **2**(3):p.259-60.
7. Cairns, A.E., et al., *Self-Management of Postnatal Hypertension: The SNAP-HT Trial.* Hypertension (Dallas, Tex, 2018:**1979.72**(2):p.425-432.
8. Law, M.R., J.K. Morris, and N.J. Wald, *Use of blood pressure lowering drugs in the prevention of cardiovascular disease: meta-analysis of 147 randomised trials in the context of expectations from prospective epidemiological studies.* BMJ, 2009.**338**:p.b1665.
9. National Institute for Clinical Excellence (N.I.C.E), *Hypertension in Prengnacy: diagnosis and management*, NICE, Editor. 2019:https://www.nice.org.uk/guidance/ng133.
10. Webb, A.J., et al., *Response of day-to-day home blood pressure variability by antihypertensive drug class after transient ischemic attack or nondisabling stroke.* Stroke, 2014.**45**(10):p.2967-73.
11. Webb, A.J.S., et al., *Prognostic Significance of Blood Pressure Variability on Beat-to-Beat Monitoring After Transient Ischemic Attack and Stroke.* Stroke, 2018. **49**(1):p.62-67.
12. Siepmann, T., et al., *Long-term cerebral white and gray matter changes after preeclampsia.* Neurology, 2017. **88**(13):p.1256-1264.
13. Boardman, H., et al., *Comprehensive multi-modality assessment of regional and global arterial structure and function in adults born preterm.* Hypertens Res, 2016. **39**(1):p.39-45.
14. Melchiorre, K., et al., *Maternal cardiac dysfunction and remodeling in women with preeclampsia at term.* Hypertension, 2011. **57**(1):p.85-93.
15. Melchiorre, K. and B. Thilaganathan, *Maternal cardiac function in preeclampsia.* Curr Opin Obstet Gynecol, 2011. **23**(6):p.440-7.
16. Ghossein-Doha, C., et al., *Age-related alterations in cardiac geometry in formerly preeclamptic women and healthy parous controls: an explorative study.* Reprod Sci, 2013. **20**(1):p.39-44.
17. Ghossein-Doha, C., et al., *Hypertension after preeclampsia is preceded by changes in cardiac structure and function.* Hypertension, 2013. **62**(2):p.382-90.
18. Ghossein-Doha, C., et al., *Maternal cardiac adaptation to subsequent pregnancy in formerly pre-eclamptic women according to recurrence of pre-eclampsia.* Ultrasound Obstet Gynecol, 2016. **47**(1):p.96-103.
19. Lazdam, M., et al., *Prevention of vascular dysfunction after preeclampsia: a potential long-term outcome measure and an emerging goal for treatment.* J Pregnancy, 2012. **2012**:p.704146.
20. Lazdam, M., et al., *Elevated blood pressure in offspring born premature to hypertensive pregnancy: is endothelial dysfunction the underlying vascular mechanism?* Hypertension, 2010. **56**(1):p.159-65.
21. Rai, T., et al., *Optimizing a digital intervention for managing blood pressure in stroke patients using a diverse sample: Integrating the person-based approach and patient and public involvement.* Health Expect, 2020.
22. Yu, G.Z., et al., *Association of Maternal Antiangiogenic Profile at Birth With Early Postnatal Loss of Microvascular Density in Offspring of Hypertensive Pregnancies.* Hypertension, 2016. **68**(3):p. 749-759.

For peer review only

23. Lewandowski, A.J., et al., *Elevated Blood Pressure in Preterm-Born Offspring Associates With a Distinct Antiangiogenic State and Microvascular Abnormalities in Adult Life.* Hypertension, 2015. **65**(3):p.607-614.
24. Lewandowski, A.J., et al., *Right ventricular systolic dysfunction in young adults born preterm.* Circulation, 2013. **128**(7):p.713-720.
25. Lewandowski, A.J., et al., *Preterm heart in adult life: cardiovascular magnetic resonance reveals distinct differences in left ventricular mass, geometry, and function.* Circulation, 2013. **127**(2):p. 197-206.
26. Davis, E.F., et al., *Pre-eclampsia and offspring cardiovascular health: mechanistic insights from experimental studies.* Clinical science, 2012. **123**(2):p.53-72.
27. Aye, C.Y.L., et al., *Disproportionate cardiac hypertrophy during early postnatal development in infants born preterm.* Pediatr Res, 2017. **82**(1):p.36-46.
28. Turbeville, H.R. and J.M. Sasser, *Preeclampsia beyond pregnancy: Long-term consequences for mother and child.* American Journal of Physiology-Renal Physiology, 2020. **318**(6):p.F1315-F1326.
29. Agatisa, P.K., et al., *Impairment of endothelial function in women with a history of preeclampsia: an indicator of cardiovascular risk.* American Journal of Physiology-Heart and Circulatory Physiology, 2004. **286**(4):p.H1389-H1393.
30. Kvehaugen, A.S., et al., *Endothelial function and circulating biomarkers are disturbed in women and children after preeclampsia.* Hypertension, 2011. **58**(1):p.63-69.
31. Sandvik, M.K., et al., *Preeclampsia in healthy women and endothelial dysfunction 10 years later.* American journal of obstetrics and gynecology, 2013. **209**(6): p.569.e1-569.e10.
32. Munoz-Hernandez, R., et al., *Decreased level of cord blood circulating endothelial colony-forming cells in preeclampsia.* Hypertension, 2014. **64**(1):p.165-71.
33. Reinisch, A., et al., *Humanized large-scale expanded endothelial colony–forming cells function in vitro and in vivo.* Blood, The Journal of the American Society of Hematology, 2009. **113**(26):p.6716-6725.
34. Yoder, M.C., *Is endothelium the origin of endothelial progenitor cells?* Arterioscler Thromb Vasc Biol, 2010. **30**(6): p. 1094-103.
35. Topouchian, J., et al., *Clinical accuracy of the Omron M3 Comfort((R)) and the Omron Evolv((R)) for self-blood pressure measurements in pregnancy and pre-eclampsia - validation according to the Universal Standard Protocol.* Vasc Health Risk Manag, 2018. **14**:p.189-197.
36. Melchiorre, K., et al., *Preeclampsia is associated with persistent postpartum cardiovascular impairment.* Hypertension, 2011. **58**(4):p.709-15.
37. Huckstep, O.J., et al., *Physiological Stress Elicits Impaired Left Ventricular Function in Preterm-Born Adults.* J Am Coll Cardiol, 2018. **71**(12):p.1347-1356.
38. McManus, R.J., et al., *Efficacy of self-monitored blood pressure, with or without telemonitoring, for titration of antihypertensive medication (TASMINH4): an unmasked randomised controlled trial.* Lancet, 2018. **391**(10124):p.949-959.
39. Grant, S., et al., *Using mHealth for the management of hypertension in UK primary care: an embedded qualitative study of the TASMINH4 randomised controlled trial.* Br J Gen Pract, 2019. **69**(686):p.e612-e620.
40. Strobl, I., et al., *Left ventricular function many years after recovery from pre-eclampsia.* BJOG, 2011. **118**(1):p.76-83.
41. Boardman, H., et al., *Variations in Cardiovascular Structure, Function, and Geometry in Midlife Associated With a History of Hypertensive Pregnancy.* Hypertension, 2020. **75**(6):p.1542-1550.
42. Boardman, H., et al., *Aortic stiffness and blood pressure variability in young people: a multimodality investigation of central and peripheral vasculature.* J Hypertens, 2017. **35**(3):p.513-522.
43. Kalafat, E. and B. Thilaganathan, *Cardiovascular origins of preeclampsia.* Curr Opin Obstet Gynecol, 2017. **29**(6):p.383-389.

For peer review only

44. Thilaganathan, B. and E. Kalafat, *Cardiovascular System in Preeclampsia and Beyond.* Hypertension, 2019. **73**(3):p.522-531.

For peer review only

FIGURE LEGENDS

Figure 1: Illustration of self-management of blood pressure for women randomised to the intervention

Figure 2: Flow Chart of Proposed Study visits

For peer review only

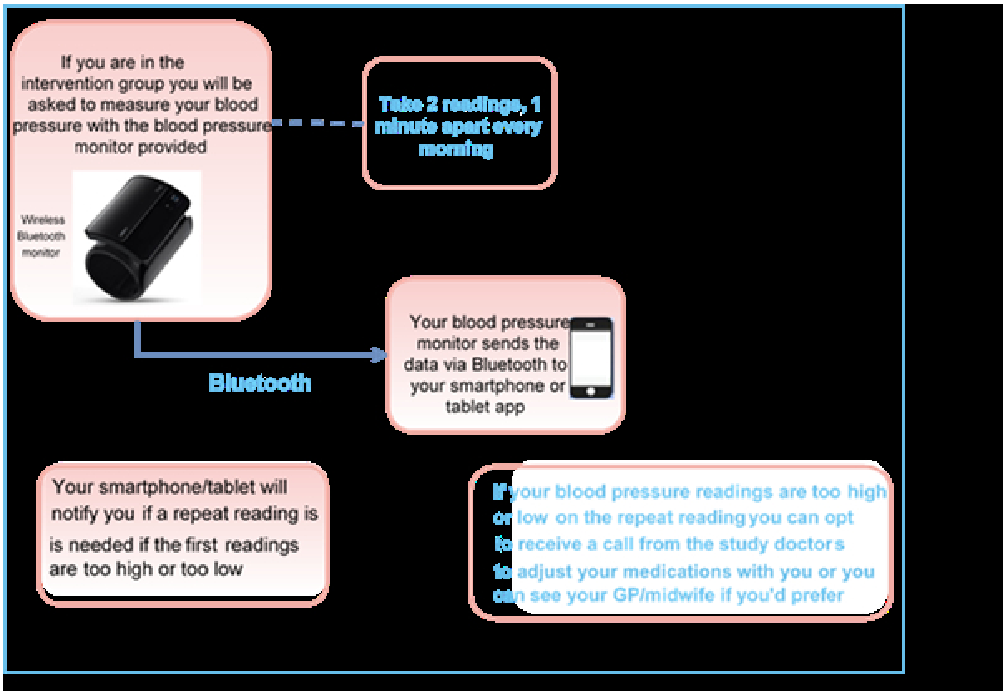

Figure 1: Illustration of self-management of blood pressure for women randomised to the intervention

63x43mm (240 x 240 DPI)

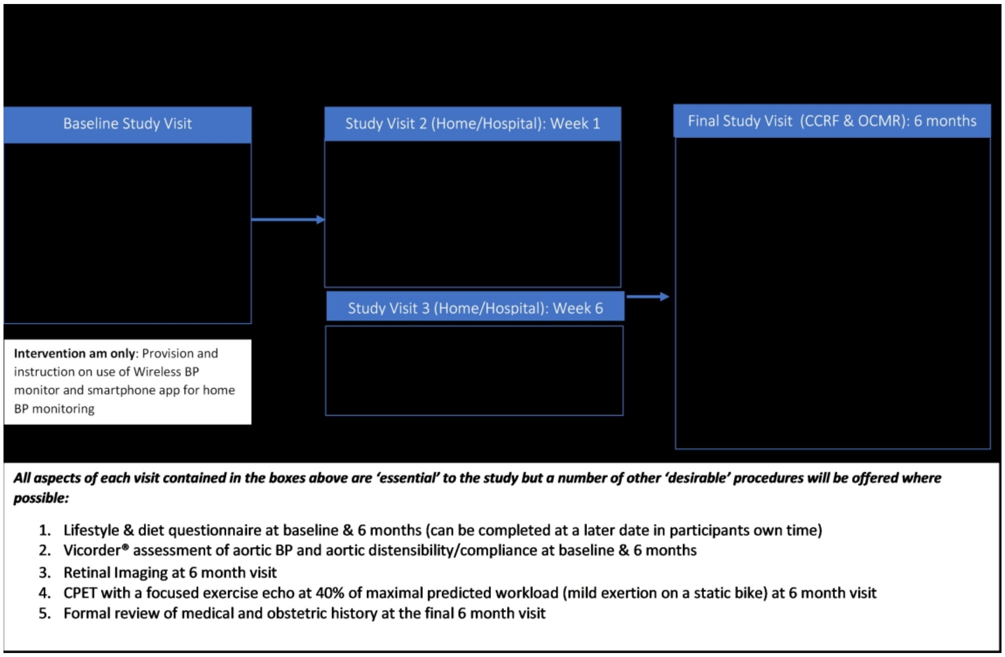

Figure 2: Flow Chart of Proposed Study visits

317x205mm (240 x 240 DPI)

APPENDIX A: TRIAL FLOW CHART

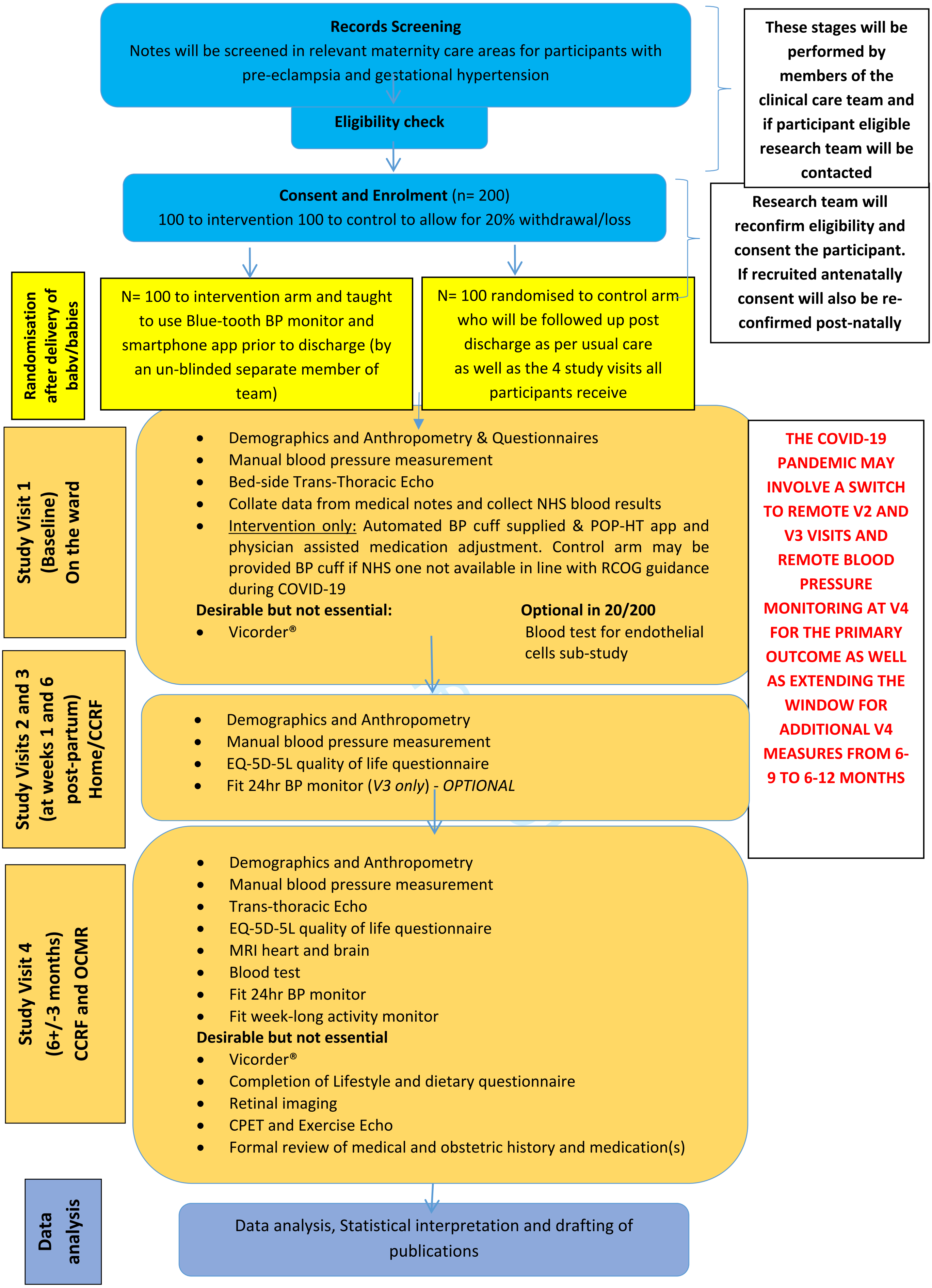

APPENDIX B: SCHEDULE OF PROCEDURES (MAIN STUDY)

**Procedures**	**Visit 0: Consent**		**Visit 1: Baseline**		**Visit 2**		**Visit 3**		**Visit 4**	
	Intervention arm	Control arm	Intervention arm	Control arm	Intervention arm	Control arm	Intervention arm	Control arm	Intervention arm	Control arm
Eligibility assessment	X	X								
Informed consent	X	X								
BP measurement			X	X	X	X	X	X	X	X
Demographics & anthropometry			X	X	X	X	X	X	X	X
Echocardiogram			X	X					X	X
Data collection: medical notes and NHS blood results			X	X						
Lifestyle & Diet questionnaire			X	X					X	X
Vicorder ®(vascular assessment)			X	X					X	X

For peer review only

Intervention: Automated BP cuff provision and Smartphone app installation			X	During pandemic cuff may be provided						
Home BP self-monitoring and physician assisted medication adjustment post hospital discharge					X		X		X	
EQ-5D-5L questionnaire					X	X	X	X	X	X
Fitting 24hr blood pressure monitor							X	X	X	X
MRI of heart and brain									X	X
Blood test			X (if in cells sub-study)	X (if in cells sub-study)					X	X
Fit accelerometer									X	X
Retinal imaging									X	X
Review of medical and									X	X

For peer review only

For peer review only - http://bmjopen.bmj.com/site/about/guidelines.xhtml

obstetric history										
CPET with exercise echo at 40% workload									X	X

For peer review only

APPENDIX C: PROCEDURES FOR ENDOTHELIAL CELLS SUB-STUDY

**Procedures**	**Visit 1: Baseline**		**Visit 2**	
**Eligibility assessment**	X	X		
**Informed consent**	X	X		
**BP measurement**	X	X	X	X
**Demographics & anthropometry**	X	X	X	X
**Blood test**	X	X	X	X

For peer review only

APPENDIX D: POP-HT Tele-monitoring system software and network architecture

The software and network architecture of the POP-HT INTERVENTION can be summarised as follows (from left to right in Figure D.1 below): the participants first take BP readings using a Bluetooth-low-energy (BLE) enabled Blood Pressure device (OMRON Evolv®). The data are transmitted to their POP-HT App, available on both Android and iOS mobile devices, via BLE. The App communicates with the POP-HT web-application, available from a public domain on the internet, over encrypted HTTPS, using Representational state transfer (REST) Application Programming Interfaces (APIs), secured by the JSON Web Token protocol. The web-application runs on a web-server hosted in an authorised and secured virtual machine from Oxford University Hospitals (OUH). It includes rule-based algorithms (based on the on/off treatment BP tables below) that process the participants' BP readings and output: (i) the BP level, (ii) the participants' next action, and (iii) the suggested frequency of BP readings. The web-application uses an App notification service (Firebase, Google, USA, https://firebase.google.com) to send messages to the participants' Apps, such as missing data warnings and medication changes. An SMS gateway service (Esendex, UK, https://www.esendex.co.uk) is also used, as a backup, to send messages in case the notification service becomes temporarily unavailable. The SMS system is also responsible for sending and recovering login credentials. E-mails are sent twice daily to clinical researchers, using NHS SMPT servers, summarising the BP and medication status, including triggers to down-titrate, of all study participants in the POP-HT system. The web-application allows participants to review all of their data while e.g., only the last 2 weeks or messages and BP are displayed on the App. Finally, the web-application also allows clinical researchers to register new participants, create and manage their treatment plan, review the participants dashboard, resolve abnormal BP and missing data flags, and export pseudo-anonymised data (identified only by the study IDs) to carry out statistical analysis.

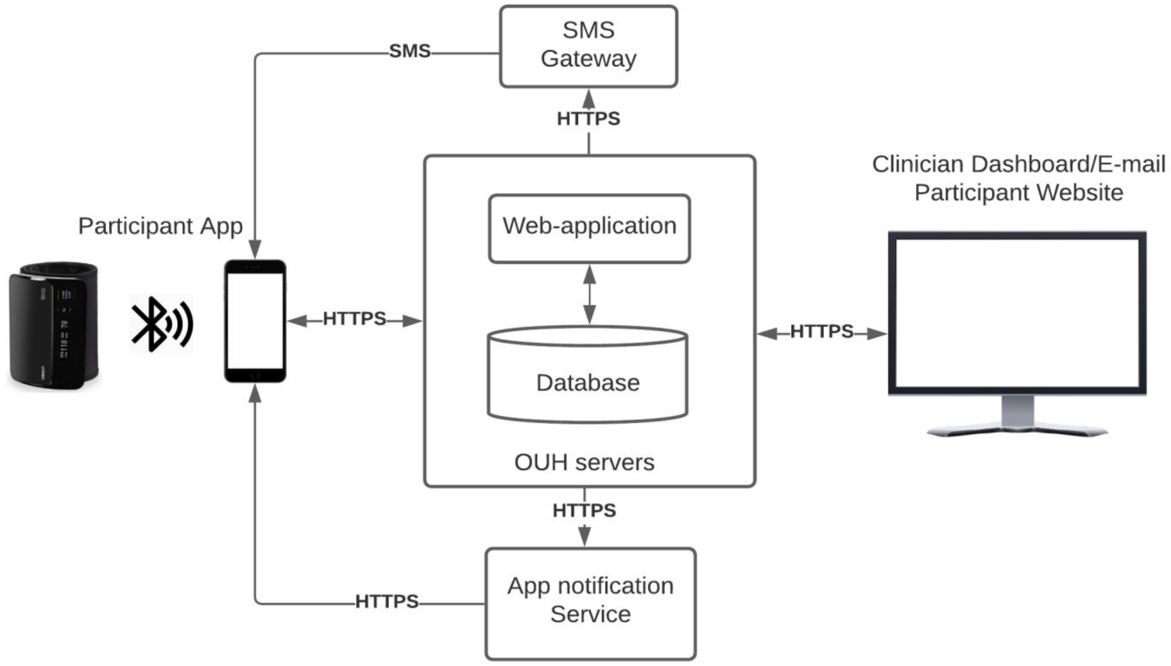

Figure D.1: Illustration of the POP-HT tele-monitoring system software and network architecture

Process of Self-Management

The decisions outlined below in Figure D.2 are based on the participant taking two consecutive BP readings (2 minutes apart), and the automated algorithm decision being made on the second reading. On this note, if more than 2 readings are taken by the participant, all readings are synchronised from the monitor and submitted to the OUH-NHS server, but the decision is made only on the last reading.

Figure D.2 Traffic light table of BP ranges, BP classification and pre-programmed actions whilst ON treatment

Colour	Level	BP	Action
Red	Very high	Sys 160 or more<br>OR<br>Dia 110 or more<br>OR<br>Symptoms	Repeat BP in 5 minutes.<br>If this is a repeat reading: contact local maternity unit immediately for urgent assessment today.
Orange	High	Sys 150-159<br>OR<br>Dia 100-109	Repeat BP in 5 minutes.<br>If this is a repeat reading: Call from study physician 9-5pm AND to see own GP/midwife for an URGENT (same-day) appointment.* Switch to twice daily readings until back in yellow/green
Yellow	Raised	Sys 140-149<br>OR<br>Dia 90-99	No action.
Green	High normal	Sys 130-139<br>OR<br>Dia 80-89	No action
Blue	Low normal	Sys 100-129<br>AND<br>Dia < 80	Switch to twice daily readings and if in this zone for 2 consecutive days, medication titration will be signed off by study doctor and instructions sent via app to participant
Purple	Low	Sys < 100<br>AND<br>Dia < 80	Repeat BP in 5 minutes.<br>If this is a repeat reading: option of opting for a call from study physician 9-5pm vs. opting to see own GP/midwife for an URGENT appointment*. Switch to twice daily readings until back in yellow/green

*During COVID-19, NHS clinicians may not see the patient in person the same day but a tele-medicine review at the minimum will be advised.

If the participant has no 3G/4G signal or Wifi when they synchronise the OMRON readings to the phone, and/or there is an error in the transmission of the readings to the OUH-NHS server, the readings are deemed as 'valid' by the App for 4 hours. After 4 hours, if the participant enters the 'Enter my readings' section and tries to re-sync with the server, they will be asked to take new readings with the OMRON. Once the synchronisation with the server is re-established, all readings, regardless of their timestamp will be sent to the server. The SMS system is also a backup to allow the participants to notify the study doctors of their readings. The messages used are essentially the same, small changes only being required to map the same App functionality to the more "limited" SMS system.

The frequency of readings requested of the participant via the app will be adjusted according to whether the readings are high, low normal or low. If they are in the orange, blue or purple zones above (high, low normal or low), they will be asked by the app to submit readings twice daily.

If the reading is abnormal, as outlined in the table they will be asked to 'Repeat the BP in 5 minutes'.

If the repeat reading submitted is in the red zone i.e.>160/110mmHg they will be sent a notification asking them to contact their local maternity unit immediately for urgent assessment (see 'Safety Netting' section below for more detail).

If the repeat reading is in the orange or purple BP ranges above, participants will be notified via an app notification and will have the option of:

- Either selecting via the app to be called by a 'physician' (who will be a clinician within the research team) to discuss and make medication adjustment over the telephone,
- Or seeing their GP/Midwife/maternity unit on an urgent basis to have their medications adjusted, which they will then update on the app. During COVID-19 given the difficulties in seeing their own GP/mid-wives, it is expected more women will want remote physician support.

The software will send a notification to the participant using more 'participant friendly' wording as agreed in several rounds of PPI and as tested on real-life volunteers as part of the PPI process: For example, if the blood pressure reading submitted is 152/100mmHg it will trigger this notification:

'*Your blood pressure reading is a little higher than we would ideally like it and the repeat reading was also a little high. Select **Yes** to be called by a specialist doctor to have your medication adjusted or **No** to see your own GP/midwife/maternity unit urgently?*

They then have the option of selecting '**YES**' or '**No**' on the app which triggers the following actions to be taken

- **Yes**: The study team will call them the same working day (if out of hours, at the beginning of the next working day) to review their symptoms and to adjust their medications. If the doctor calling has any concern or if the participant feels unwell prior to the call then they will be aware of the need to see a health professional from the information sheet, the app and/or the call, as appropriate. During the call, they will be advised of the need to have blood tests checked if this reading is in the 1st 2 weeks' post-partum in the orange zone. This should be done over the next 24 hours by the GP/local maternity unit. They will also be asked to monitor twice daily until 2 sets of readings are back in the normal (yellow/green zones). The medication schedule will be updated on the web-platform after the call and synchronise to the app so it will be written down for the participant too. The next day the participant will be asked to confirm their new medication schedule on the app with a YES/NO as a safety check. If it is NO they will be asked to enter, what they are taking and any discrepancy will be clarified with the research team over the phone.

- **No**: The app will notify them that urgent midwife/GP assessment is advised and the team will call the same day to check this is in progress and enquire about any medication adjustment. They will also be asked to monitor twice daily until 2 sets of readings are back in the normal (yellow/green zones).

For peer review only

'Safety netting' built into the system

The app has been tested for in detailed simulation scenarios e.g. high BP, low BP, fluctuant BP, non-compliers, anxious and for all eventualities based on our experience from SNAP-HT [7] and it has proved robust in these 'test scenarios'. Automatic e-mail alerts will be triggered to the clinical members of the study team and flags will generate on the website next to that participant's study ID, for those BP readings 'out of target range', and/or if readings are consistently not being recorded and/or uploaded via the app by a particular participant. These secure e-mails will be sent (as explained further in appendix E) so that appropriate action can be taken in a timely fashion to adjust medications for those readings out of range as explained above. For those readings in the red zone, a phone call will also be made that same working day (9-5pm) to ensure arrangements have been made for assessment by an NHS provider, and in the event they cannot be contacted, their GP/midwife will be notified.

Once participants have switched to once weekly readings i.e. they have been off-treatment for 5 days with readings in the normal range, and have been notified by the app to make this switch, motivational reminders will be sent on a weekly basis. If the readings go up or down outside of the normal range whilst doing once weekly readings i.e. if they are not in the green or blue zones, they will be asked to repeat the reading and if the repeat reading is also outside of these zones, the relevant actions will be triggered as outlined below in figure D.3

Figure D.3 Traffic light table of BP ranges classification and pre-programmed actions whilst OFF treatment

Colour	Level	BP	Action
Red	Very high	Sys 160 or more<br>OR<br>Dia 110 or more	Repeat BP in 5 minutes.<br>If this is a repeat reading*: contact your local maternity unit immediately for urgent assessment today. Switch to once daily readings
Orange	High	Sys 140-159<br>OR<br>Dia 90-109	Repeat BP in 5 minutes.<br>If this is a repeat reading and the value is in this range for 2 or more days in a row option of opting for a call from study doctors between 9-5pm vs. opting to see own GP/midwife in next 48hrs. Switch to once daily readings until further notification
Green	Normal	Sys < 140<br>OR<br>Dia < 90	No action. Continue weekly readings
Blue	Low normal	Sys 100-129<br>AND<br>Dia < 80	No action unless symptoms (report via the app/website) e.g. light-headed/dizzy/faint in which case please notify study team who will call you within 48 hours

Purple	Low	Sys < 100<br>AND<br>Dia < 80	Repeat BP in 5 minutes.<br>If this is a repeat reading and the value is in this range for 2 or more days in a row option of opting for a call from study doctors between 9-5pm vs. opting to see own GP/midwife in next 48hrs. Switch to once daily readings until further notification

*If the repeat reading is 'Red' a flag/notification will also be sent to the study team to call the participant to check they have followed the action just as for the on-treatment algorithm. For orange and purple zones, the research team will also be notified and the participants will be asked to switch back to daily readings. If the readings remain outside of the green/blue zones for >2 days the app will offer the option of being contacted shortly by the study team or opting to see their own GP/midwife, as per the pathways for the on-treatment algorithm. It is unlikely that women will need to-restart treatment based on the pilot data from SNAP-HT [4], where only 1/91 women needed to re-start medication after stopping.

In the case of failure to submit readings, automatic 'motivating' notifications will be sent at 24hours to the participants, and notifications are also sent to the study team after 36 hours without a reading during the first 2 weeks following discharge. This is to prompt a call to the participant to discuss any problems. If the participant repeatedly fails to submit a reading for >36 hours during the first 2 weeks, then they may be withdrawn from the study at the discretion of the PI/CI. Their GP will also be called and mailed; and the participant will be called, messaged and e-mailed advising urgent medical review.
Similar 'safety alerts' will be sent out if a participant records any side-effects/SAEs (which can be accessed and reviewed by the study team via a secure log-on portal). If there is anything deemed to require further action the PI/CI, GP and participant will be notified urgently.

APPENDIX E: MODIFICATIONS TO STUDY DESIGN TO MITIGATE THE IMPACT OF COVID-19

On March 17th 2020, the COVID-19 pandemic meant that all research across Oxford University Hospitals (OUH) NHS Foundation Trust had to be halted. A minor amendment to

For peer review only

perform remote V2 and V3 visits allowed us to continue the follow up of 18/200 women already recruited as of 06/05/2020. A further two amendments allowed the POP-HT RCT to restart safely during the COVID-19 pandemic as the study received a Stage 3 exception from OUH NHS Foundation Trust, due to its contribution to the clinical care of these women. Recruitment to the trial recommenced in early June 2020.

The following changes are explained in more detail below to explain how the study will run safely and in line with local COVID-19 guidance:

Remote study visits were established early to allow entirely remote follow up visits for week 1 and week 6, equivalent to visits 2 and 3 (non-substantial amendment 2.0 25/03/20201). The 1st 18 participants' recruited pre-COVID had been followed up remotely successfully, demonstrating the technique to be both effective and feasible for the remaining 182/200. None of the 1st 18 participants required face-face contact for review of their medications and obstetric history, or for use the POP-HT app and the solving of any technical issues for those in the intervention arm. Remote blood pressure measurement (both clinic and ambulatory 24hr blood pressure monitoring) was achieved successfully for all of the 1st 18 participants recruited pre-COVID 19 using ZOOM® or MS TEAMS®. During this period of follow up, no new baseline visits were performed during the 1st wave of the COVID-19 pandemic.

When the study restarted recruitment in June 2020 the following amendments were made to the baseline visit with re-design of the recruitment, consent and enrolment process to minimise direct patient contact and risk of virus transmission:

a. Provision of documents: PIS, flyer and additional information sheet provided by the clinical team to the participant on a Tablet/Ipad® (sterilised with CLINELL® wipes). The participants can then review them in this format (and a copy e-mailed to them for their records once consent has been obtained and they are enrolled).
b. Consent: Consent forms will be placed in wipe down wallets, which will be handed to the participant for signing wearing sterile gloves. Once signed the form will be photocopied whilst wearing gloves. The copy will then be placed back into a sterile wallet for the participant and the original will be placed in a second wallet in their notes. Both will be wiped down with CLINELL® wipes and the research teams' copy will be kept securely in a wipe-down file/ring-binder in 'quarantine' before moving them to CCRF after 48hours.
c. The Vicorder® was made an optional measurement to reduce the amount of time in direct patient contact
d. The Echo and Vicorder® will be performed by a single investigator at the bedside. PPE will be worn (the level of which will be in line with hospital policy). Adequate training in donning and doffing of PPE has been undertaken by the study team via OXSTAR®. This will not affect OUH trust protocols for female chaperones, which can still be provided if needed by existing clinical staff on the ward.

The V2 and V3, at weeks 1 and 6 respectively, will be continued remotely for participant 19 onwards once recruitment restarted. V4 can also be done remotely for the primary outcome measures if a national lockdowns necessitate such a step to be taken.

For peer review only

APPENDIX F: AMENDMENT HISTORY

All amendments have been submitted to; and approved by SPONSOR, the REC and HRA, the local hospital (OUH) trial management authority; and other relevant parties are notified where needed.

Amendment No.	Protocol Version No.	Date issued	Author(s) of changes	Details of Changes made
01 (minor)	N/A	21/01/2020	J Kitt	Correction to wording of consent form clauses<br><br>Correction of version listed in flyer footer<br><br>IRAS form updated to list PI for OUH site
02 (minor)	V2.0	19/03/2020	P Kemp	Add option for Visit 2 (Weeks 1) and Visit 3 (Week 6) to be conducted remotely by video call.<br><br>Add the option of sending a sterile OMRON EVOLV BP monitor to participants prior to Visit 3 (Week 6).<br><br>Make the "fitting of a home blood pressure monitor" procedure optional for Visit 3 and add the option of conducting this at a later time point.
03 (minor)	V3.0	22/04/2020	J Kitt and P Kemp	Changes to visits during the COVID-19 pandemic:<br><br>Add provision of BP monitor to control arm;<br><br>Consent process to be modified to reduce risk of transmissions of COVID-19;<br><br>Extend the time point for Visit 4 to 6-12 months from 6-9;<br><br>Change the randomisation and blinding process so that only one

For peer review only

				member of staff (wearing PPE where necessary) is present at Visit 1;<br><br>Minimise participant contact during the baseline visit via use of tablets/iPads for reviewing the PIS/flyer;<br><br>Vicorder is now an optional measurement during the baseline visit.
04 (minor)	V4.0	06/10/2020	A Frost and P Kemp	Add blood test to baseline visit in both control and intervention hypertensive groups.<br><br>Include a sub population of 20 normotensive postnatal women for a new blood validation sub study.
05 (minor)	V5.0	05/01/2021	J Kitt and P Kemp	Add option for Visit 4/Final visit to be performed remotely by video call for the primary study outcome and BP based secondary outcome measures
06 (minor)	V6.0	08/03/2021	J Kitt and P Kemp	Addition of an extra sequence during the MRI scan at 6-12months to allow the kidneys to be evaluated at the same time as the heart and brain sequences are being performed. Scan duration will still remain under 60 minutes

For peer review only

APPENDIX G: COPIES OF PARTICIPANT INFORMATION SHEET (PIS) AND INFORMED CONSENT FORMS (ICF)

Oxford University Hospitals NHS Foundation Trust

Division of Cardiovascular Medicine

Radcliffe Department of Medicine &
Nuffield Department of Primary Care Health Sciences,
Oxford Heart Centre, John Radcliffe Hospital,
Oxford, OX3 9DU Tel: 01865 572833
Email: jamie.kitt@cardiov.ox.ac.uk

Physician Optimised Post-partum Hypertensive Treatment (POP-HT) Study

Participant Information Sheet (PIS): Information about a study you are invited to join

We would like to invite you to take part in a research study. Joining is entirely up to you. This information sheet explains why the research is being conducted and what it would involve if you did decide to take part. If you have any questions, please do not hesitate to ask. Please feel free to talk to others about the study.

Summary of the study

- **High blood pressure occurs in ~1 in 10 women during pregnancy** and **remains elevated in 50% even after the baby is born requiring medication at home after discharge.**
- This **study** is **investigating** whether, '**self- management' of blood pressure (BP) at home**, **after discharge**, can improve your blood pressure control and reduce the longer-term impact on your heart and brain.
- **50%** of participants (**the self-management group**) will be asked to **measure their blood pressure at home** using a **Wireless monitor** and **upload the readings via** a **smart-phone/tablet app.** If readings are abnormal then a specialist doctor can guide your medication adjustment accordingly (you will still have option of seeing your own GP/midwife if you prefer).
- **50%** (**the control group**) **will be looked after by their GP/ midwife/other NHS services as normal**. The group you are in will be decided by random. During the COVID-19 pandemic a BP monitor will also be provided to the control arm to allow home readings to be measured as per updated national guidelines. See page 11 for other amendments during the COVID-19 pandemic.
- The **study starts after giving birth** and **runs up-to 6-12 months** after discharge from hospital and **both the intervention and control group will have 4 study visits** as part of the trial.

Why we are doing this study?

High blood pressure disorders in pregnancy are associated with an increased risk of high blood pressure, heart attack and stroke in later life. The risks associated with high blood pressure in pregnancy can be mitigated by early recognition and treatment of raised blood pressure (and other traditional risk factors e.g. lack of regular exercise, an unhealthy diet. This trial is looking at the impact that blood pressure control has on these long-term risks. We plan to assess whether blood pressure self-management can improve blood pressure control and whether this reduction in blood pressure in the months after birth can reduce the long-term effects these conditions have on your heart, brain and blood vessels, in turn reducing the risk of the events listed below, which include:

1 in 5 women having another hypertensive pregnancy

1 in 7 women having another pre-eclamptic pregnancy

A 4x increase in risk of having long term high blood pressure

A 2x increase in risk of experiencing both cardiovascular death or a heart attack

A 1.5x increase in risk of having a stroke

(Figure adapted from NICE NG 133: Hypertension in pregnancy July 2019)

Why have I been invited?

If you have pre-eclampsia or gestational hypertension and still require medication after delivering your baby/babies at the time of discharge.

Do I have to take part?

No, this is a voluntary study, and it is your decision to participate. You are free to withdraw from the study at any time without giving a reason. This would not affect the standard of care you receive. If you decide that you no longer wish to continue with the study, we would still retain any data already obtained from you unless you request otherwise.

What will happen if you take part in the POP-HT study:

Both groups receive the same number of study visits i.e. 4 visits for all participants.

For peer review only

Visit 1 (90 minutes) will take place in the first days after giving birth whilst you remain on the post-natal ward in the Women's centre. We will **measure your blood pressure**, **scan your heart (using an ultra-sound scan like you had of the baby), take a blood test and review your medical notes and blood tests** and e-mail a **questionnaire about your lifestyle and diet,** which can be completed later to shorten the visit**. At the end of visit 1** you will either be **allocated at random** to the **intervention group** or **the control group.** If allocated to the **intervention** group a separate member of the team will come and provide you with the blood pressure monitor and install the app on your smartphone/tablet and teach you how to use it. You will have plenty of time to practice whilst still in hospital!

Visits 2 and 3 (30 minutes) take place at **weeks 1 and 6 weeks after discharge respectively** and are to measure your blood pressure, take some simple measurements e.g. waist, left arm and hip circumference, and to complete a brief questionnaire. These can be done as a **home visit**, or as a visit to us in the Cardiovascular Clinical Research Facility (CCRF) at the **John Radcliffe Hospital**. At the end of **Visit 3** (week 6) there will be a 24 hour blood pressure monitor fitted, programmed to be silent to minimise disruption to you and your baby, and we will provide you with a stamped, addressed envelope to post it back to us at CCRF.

At 6-12 months there will be a **slightly longer visit to our research facility** (CCRF) in the **John Radcliffe Hospital. Visit 4** (up to 4 hours) will involve measuring your blood pressure again, doing another scan of your heart by ultra-sound, doing an MRI (magnet scan) of your heart and brain, and taking a blood test. There will also be a brief review of your medical and obstetric history and medications, and a few other tests (not absolutely mandatory), which include: taking photos of the blood vessels in the back of your eyes, and doing some gentle exercise on a bike (akin to walking up a hill at a fast pace) during which we measure your heart rate, blood pressure and, scan your heart briefly with the ultra-sound machine. The study finishes with another period of 24hr blood pressure monitoring and a wrist-watch (accelerometer) you wear for 1 week. ***The additional PIS you have also been given provides more detail specifically about this 4th visit. We will run through this again at the end of your 3rd visit at 6 weeks so you can have a chance to discuss any questions and we can help plan child care for the 4th and final longer visit at the John Radcliffe Hospital.***

We would like to follow you up for up to 10 years. For this longer term follow up, the research team in Oxford will ask for information about your health from NHS Digital (we will send your name, date of birth, NHS number and postcode to NHS Digital and ONS (or other central NHS bodies) who can link this information to your centrally held records to allow your blood pressure records to be reviewed if needed. We will access these records so that we can assess long term health outcomes and in particular monitor your long term blood pressure control in line with one of the study objectives.

We may also want to measure your blood pressure again in the future (up to 10 years from enrolment) as a home visit/visit to the hospital. This will be an extension to this study and we would like to contact you again to ask you to consider further participation.

Further details of the individual study procedures over the 6-month period are as follows:

1. Bed-side blood pressure measurement (10 minutes): Three blood pressure readings will be taken at intervals of 1 minute from your left arm (unless there is a medical reason not to use the left) using an automated blood pressure monitor. This will require you sitting at rest for 5 minutes prior to doing any measurements.
2. Echocardiogram scan (15 minutes): We will perform an ultrasound (echocardiogram/echo) of your heart. This is a safe and painless procedure and you will be asked to lie on a couch on your left side. A probe is placed on your chest and lubricating jelly is used so the probe makes good contact with the skin. Ultrasound waves then create images of your heart on the scanner monitor. It normally takes 15 minutes to acquire these images. A female sonographer/scanner will be provided wherever possible and if not available, a female chaperone will be available.
3. Vicorder® (Vascular Measures and Central Blood Pressures, 10 minutes): This involves lying flat on a couch and having two blood pressure cuffs fitted, one to the right arm and one to the right leg. These are inflated and deflated three times at 1-2 minute intervals. This is now an optional measure during the COVID-19 pandemic.
4. Lifestyle and diet questionnaire (25 minutes): The questionnaire combines validated questions used in previous studies. Information will be collected on factors that affect blood pressure including: smoking frequency, alcohol and salt intake, exercise and family history. Questionnaires can be completed either during a study visit or at a later date and posted back to the study team (pre-paid envelopes will be provided). Some of these questions may not seem relevant as they are taken from validated questionnaires used across a range of ages and in both males and females. The team will explain any such questions.
5. EQ-5D-5L Quality of Life questionnaire (5 minutes): You will be provided with an EQ-5D-5L questionnaire, a widely used and validated way of assessing quality of life. A trained study investigator will run through the structured questionnaire during the visit with you.
6. MRI of the Heart, Brain, aorta and kidneys (1 hour including break): As part of this study you will have an MRI scan of your heart and brain. The MRI scanner is shaped like a polo mint, the hole inside measuring about 60 centimetres wide. MRI is safe and non-invasive and does not involve any ionising radiation (x-rays). However, because they use a large magnet to work, MRI scans are not suitable for everybody. Because of this, you will be asked pre-screening safety questions to help determine if you are able to take part. More detail about the MRI scan is provided on the *Supplementary Information Sheet* given to you before the 4th study visit. This additional information sheet also provides more detail about the optional sub-study of having gadolinium given (a commonly used contrast drug for MRI) to acquire an extra few images if you are no longer breast-feeding at that time-point. Additional consent will be sought and obtained for this sub-study prior to the scan being performed.
7. Blood test (10 minutes): The equivalent of 5 teaspoons of blood will be collected by a trained member of staff. We will try our best to time it with blood tests requested by your clinical care team whilst you are an inpatient. You do not need to be fasted for this test. We use very small 'butterfly' needles to minimise any discomfort. You may experience some bruising and

For peer review only

discomfort at the site where you have your blood taken. Our staff are highly trained in blood taking and we will make sure you are as comfortable as possible.

8. Fitting of a home blood pressure monitor (5 minutes, to be worn for 24 hours) and activity monitor (5 minutes, to be worn for 7 days and nights): This monitor consists of a blood pressure cuff, which will be fitted on the left arm (right if a specific medical reason precludes use of the left) and a BP monitor. A small bag will also be provided that is worn around the waist or shoulders, in which the monitor is placed. You will be shown how to re-attach the cuff to the monitor e.g. after a bath/shower. The BP monitor will be silent and automatically inflate hourly during the day and every other hour at night to minimise inconvenience at this busy time. Participants will be asked to wear the monitor for 24 hours. We provide you with an information sheet about how to use the monitor and deal with frequent problems and this also has a brief blood pressure monitoring diary on the back for you to detail the time you went to sleep/woke up and any periods of activity that may have put your blood pressure up e.g. running for the bus/cycling to work. The activity monitor (wrist-watch) is waterproof and shock-proof and will be worn continually on the wrist for one week. Stamped, addressed envelopes will be provided to return both devices after use.
9. Retinal imaging (10 minutes): Photos will be taken of the back of your eye just like at an optician. More information about this is provided in the *Supplementary Information Sheet*. To help keep your information confidential, your images will be 'de-identified' and assigned a study code. However, your retinal images are unique to you so they can never be completely anonymous.
10. Cardiopulmonary Exercise Testing (CPET) with exercise echo (30 minutes): This involves gentle cycling on a stationary bicycle and doing a short ultrasound (echocardiogram) of your heart whilst exercising. More information about this is provided in the *Supplementary Information Sheet*.

The Self-management (intervention) group: Taking your blood pressure at home

This section only applies to participants who are allocated to the intervention group and an additional intervention group information sheet will be provided for you as a paper copy, on the app and on the website.

You will be asked to **start measuring your blood pressure** on the **day of discharge** from hospital using the OMRON Evolv monitor provided. This will mean taking **2 readings, 1 minute apart** every morning after discharge. **We will ask you to do this every morning, until you have had 5 consecutive days with blood pressure readings in the normal range (off medication).** We anticipate this to take approximately **2-3 weeks following discharge** based on our experience in our pilot study.

After you measure your blood pressure, you will **open up the POP-HT app** on your smartphone/tablet, confirm it was you that took the blood pressure readings and **click 'SYNC'.** The app will then 'synchronise' with the OMRON EVOLV® monitor to **upload the reading to the app and the secure study website** hosted on the hospital's secure intranet (*not the internet*). **See figure one below for an illustration of how this is done**

For peer review only

Your smartphone/tablet will notify you if your readings are too high, too low or in the normal range. If they are high or low the app will ask you to repeat one more reading. If the reading is still high/low the app will notify you and the specialist study doctors. **You can then opt to receive a call from them to help adjust your medication and will be advised to make an urgent/same-day appointment with your own GP/midwife if necessary** (further detailed information about this is available in the self-management information sheet).

Medication will be gradually titrated down until you are off all medication.

Once you have 5 days in a row with readings in the normal range (off medication) you will be sent a notification/message reading "Thank you. Your blood pressure readings have all been normal since stopping treatment. Please **change to once weekly readings** for the remainder of the study." This will be **until 6 months** after the delivery of your baby. The **reason** for the **longer period of weekly monitoring** is to **ensure you do not have a late rise in your blood-pressure readings** that requires further treatment.

For peer review only

Figure 1: Illustration of how self-monitoring will be performed in the intervention group

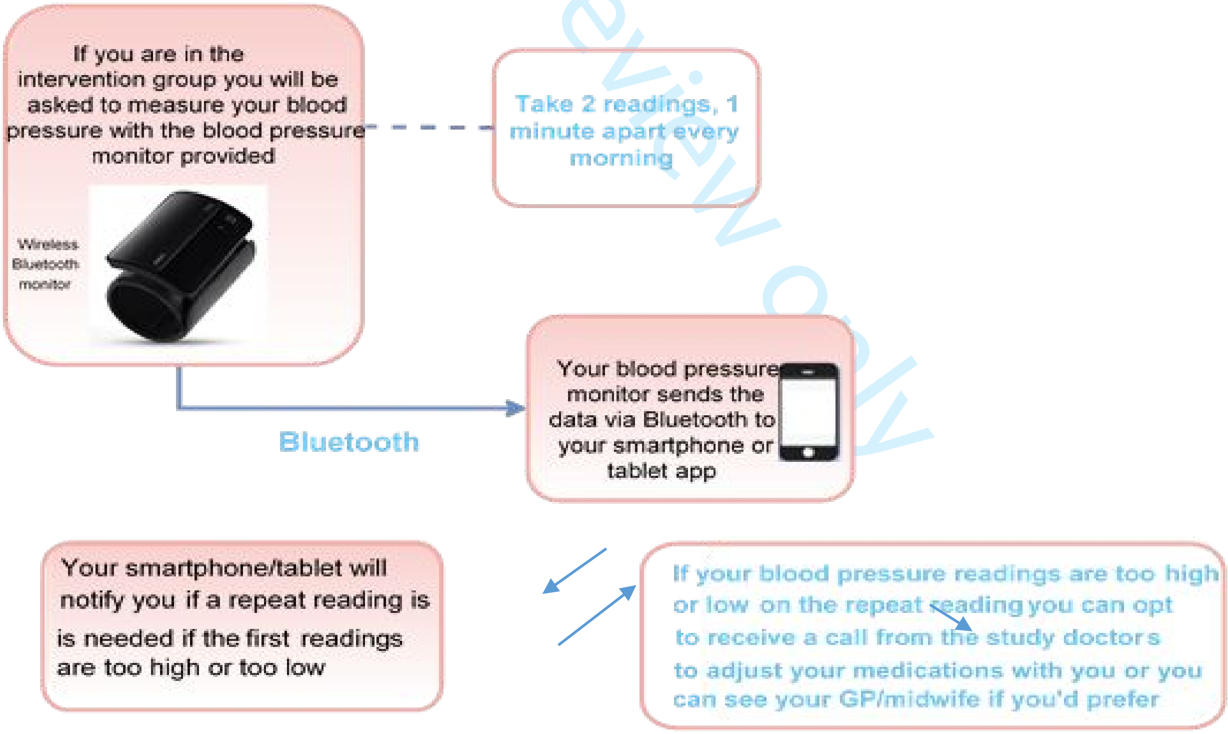

The control group

If you are allocated to the control group, you will be looked after by your GP/midwife/Health visitor as per usual NHS care in addition to the 4 study visits described above. **You will also be given a blood pressure monitor for the first few weeks, during the COVID-19 pandemic, as per updated national guidance**. This is to facilitate care from your GP and mid-wife who may not be able to do home visits as they normally would during the pandemic. Instead, your self-monitored readings can be reviewed by a tele-medicine appointment (when a face-face review is not possible due to COVID-19) and your medications adjusted as needed.

What will I need to do if I want to take part?

You will have plenty of time to consider your participation after reading this. If you decide you want to take part, the first step is to contact the research team who will arrange your first study visit.

Contact: Dr Jamie Kitt or Mrs Yvonne Kenworthy Tel: 01865 572833 or study mobile 07713 782185

E-mail: Jamie.kitt@cardiov.ox.ac.uk or Yvonne.Kenworthy@cardiov.ox.ac.uk

You can contact us by calling/SMS/e-mail to arrange a study visit, or we will contact you after giving you at least one hour to consider this document. If you are willing to participate in the study, then we will take your written consent.

What should I consider?

You will not be able to take part in this study if the chief investigator deems your taking part would be unsafe and will already have been screened against strict exclusion criteria. Should you have any concerns however, please discuss this with the study team. Due to the fact that the nature of this research study involves home visits, all members of the research team have undertaken the relevant and stipulated safe-guarding training.

Are there any possible disadvantages or risks from taking part?

Your decision on whether you will participate or not **will not affect in any way your clinical care** now or in future. All of the study procedures/assessments are safe but as with any medical procedure, there are some minor risks. More details on the risks associated with certain procedures e.g. cardiac MRI and blood taking are detailed in the ***Supplementary Information Sheet provided with this main PIS***. At all times, an experienced study investigator will be with you and will address any issues that may arise. As per routine clinical advice women in both arms of the trial should continue to monitor their babies for drowsiness, lethargy, pallor, cold peripheries or poor feeding when discharged home.

What are the possible benefits of taking part?

This study is designed to test whether self-management can improve blood pressure control in the period immediately after birth. As part of the study all participants receive 4 additional visits above and beyond usual NHS care and so will be more closely monitored than you otherwise would be. This may result in earlier access to treatment should any abnormal BP readings be detected.

For peer review only

Will my taking part in this study be kept confidential?

Yes. The data collected from the study will be de-identified so that you will be known only by a unique study specific ID. You would not be identifiable from this. Responsible members of the University of Oxford, Oxford University Hospitals NHS Trust and Regulatory Authorities may be given access to data for monitoring and/or audit of the study to ensure that the research is complying with applicable regulations. As part of our commitment to maximise participant involvement in research, participants can give consent (optional) for their contact details to be retained (see section below). As mentioned above, details will be shared with NHS digital to allow longer-term follow up of your blood pressure up to 10 years. NHS digital is regulated by the same strict criteria as this study. In the event that you lose capacity to consent whilst taking part in the study you will be withdrawn from the study and no further data will be collected nor any further assessments/procedures undertaken. Any data that has already been collected will be retained.

What will happen to the samples I give?

Blood samples collected will be analysed for this study, but your samples may also be used for other studies with appropriate ethical approval in the future. Samples collected will be de-identified and stored in secured facilities within the University of Oxford. If you withdraw from the study for any reason, we will retain any blood samples and data collected up to that point for use in research as detailed in this participant information sheet. If you agree to your samples being used in future research, your consent form will be held until the samples have been depleted or destroyed.

Will my General Practitioner/family doctor (GP) be informed of my participation?

Your GP will be notified of your study participation and will be provided with a letter or study information sheet. There may also be instances where GPs will be contacted to follow up incidental findings that may be of clinical significance or if you withdraw/are withdrawn from the study.

What will happen to my data?

Data protection regulation requires that we state the legal basis for processing information about you. In the case of research, this is 'a task in the public interest.' The University of Oxford is the data controller and is responsible for looking after your information and using it properly. We will be using information from you, your medical notes and NHS Digital (and other central NHS bodies) in order to undertake this study and will use the minimum amount of personally-identifiable information possible. We will store any research documents with personal/traceable data, such as consent forms and your retinal images, securely at the University of Oxford for 10 years after the end of the study as part of the research record. If you have consented to your samples being retained for future research, a copy of your consent is retained for the duration of sample storage. We keep any other identifiable information about you for up to 12 months after the study is finished. All documents containing personal information such as your informed consent form will be stored securely and only accessible by study staff and authorised personnel only. The Oxford University Hospitals NHS Foundation Trust will use your name, NHS number, date of birth

and contact details (address and telephone number) to contact you about the research study, to make sure that the relevant information about the study is recorded for your care, and to oversee the quality of the study. They will keep identifiable information about you from this study for up to 12 months after the study has finished. As part of your participation in the study, in addition to the information you provide about your health, the research team in Oxford will ask for information about your health from NHS Digital (including, but not limited to, NHS Digital). We will send your name, date of birth, NHS number and postcode to NHS Digital (or other central NHS bodies) who can link this information to your centrally held records. Data protection regulation provides you with control over your personal data and how it is used. When you agree to your information being used in research, however, some of those rights may be limited in order for the research to be reliable and accurate. Further information about your rights with respect to your personal data is available at http://www.admin.ox.ac.uk/councilsec/compliance/gdpr/individualrights. You can find out more about how we use your information by contacting jamie.kitt@cardiov.ox.ac.uk.

Will I be reimbursed for taking part?

If you visit us at CCRF we will reimburse travel and parking expenses to and from the CCRF at the John Radcliffe Hospital site, if you provide receipts and/or mileage details. **You will also receive £30 thank-you** for your participation in the study after the final study visit. The app notifications and usage are free when in WIFI zone but if using 3G/4G they may be charged depending on your network-provider. If this is the case, then any cost incurred will be reimbursed to you on production of the relevant bill.

What will happen if I don't want to carry on with the study?

You can withdraw from the study at any time without giving a reason. If you decide you no longer wish to take part in our study, you can phone, write to, or e-mail Dr Jamie Kitt using the contact details listed in the header on page one. Should you wish to withdraw, please let us know if we can keep the information we have collected about you so far as we may be unable to destroy the data if it has already been de-identified, as outlined in the confidentiality section. Data and samples already collected would not be used in the final study analysis except where analysis of their data or samples has already been integrated into interim results.

What will happen to the results of the study?

Summarised results will be published in scientific journal/s and also summarised on our website, after completion of the study, for you to read: https://www.rdm.ox.ac.uk/about/our-clinical-facilities-and-mrc-units/cardiovascular-clinical-research-facility/ongoing-clinical-studies.

What if we find something unexpected?

If your blood pressure readings are very high (**above 160/110mmHg**), we will inform you of this finding immediately with instructions on what to do next and we will also call you to inform you to seek medical assistance. In the unlikely event that we detect any structural abnormalities during the scan of your heart

(echocardiogram) or MRI of your heart or brain, then with your permission we will refer you for assessment by contacting your GP and/or a specialist hypertension clinic, who can arrange instigate any necessary investigations and treatment.

What if there are any problems?

The University of Oxford, as Sponsor, has appropriate insurance in place in the unlikely event that you suffer any harm as a direct consequence of your participation in this study. NHS indemnity operates in respect of the clinical treatment which is provided. If you wish to complain about any aspect of the way in which you have been approached/treated during this study, you should contact, the Chief Investigator, Prof Paul Leeson on +44 (0)1865 572846 or e-mail: paul.leeson@cardiov.ox.ac.uk. You may contact the University of Oxford Clinical Trials and Research Governance (CTRG) office on 01865 616480, or the head of CTRG, email ctrg@admin.ox.ac.uk.

How have patients and the public been involved in this study?

Members of the public, who have been through similar pregnancy related medical problems, have been involved in the design of this study, testing and refining of the intervention arm being trialled and several of the documents including the poster, logo and this patient information leaflet.

Who is organising and funding the research?

This study has been designed and organised by investigators of the University of Oxford, Division of Cardiovascular Medicine and Nuffield Department of Primary Care Health Sciences (namely Prof Paul Leeson, Dr Jamie Kitt, Professor Richard McManus, Dr Lucy Mackillop and Dr Adam Lewandowski). If you wish to know more about any aspect of the study, please contact Jamie Kitt on 01865 (5)72833 or jamie.kitt@cardiov.ox.ac.uk. Dr Jamie Kitt is conducting this research as part of his doctoral studies and results from this study may be used in anonymous form to support this. The research is being financed by the British Heart Foundation. The sponsor of the study is the University of Oxford.

Who has reviewed the study?

All research in the NHS is looked at by an independent group of people called a Research Ethics Committee, to protect your safety, rights, wellbeing and dignity. This study has been reviewed by London Surrey REC [19/LO/1901].

Participation in future research:

If you consent to be considered for future studies, a copy of this consent and your contact details will be kept securely and independently of the study records in a separate, secure database in order that we can contact you if further research in this area is being under-taken. You are under no obligation to consent to being contacted again and we understand you may want to participate only in this study in which case your details will not be kept as explained in the data confidentiality section above. You have the right to ask for your personal information to be removed from this database at any time.

Further information and contact details:

For peer review only

Lead Study Investigator: Dr Jamie Kitt; Tel: 01865 572833 Email: jamie.kitt@cardiov.ox.ac.uk

Chief Investigator: Prof Paul Leeson; Tel: 01865 572846 Email: paul.leeson@cardiov.ox.ac.uk

Amendments during COVID-19 pandemic

During the COVID-19 pandemic, this PIS and the flyer will be provided electronically via a tablet, which the clinical team will give you, although there will be less time to consider this than normal due to expedited discharge processes during the pandemic. Consent forms will be placed in wipe down wallets, which will be handed to you for signing and then photocopied whilst wearing gloves. The copy will be placed back into a sterile wallet for you and the original will be placed in a second wallet. Both will be wiped down with CLINELL® wipes and our copy will be kept securely in quarantine before moving them to CCRF. The baseline visit has been adjusted to reduce the amount of direct patient contact to the blood pressure measurements, the ultra-sound (echo), and the Vicorder test is now optional. There will not be a second female chaperone from the research team (this will not affect normal hospital chaperone rules). Questionnaires are e-mailed out and can be completed on a tablet/computer at a later date. All direct contact will be done in PPE where necessary in line with hospital policy. The control arm will also be provided with a blood pressure monitor, when NHS monitors are not available prior to discharge to allow home monitoring by NHS GPs/mid-wives in line with updated RCOG guidance. The study will be performing remote follow up visits for week 1 and week 6 (visits 2 and 3). The final (4th visit) is being extended from 6-9 months, to 6-12 months by which time 'normality' will hopefully have been resumed to allow the final visit to take place at the John Radcliffe Hospital. In extenuating circumstances, such as COVID-19 national lockdowns some part of the V4 will also be done remotely, the 24hr BP monitor fitting, the accelerometer/wrist-watch fitting, the review of the medical history, demographics, questionnaires, and manual blood pressure measurements may also be conducted remotely via video or phone call. The remaining procedures of the final visit will be done when it is safe to do within the 12 month time-window.

For peer review only

Tel: 01865 572833
Email: jamie.kitt@cardiov.ox.ac.uk
Fax: +44(0)1865 572840

Study Code:			*Participant identification number:*		
P	O	—			

Physician Optimised Post-partum Hypertensive Treatment (POP-HT) Study

CONSENT FORM

Name of Researcher:

Participant Name:

If you agree, please initial each box

1. I confirm that I have read the information sheet dated V3.0 06/05/20 for this study. I have had the opportunity to consider the information, ask questions and have had these answered satisfactorily.	
2. I understand that my participation is voluntary and that I am free to withdraw at any time without giving any reason, without my medical care or legal rights being affected.	
3. I understand that relevant sections of my medical records and data collected during the study may be looked at by individuals from University of Oxford, hosting NHS organisations and regulatory authorities, where it is relevant to my taking part in this research. I give permission for these individuals to have access to my records.	
4. MRI/Echocardiography/other research tests: I understand that these are research scans/tests that are not useful for medical diagnosis, and that scan/test results are not routinely looked at by a doctor. If a concern is raised about a possible abnormality on my scan/research test, I will only be informed if a doctor thinks it is medically important such that the finding has clear implications for my current or future health.	
5. I agree for my GP to be informed of any results of medical tests performed as part of the research that may be important for my health care.	
6. If I withdraw/I am withdrawn from the study we will contact your GP to inform them, in order to ensure that any on-going care you require is reinstated.	
7. I agree that the information held and maintained by NHS Digital/ Office for National Statistics (ONS) may be used to provide information about my health status. I understand that my name, NHS number, date of birth and postcode may be shared securely to obtain such information and allow contact for blood pressure, and other relevant measurements over the next 10 years.	

For peer review only

8. I agree to donate blood samples. I consider these samples a gift to the University of Oxford and I understand I will not gain any direct personal or financial benefit from them.		
9. I understand that retinal images will be taken as part of the study and will be stored in a de-identified format on the high compliance (secure) server of the University of Oxford for up to 10 years		
10. I agree to take part in this study		
Additional:	Yes	No
11. I agree for my anonymised samples to be used in future research, here or abroad, which has ethics approval. I understand this research may involve commercial organisations.		
12. I agree to be contacted about ethically approved research studies for which I may be suitable. I understand that agreeing to be contacted does not oblige me to participate in any further studies.		
13. I have been approached about the optional additional measure: Vicorder ® assessment of the aortic stiffness and aortic blood pressure		
14. I have been approached about the optional additional measure: Laser Speckle Tracking of the skin on my fore-arm and agree to partake in this additional measure (being done in 48 participants out of 200)		

Name of Participant	*Date*	*Signature*
Name of Person taking Consent	*Date*	*Signature*

**** For researchers please tick (✓) to document:***

The original signed form will be placed in the medical notes and a further copy will be retained at the trial site and one given to the participant ( )

For peer review only

SPIRIT

STANDARD PROTOCOL ITEMS: RECOMMENDATIONS FOR INTERVENTIONAL TRIALS

SPIRIT 2013 Checklist: Recommended items to address in a clinical trial protocol and related documents*

Section/item	Item No	Description
**Administrative information**		
Title	1	A Randomised control trial of post-partum blood pressure self-management following hypertensive pregnancy: Physician Optimised Post-partum Hypertension Treatment (POP-HT) trial protocol paper
Trial registration	2a	NCT04273854 at Clinicaltrials.gov
	2b	All items from the World Health Organization Trial Registration Data Set
Protocol version	3	V6.0 08/03/2021
Funding	4	BHF Grant number FS/19/7/34148
Roles and responsibilities	5a	See title page and section on authors contributions
	5b	See title page
	5c	See section titled Publication Policy: Role of study sponsor and funders, if any, in study design; collection, management, analysis, and interpretation of data; writing of the report; and the decision to submit the report for publication, including whether they will have ultimate authority over any of these activities
	5d	See section on Trial Monitoring: Composition, roles, and responsibilities of the coordinating centre, steering committee, endpoint adjudication committee, data management team, and other individuals or groups overseeing the trial, if applicable (see Item 21a for data monitoring committee)
**Introduction**		
Background and rationale	6a	See Introduction section
	6b	
Objectives	7	See Hypotheses section

For peer review only

Trial design	8	See section on Study design within METHODS

Methods: Participants, interventions, and outcomes

Study setting	9	See Section titled ‘Study design’
Eligibility criteria	10	See Section titled ‘Eligibility and recruitment
Interventions	11	SEE METHODS AND APPENDICES A-E
Outcomes	12	See Section titled ‘Study aims and objectives’
Participant timeline	13	See FIGURE 1 within METHODS and APPENDICES A-C
Sample size	14	See Section titled ‘DATA ANALYSIS’
Recruitment	15	See METHODS

Methods: Assignment of interventions (for controlled trials) :

Sequence generation	16a	See Section titled ‘Randomisation’. More detail in full study protocol and SAP
Allocation concealment mechanism	16b	See Section titled ‘Randomisation’. More detail in full study protocol and SAP
Implementation	16c	See Section titled ‘Randomisation’. More detail in full study protocol and SAP

Methods: Data collection, management, and analysis: See Sections titled ‘Assessments during study visits’, ‘Data analysis’ and ‘SAP’

Data collection methods	18a	Plans for assessment and collection of outcome, baseline, and other trial data. DATA collection is all electronic using CASTOR® EDC.
	18b	See section on PPI and SAP and further detail in the full study protocols

Data management	19	See section titled 'Data Management'. Full plans for data entry, coding, security, and storage are detailed in the full study protocol and summaries in the participant information sheet (PIS)
Statistical methods	20a	See SAP summary within the protocol paper. Full SAP to be produced
	20b	Full SAP to be produced imminently
	20c	Full SAP to be produced imminently
**Methods: Monitoring: See sections titled 'TRIAL OVERSIGHT' and 'SAFETY REPORTING'**		
Data monitoring	21a	See 'Trial Committees'
	21b	Description of any interim analyses and stopping guidelines, including who will have access to these interim results and make the final decision to terminate the trial
Harms	22	See ' Trial oversight and Safety Reporting sections'
Auditing	23	See ' Monitoring' section
**Ethics and dissemination: See Section titled 'Ethics and Dissemination' and Supplementary Material Appendices E and F**		
Research ethics approval	24	19/LO/1901 is the RE/HRA approval reference
Protocol amendments	25	See Supplementary materials: appendices E and F
Consent or assent	26a	Detailed in full trial protocol in more detail but summarised in Section 'Eligibility and Recruitment' and 'Ethics and Dissemination'
	26b	See Appendices section below re consent for biological specimens
Confidentiality	27	See section on 'Participant Confidentiality'
Declaration of interests	28	See section titled 'Competing interests'
Access to data	29	See full trial protocol for section on data access, storage and management
Ancillary and post-trial care	30	See section titled Insurance
Dissemination policy	31a	See section titled Publication policy
	31b	See section titled Publication policy
	31c	See section titled Publication policy

For peer review only - http://bmjopen.bmj.com/site/about/guidelines.xhtml

**Appendices:**		**Appendices are referenced in the main body of the text where relevant and are uploaded in the Supplementary file.**
Informed consent materials	32	See ‘Appendix G’ in the supplementary file
Biological specimens	33	See relevant sections of the consent forms and PIS in Appendix G of the supplementary file. Further details are also contained in the full study protocol

*It is strongly recommended that this checklist be read in conjunction with the SPIRIT 2013 Explanation & Elaboration for important clarification on the items. Amendments to the protocol should be tracked and dated. The SPIRIT checklist is copyrighted by the SPIRIT Group under the Creative Commons “Attribution-NonCommercial-NoDerivs 3.0 Unported” license.

For peer review only

BMJ Open

Post-partum blood pressure self-management following hypertensive pregnancy: protocol of the Physician Optimised Post-partum Hypertension Treatment (POP-HT) trial

Journal:	*BMJ Open*
Manuscript ID	bmjopen-2021-051180.R1
Article Type:	Protocol
Date Submitted by the Author:	17-Oct-2021
Complete List of Authors:	Kitt, Jamie; University of Oxford<br>Frost, Annabelle; University of Oxford<br>Mollison, Jill; University of Oxford, Nuffield Department of Primary Care Health Sciences<br>Tucker, Katherine; University of Oxford, Nuffield Department of Primary Care<br>Suriano, Katie; University of Oxford<br>Kenworthy, Yvonne; University of Oxford, Oxford Cardiovascular Clinical Research Facility<br>McCourt, Annabelle; University of Oxford<br>Woodward, William; University of Oxford<br>Tan, Cheryl; University of Oxford<br>Lapidaire, Winok; University of Oxford<br>Mills, Rebecca; University of Oxford, Radcliffe Department of Medicine<br>Khan, Maryam; University of Oxford, Radcliffe Department of Medicine<br>Tunnicliffe, Elizabeth M; University of Oxford, Radcliffe Department of Medicine<br>Raman, Betty; University of Oxford, Radcliffe Department of Medicine<br>Santos, Mauro; University of Oxford, Institute of Biomedical Engineering, Department of Engineering Science<br>Roman, Cristian; University of Oxford, Institute of Biomedical Engineering, Department of Engineering Science<br>Hanssen, Henner; University of Basel<br>Mackillop, Lucy; University of Oxford, Nuffield Department of Women's and Reproductive Health<br>Cairns, Alexandra; University of Oxford, Nuffield Department of Women's and Reproductive Health<br>Thilaganathan, Basky; University of London Saint George's, Obstetrics and Gynecology<br>Chappell, Dr Lucy; King's College London, Women's Health Academic Centre<br>Aye, Christina; University of Oxford, Nuffield Department of Women's and Reproductive Health<br>Lewandowski, Adam; University of Oxford, Division of Cardiovascular Medicine, Radcliffe Department of Medicine<br>McManus, Richard; University of Oxford, Dept of Primary Care Health Sciences<br>Leeson, Paul; University of Oxford, Division of Cardiovascular Medicine

**Primary Subject Heading**:	Cardiovascular medicine
Secondary Subject Heading:	Cardiovascular medicine
Keywords:	Hypertension < CARDIOLOGY, Echocardiography < CARDIOLOGY, Cardiology < INTERNAL MEDICINE, Vascular medicine < INTERNAL MEDICINE, Maternal medicine < OBSTETRICS

SCHOLARONE™ Manuscripts

Post-partum blood pressure self-management following hypertensive pregnancy: protocol of the Physician Optimised Post-partum Hypertension Treatment (POP-HT) trial

Dr Jamie Kitt[1] BM BCh MA MRCP, Dr Annabelle Frost[1] MChB MRCOG, Dr Jill Mollison[5] PhD, Dr Katherine Tucker[5] BSc (Hons) PhD, Dr Katie Suriano[1] PhD, Mrs Yvonne Kenworthy[1] BSc, Mrs Annabelle McCourt[1], Mr William Woodward BSc[1], Mrs Cheryl Tan[1] BSc(Hons) MRes, Dr Winok Lapidaire[1] MSc PhD, Mrs Rebecca Mills MSc[6,] Ms Maryam Khan BSc[6], Dr Elizabeth M Tunnicliffe[6] PhD, Dr Mauro Santos[7] D Phil, Dr Cristian Roman[7] D Phil, Professor Henner Hanssen[8] MD, Dr Lucy Mackillop[2] BM Bch MA, Dr Alexandra Cairns BM BCh MRCOG[2], Professor Basky Thilaganathan[3] FRCOG, Professor Lucy Chappell[4] FRCOG, Dr Christina Aye[2] MRCOG DPhil, Dr Adam Lewandowski[1] BSc (Hons) DPhil, Professor Richard McManus[4] FRCGP PhD and Professor Paul Leeson[1] PhD FCRP

1 Cardiovascular Clinical Research Facility (CCRF), Division of Cardiovascular Medicine, University of Oxford, Oxford, UK
2 Nuffield Department of Women's and Reproductive Health, University of Oxford, Oxford, UK
3 Maternal-fetal Medicine Unit, St George's University Hospitals NHS Foundation Trust
4 King's College London
5 Nuffield Department of Primary Care, University of Oxford, Oxford, UK
6 Oxford Centre for Clinical Magnetic Resonance Research, Division of Cardiovascular Medicine, Radcliffe Department of Medicine, University of Oxford, Oxford, UK.
7 Oxford Institute for Biomedical Engineering, University of Oxford, Oxford, UK
8 Department of Sport, Exercise and Health, University of Basel, Switzerland

Correspondence to:
Dr Jamie Kitt, Oxford Cardiovascular Clinical Research Facility, Division of Cardiovascular Medicine, Radcliffe Department of Medicine, University of Oxford, John Radcliffe Hospital, Oxford. OX3 9DU, UK.
Tel: +44 1865 572833. Fax +44 1865 221111. E-mail: jamie.kitt@cardiov.ox.ac.uk

Date: 12/10/2021

Word Count: 6, 904 (excluding title page, abstract, figures, tables, references and appendices)

Protocol version: V 6.0 08/03/2021

Sponsor: University of Oxford Clinical Trials and Research Governance, Joint Research Office, 1st floor, Boundary Brook House, Churchill Drive, Headington. Oxford OX3 7GB Tel: 01865 61648
E-mail : ctrg@admin.ox.ac.uk

Funders: The research is being financed by a British Heart Foundation Clinical Research Training Fellowship (BHF Grant number FS/19/7/34148).

For peer review only

ABSTRACT

Introduction

New onset hypertension affects approximately 10% of pregnancies and is associated with a significant increase in risk of cardiovascular disease in later life, with blood pressure measured six weeks post-partum predictive of blood pressure 5-10 years later. A pilot trial has demonstrated that improved blood pressure control via self-management during the puerperium was associated with lower blood pressure for up to three years. POP-HT will formally evaluate whether improved blood pressure control in the puerperium results in lower blood pressure at six months post-partum, and improvements in cardiovascular and cerebrovascular phenotypes.

Methods and analysis

POP-HT is an open label, parallel arm, randomised controlled trial involving 200 women aged 18 years or over, with a diagnosis of pre-eclampsia or gestational hypertension, and requiring anti-hypertensive medication at discharge. Women are recruited by open recruitment and direct invitation around time of delivery and randomised 1:1 to either an intervention comprising physician-optimised self-management of post-partum blood pressure or usual care. Women in the intervention group upload blood pressure readings to a 'smartphone' app that provides algorithm-driven individualised medication-titration. Medication changes are approved by physicians, who review blood pressure readings remotely. Women in the control arm follow assessment and medication adjustment by their usual health care team. The primary outcome is 24 hour average ambulatory diastolic blood pressure at 6-9 months post-partum. Secondary outcomes include: additional blood pressure parameters at baseline, week 1 and week 6; multi-modal cardiovascular assessments (CMR and echocardiography); parameters derived from multi-organ magnetic resonance imaging including brain and kidneys; peripheral macrovascular and microvascular measures; angiogenic profile measures taken from blood samples and levels of endothelial circulating and cellular biomarkers; and objective physical activity monitoring and exercise assessment. An additional 20 women will be recruited after a normotensive pregnancy as a comparator group for endothelial cellular biomarkers.

Ethics and dissemination:

IRAS PROJECT ID 273353. This trial has received a favourable opinion from the London - Surrey Research Ethics Committee and HRA [REC Reference 19/LO/1901]. The Investigator will ensure that this trial is conducted in accordance with the principles of the Declaration of Helsinki and follow good clinical practice guidelines. The Investigators will be involved in reviewing drafts of the manuscripts, abstracts, press releases and any other publications arising from the study. Authors will acknowledge that the study was funded by the British Heart Foundation Clinical Research Training Fellowship (BHF Grant number FS/19/7/34148). Authorship will be determined in accordance with the ICMJE guidelines and other contributors will be acknowledged.

Trial Registration: Clinicaltrials.gov registration number (NCT04273854), registered 18TH February 2020.

Keywords: Blood pressure, Hypertension, Pre-eclampsia, Gestational hypertension, Hypertensive pregnancy, Randomised trial, Self-management, Cardiac imaging, Cardiac remodelling, Cerebrovascular health

For peer review only

STRENGTH AND LIMITATIONS OF THIS STUDY

- POP-HT is the first randomised trial that is powered to detect whether post-partum blood pressure self-management can significantly improve blood pressure control at 6-9 months post-partum, in women affected by new onset hypertension in pregnancy.
- POP-HT will also be the first randomised trial to investigate whether this improved blood pressure control translates into beneficial cardiovascular, vascular and cerebrovascular modelling, and will help elucidate the mechanisms behind adverse remodelling.
- The technology used to facilitate self-management in the study is readily translatable into widespread clinical practice, once it has been subject to the relevant regulatory approval and further validation required.
- The risk of drop-out, amplified by the COVID-19 global pandemic, could affect the ability to remain adequately powered to test the secondary outcome measures of the trial.
- The trial requires high levels of participant motivation and engagement during a busy time of the participants' lives. Patients in the intervention arm are required to submit daily readings when on treatment and adjust their medication as instructed by the app. Weekly readings are required thereafter for the duration of the study. This is in addition to the two periods of 24hr ABPM over the 6-month period. The degree of engagement required may limit recruitment into the trial and/or result in high drop-out/withdrawal rate.

For peer review only

ABBREVIATIONS

AE Adverse event
ABPM Ambulatory Blood pressure monitoring
AHA American Heart Association
CI/PI Chief/Principal Investigator
CCRF Cardiovascular Clinical Research Facility
CPET Cardiopulmonary exercise test
CT Clinical Trials
CTRG Clinical Trials and Research Governance
DIC Disseminated Intra-vascular Coagulation
DMC/DMSC Data Monitoring Committee / Data Monitoring and Safety Committee
DSUR Development Safety Update Report
ECV Extra-cellular volume
ESC European Society of Cardiology
GCP Good Clinical Practice
GP General Practitioner
HRA Health Research Authority
ICF Informed Consent Form
LV Left ventricle
MHRA Medicines and Healthcare products Regulatory Agency
NICE National institute for clinical excellence
OCMR Oxford Centre for Cardiovascular Magnetic Resonance Imaging
OIBME Oxford Institute for Biomedical Engineering
NHS National Health Service
NIHR National Institute for Health Research
PIS Participant/ Patient Information Sheet
PPE Personal Protective Equipment
PW Pulse wave
R&D NHS Trust R&D Department
REC Research Ethics Committee
SAE Serious Adverse Event
SAP Statistical Analysis Plan
SOP Standard Operating Procedure
TMF Trial Master File

For peer review only

INTRODUCTION

Hypertensive disorders of pregnancy affect 10% of pregnancies, which equates to >80,000 women per year in the UK [1]. One study showed that 50% of women with pre-eclampsia have persistent significant hypertension on day five following delivery [2], with blood pressure control remaining an issue for up to six weeks after childbirth, often requiring multiple medications and careful titration. At the same time, competing demands on mothers, not least from her newborn baby, are associated with poor adherence and/or poor levels of clinical contact. Drug titration can therefore be sporadic exacerbating poorly controlled hypertension. The risk of poor blood pressure control during this period may extend into later life. Hypertensive disorders of pregnancy are associated with a two-fold increase in risk of subsequent cardiovascular disease [3, 4] with a third presenting with chronic hypertension within 10 years [5]. We observed that high blood pressure during the first six weeks post-partum is related to the risk of higher blood pressure in the five to 10 years following a hypertensive pregnancy [6]. Furthermore, the SNAP-HT randomised controlled pilot study [7] showed blood pressure control in the post-partum period can be improved through self-management, with a mean improvement of 5.2mmHg in systolic and 6mmHg in diastolic blood pressure at six weeks post-partum. However, the most striking finding was that mean diastolic blood pressure remained 4.5mmHg lower in the intervention group at six months' post-partum; even after all but two mothers had stopped medication. Long-term follow-up of the women involved in the trial demonstrated diastolic blood pressure remained significantly lower in the intervention group [-6.8mmHg), at more than three years post-partum, even when adjusted for 'lifestyle risk factors' [8]. In those below the age of 50 years diastolic blood pressure is also a better correlate to long-term cardiovascular risk and is the predominant pathophysiology in young adults [9] before progression to the mixed and systolic patterns of hypertension seen in those over 50 [9]. Given all our patients are young females, below the age of 50 years, 24hr overall average diastolic blood pressure was selected as the primary outcome for our trial.

In the general population, high blood pressure is strongly related to long-term risk of cardiovascular disease and is the leading risk factor for loss of disability-adjusted life years (DALYs) in high and low-middle income countries. Every 10 systolic/5 diastolic mmHg of blood pressure reduction associates with a ~40% reduction in lifetime stroke risk and ~20% reduction in coronary heart disease risk [10]. If the difference observed in SNAP-HT could be translated to all women who have a hypertensive pregnancy, significant reductions in disease burden could be achieved in the population. However, several questions remain to determine the potential clinical translational benefits of self-monitoring in the management of post-partum blood pressure. Firstly, can a similar reduction in blood pressure be achieved with updated technology in a trial powered to detect diastolic BP differences at 6 months? Bluetooth-enabled home blood pressure monitors allow for automated upload of readings to the app, reducing the need for manual entry during a busy period in the patients' lives. This also facilitates telemonitoring by physicians who can review and advise on readings [11-13]. Secondly, does improved post-partum blood pressure control also result in reduced end organ damage in the cardiac, vascular and cerebrovascular systems? Hypertensive pregnancies are associated with early changes in cardiac, vascular and brain structure and function, which are disproportionate to their cardiovascular risk profile [14-16]. This may explain why this population have an increased risk of later cardiovascular and cerebrovascular disease. It is possible these changes may emerge during pregnancy and persist long-term, independent of post-partum blood pressure variability. Although the significant cardiovascular adaptations that emerge during pregnancies complicated by hypertensive

For peer review only

disorders of pregnancy are known to reverse to some extent during the post-partum period [17-20], an alternative hypothesis it that the long-term risk reflects a failure of the cardiac, vascular or cerebral systems to 'normalise' after pregnancy [21, 22]. If so, improved post-partum blood pressure control may offer a new approach to modify these long-term end organ changes, in addition to any beneficial effects on blood pressure.

HYPOTHESES

The primary hypothesis is that blood pressure self-management with clinician oversight will reduce diastolic blood pressure at six to nine months post-partum; in women requiring anti-hypertensive medication in the puerperium after a hypertensive pregnancy.
The secondary hypotheses are that blood pressure lowering in the puerperium and strict control within predefined target ranges during this time period will improve cardiovascular, and cerebrovascular and vascular phenotypes in this cohort including:

- MRI indices of cardiovascular structure & function,
- MRI indices of cerebral perfusion, white matter integrity, subcortical volumes,
- MRI assessment of aortic compliance,
- Echo measures of cardiovascular structure and function, especially diastolic function and left atrial volume.
- Improved cardiovascular adaptation to exercise, assessed by exercise ejection fraction during cardiopulmonary exercise testing (CPET).
  In addition, it is hypothesised the intervention will lead to improvements in peripheral vascular function, including integrity of the retinal vasculature and markers of vascular stiffness. The tertiary hypotheses are that self-management will be associated with higher quality of life scores once discharged from hospital, fewer post-natal readmissions to hospital, improved endothelial function; and fewer signs of kidney injury and fibro-inflammation.

METHODS

Planned Trial Period: 31/12/19-01/12/30
Planned Recruitment period: 31/12/19- 31/08/21

Study design

POP-HT is a single centre, open label, two-arm parallel, randomised controlled trial in women who develop hypertensive disorders of pregnancy, who require anti-hypertensive treatment at the time of discharge. This study will investigate the effectiveness of post-partum physician assisted self-management of blood pressure compared to standard care over the first six months post-partum.
We will recruit 200 participants, who will be randomly allocated in a 1:1 fashion to either the intervention or control arm. The intervention arm will comprise app-based home blood pressure monitoring (including periods of home 24hr ABPM) coupled with physician-assisted self-management. The control arm will receive 'standard' levels of NHS care from their GP and

For peer review only

midwives and health visitors. All participants will be recruited from the Oxford Women's Centre at the John Radcliffe Hospital, which sees approximately 25 patients with hypertensive disorders of pregnancy per month. A trial flow chart is presented in appendix A and a schedule of procedures in appendix B. The expected duration of participant involvement will be up to a maximum of 12 months.

Endothelial Cell Sub-study

The purpose of the sub study of 20 women is to provide a reference population of women not affected by hypertensive disease. In the sub study, 20 healthy postnatal women will undergo measurements of specific characteristics of blood cells and circulating factors involved in inflammation and endothelial dysfunction. This small sub-cohort population will validate how blood cells and circulating factors vary naturally and may be affected by external factors such as mode of delivery and blood pressure. These 20 normotensive participants will be compared to 20/200 participants who have had a hypertensive pregnancy and that are in the main study. These 20/200 will provide additional consent for blood sampling for endothelial cells at baseline and at the V4 visit. The 20 normotensive patients will be recruited from the postnatal ward in the Oxford Women's Centre and the normotensive participants will be recruited directly to the sub-study and, not be expected to participate in the main POP-HT study. (See appendix C for a schedule of procedures for the sub-study).

Study intervention

The intervention is the provision of a wireless Bluetooth® enabled OMRON Evolv® blood pressure monitor, validated for widespread clinical use, including in pregnancy [23] , alongside the installation of a proprietary smartphone app. The app will assist the self-management of post-partum blood pressure, with physician oversight of medication adjustment. Following the baseline visit, those randomised to the intervention arm will be provided with an OMRON Evolv® monitor and the app installed on the participant's phone and its use demonstrated. The participants will have ample time to become familiar with the device and app prior to discharge, as in SNAP-HT [4]. They will also be provided with an 'intervention-arm information sheet,' which contains information about the self-monitoring process as well as a frequently asked questions (FAQ) section and contact number for any technical issues. The system was developed by the Oxford Institute of Biomedical engineering (IBME) who have successfully developed apps for several other blood pressure studies including SNAP-HT [7], TASMIN [24] and BUMP (NCT03334149). Participants in the intervention arm will be asked to start home blood pressure readings on the day of discharge. Figure 1 illustrates the method of blood pressure self-management.

Following discharge from hospital, participants will be asked to upload their home blood pressure readings in a standardised manner. These readings are in turn uploaded to the secure NHS hosted web-based platform. The readings are automatically cross-checked against pre-defined algorithms and an appropriate notification will be generated in response.

The medications prescribed to each participant at discharge will be decided by their clinical care team. The medication schedule will be uploaded to a secure NHS hosted web-based platform which syncs automatically with the app. This approach was trialled successfully in the SNAP-HT pilot trial [7] and this study will be using the same approach to develop these dose titration schedules based on discharge medication. Medication titration will be done in line with the updated NICE guidance NG133 in the intervention arm (and for those in the control arm clinicians

For peer review only - http://bmjopen.bmj.com/site/about/guidelines.xhtml

are anticipated to also follow this new NICE guideline) [11]. Down-titration is triggered when BP is consistently <130 mmHg and <80 mmHg diastolic and the process for this is explained in further detail in Appendix D.

Patient and public involvement (PPI)

The study team hosted regular PPI meetings prior to the study commencing to understand the experiences of patients participating in prior self-management and pre-eclampsia studies, and assist with study design and methodology. This included consulting participants and investigators from other related studies conducted by this group. Participant-facing information documents, were reviewed by PPI members with experience of raised blood pressure in pregnancy. PPI members also helped design the intervention to ensure it was acceptable for participants. The PPI group will also assist in the drafting of study results for dissemination to participants.

Study aims and objectives

For clarity; the timepoint of the primary and blood pressure outcome is 6-9 months post-partum, whereas an extra 3 months (6-12 months) was approved by the trial steering committee, REC and HRA for completion of the other secondary outcome data collection (as part of an amendment to mitigate against the impact of the COVID-19 pandemic on follow up rates for these additional measures). This explains the different time points in table 1 below:

For peer review only

Date and version No: V 1.0 12/10/2021

Table 1

	Objectives	Outcome Measures	Timepoint(s)
**Primary**	To compare post-partum diastolic BP in the intervention arm vs. the control arm.	24 hour average diastolic BP measured by assessed by SPACELAB 90217 24hr Ambulatory blood pressure monitoring (ABPM)	Visit 4 (6-9 months post-partum)
**Secondary**	To compare the effect of the intervention on cardiovascular, cerebrovascular and vascular phenotypes	**BP based**<br>a) 24 hr average systolic blood pressure assessed by SPACELAB 90217 24hr ABPM<br>b) Mean diurnal diastolic blood pressure assessed by SPACELAB 90217 ABPM<br>c) Mean diurnal systolic blood pressure assessed by SPACELAB 90217 ABPM<br>d) Mean nocturnal diastolic blood pressure assessed by SPACELAB 90217 24hr ABPM<br>e) Mean nocturnal systolic blood pressure assessed by SPACELAB 90217 24hr ABPM<br>f) Mean bedside diastolic blood pressure measured during study visit (mean of 2+3)<br>g) Mean bedside systolic blood pressure measured during study visit (mean of 2+3)<br><br>**Cardiac MRI**<br>h) Left ventricular (LV) mass indexed to end-diastolic volume and body surface area (BSA)<br>i) LV EDV indexed to BSA<br>j) LV wall thickness – septum, posterior and RWT<br>k) LA volume indexed to BSA<br>l) Right ventricular (RV) mass indexed to end-diastolic volume and body surface area (MRI)<br>m) RV EDV indexed to BSA<br>n) RA volume indexed to BSA<br>o) LV ejection fraction (EF) & RV EF<br>p) LV and RV stroke volumes indexed to BSA<br>q) Myocardial fibrosis<br>r) ECV (extra-cellular volume)<br><br>**Echo**<br>s) LV Diastolic function: E/E' average, E/A ratio, E deceleration time<br>t) Global longitudinal strain (GLS)<br>u) LV systolic function (EF by Biplane Simpson's)<br>v) LA volume by Biplanar assessment<br><br>**Vascular**:<br>w) Pulse wave velocity<br>x) Augmentation index<br>y) Aortic BP<br>z) Aortic distensibility (MRI)<br><br>**Cerebrovascular**<br>aa) Total white matter hyperintensity volume<br>bb) Cerebral blood flow<br>cc) Mean vessel thickness of the middle and posterior cerebral arteries and internal carotid artery	6-9 months for the 24 hr ABPM measures<br><br>Baseline, week 1, week 6 and 6-9 months for the bedside blood pressures measures<br><br>For Cardiac MRI at 6-12 months post-partum<br><br>At baseline and at 6-12 months post-partum for Echo outcome measures<br><br>PWV, Aortic BP & AI at baseline & at 6-12 months (aortic stiffness);<br>Aortic compliance (on MRI) at 6-12 months<br><br>6 -12 months post-partum for all Brain MRI measures.

For peer review only

		**Retinal**<br>dd) the corrected central retinal arteriolar equivalent<br>ee) the corrected central retinal venular equivalent<br>ff) corrected central retinal arteriolar equivalent/corrected central retinal venular equivalent ratio.	6-12 months post-partum for all retinal measures
**Tertiary:**	To explore in-vitro vascular function in a sub-study of 20 women<br><br>To explore presence/absence of kidney injury and fibro-inflammatory status<br><br>Quality of life assessment<br><br>Participant experience: assessment of individual experience following intervention<br><br>Number of readmissions in intervention vs control arm<br><br>Side-effect impact	**Exercise Echo**<br>Exercise ejection fraction (echo) & exercise LA volume at 50% of peak workload during a bicycle cardio-pulmonary exercise test (CPET)<br>**CPET**<br>VO2 at VT1<br><br>Assessment of endothelial cell function and circulating biomarker levels associated with vascular angiogenesis and inflammation in normotensive and hypertensive women to determine if BP improvement can affect vascular function<br><br>T1 mapping of the kidneys to look at cortico-medullary differentiation<br><br>EQ-5D-5L health questionnaire results<br><br>Qualitative semi-structured interviews in a subset of individuals as well as assessment of acceptability and feasibility within the intervention arm<br><br>Readmission number in each arm<br><br>Number and frequency of side-effects reported (intervention via the app and control during follow up calls/SMS)	6-12 months post-partum<br><br>6-12 month post-partum<br><br>From baseline to 6-12 months post-partum<br><br>6-12 months post-partum<br><br>Baseline, week 1, week 6 and 6-12 months post-partum<br><br>6-12 months post-partum<br><br>0-12 months post-partum<br><br>0-12 months post-partum
Intervention(s)	The intervention will consist of physician-optimised self-management of post-partum BP. Women will follow a 'smartphone' app-based algorithm for medication titration, which will provide individualised dose titration advice. This is overseen and any change is approved by physicians who review the uploaded readings and respond to tele-monitored abnormal readings in a timely fashion.		
Comparator	The control arm will be managed as per usual NHS-led care with assessment by their own health care professionals and adjustment of medications as required. The BP of this group will be monitored and recorded at the same time-points and in the same manner as the intervention arm as with all other secondary outcome measures.		

For peer review only

Eligibility and recruitment

The procedures for each study visit and the estimated time each will take are listed in appendices B and C respectively.

All trial participants will be recruited locally from the Women's Centre at the John Radcliffe Hospital, Oxford. Screening will be carried out by the patient's clinical care team. Consent will be performed by the research team, once verbal consent is given to the clinical team for them to be approached.

Main Trial Participants

All participants will be females of childbearing age >18 years of age. Entry into the trial will require a clinician confirmed diagnosis of either gestational hypertension or pre-eclampsia defined by NICE NG 133 [11], that requires anti-hypertensive medication.

Inclusion Criteria

- Participant is willing and able to give informed consent for participation in the trial.
- Female, aged 18 years or above.
- Clinician confirmed diagnosis of either gestational hypertension or pre-eclampsia defined by NICE NG 133 and remains in hospital after delivery.
- Requiring anti-hypertensive medication at the point of discharge from secondary care.
- Participant has clinically acceptable laboratory results and clinical course post-partum with no other adverse complicating factor requiring prolonged admission post-partum that would make participation unfeasible as judged by the CI. Examples would include stroke sequalae, ongoing DIC, or other significant life-threatening co-morbidity
- In the Investigator's opinion, is able and willing to comply with all trial requirements including ownership of a smartphone or tablet and willing to use the smart-phone app if randomised to that arm.
- Sufficient competence in English language to follow the app instructions and partake in the study, as judged by the CI.

Exclusion Criteria (the participant may not enter the trial if ANY of the following apply):

- Significant renal or hepatic impairment that would affect safe medication titration and adjustment as part of the trial, as deemed by the Investigator.
- Participant with life expectancy of less than 6 months.
- Any other significant disease or disorder which, in the opinion of the Investigator, may either put the participant at risk through participation in the trial, influence the result of the trial, or impair the participant's ability to participate in the trial.
- Participants who have participated in another research trial involving an investigational product in the past 12 weeks.
- Women with pre-existing hypertension will be excluded, as this is a separate pathology that would affect the efficacy of the study intervention and affect the primary and secondary outcomes of the study.

ENDOTHELIAL CELLS SUB-STUDY

For peer review only

Dysregulation of the vascular endothelium and endothelial cells has been observed in the pathogenesis and progression of several cardiovascular diseases, including hypertension and hypertensive pregnancy disorders [25-31]. Studies have demonstrated that significant peripartum inflammation, endothelial dysfunction and angiogenic imbalance extends beyond delivery, and persists to 5-10 years post-partum [32-34]. Endothelial colony-forming cells (ECFCs) represent a highly proliferative subtype of endothelial progenitor cells (EPCs), which play a vital role in the regulation of vascular homeostasis [35-37]. Herein, we will investigate endothelial cell function using peripheral blood derived-ECFCs and angiogenic biomarkers in the blood at baseline and at the final visit (V4), which takes places approximately 6 months post-partum

The inclusion criteria for the blood validation sub-study include:

- Participant is willing and able to give informed consent for participation in the trial.
- Female, aged 18 years or above.
- Normotensive (BP <140/90mmHg) throughout antenatal and postnatal period (except the 20/200 recruited from the main study)

The exclusion Criteria for POP-HT blood validation sub-study include:

- A hypertensive disorder of pregnancy (for the 20 normotensive recruits required)
- Use of beta blockers such as atenolol or equivalent
- BMI>35
- Evidence of cardiomyopathy, inherited cardiac conduction abnormalities, congenital heart disease or significant chronic disease relevant to cardiovascular status
- Folic acid or folate supplementation in the third trimester

Randomisation

Randomisation will be performed as soon as possible following the baseline visit. Randomisation will be carried out using a secure web-based randomisation software (embedded within Castor®). Participants will be randomised on a 1:1 basis and two minimisation factors will be used to ensure that the groups are matched as well as possible:

- Primary factor: Gestational age at the time of presentation with pre-eclampsia/gestational hypertension (agreed on as a surrogate marker of disease severity).
- Secondary factor: Prescription of ACE inhibitor (Enalapril) at the time of randomisation.

The full trial protocol (available on request) details more information on allocation concealment and implementation of the randomisation.

Assessments during study visits

A flow chart of the proposed study visits is included below (figure 2). All data will be recorded directly into CASTOR® electronic data capture forms where possible in real-time. Any data requiring post-processing will be entered into CASTOR following such analysis. During the study visits, all procedures are performed on participants in both the intervention and control arms with the exception of provision of the intervention; which is reserved only for those randomised to that arm. At all study visits involving a review of the medical and obstetric history, the number

For peer review only

and dose(s) of anti-hypertensive medication is recorded to allow comparison between groups post-hoc.

As figure 2 illustrates, there will be 'essential' components to each study visit. However, as the research is being carried out on women with newborn babies, some components have been classed as 'desirable' to allow for shortening of the study visits, if required, without affecting the primary outcome. A number of modifications to the original study design, and the means of performing the above assessments, have also been made to mitigate the impact of the COVID-19 global pandemic (please see appendix E for further detail in the supplementary file). All amendments have been submitted to; and approved by SPONSOR, the REC and HRA, the local hospital (OUH) trial management authority; and other relevant parties have been notified where needed (see Appendix F for amendment history).

<u>Baseline visit (week 0):</u>

Demographics and anthropometry

Assessment will include recording of the ante-natal booking height, weight and BMI (obtained from notes) and the mid-left arm circumference.

Bed-side Blood pressure measurement

Participants will have their blood pressure checked after 5 minutes' rest using the automated mode of a validated sphygmomanometer. Three blood pressure readings will be taken at intervals of 1 minute. The measurement technique advised by the British Heart Foundation and NICE NG133 [11] will be strictly followed.

Echocardiogram (cardiac ultrasound) scan

Cardiac ultrasound imaging will be performed by a trained sonographer to evaluate cardiac structure and function. British Society of Echocardiography guidelines will be followed for collection of a standard clinical imaging dataset

Collate data from medical notes and review blood results

A study team member will review the medical notes (paper and electronic) to document relevant medical history as stated in the full trial protocol

Quality of Life questionnaire (EQ-5D-5L)

Participants will be provided with an EQ-5D-5L questionnaire via e-mail

Desirable: Vicorder® (Vascular Measures and Central Blood Pressures)

Resting measures of vascular stiffness including pulse wave velocity and central blood pressure will be collected using a non-invasive device (Vicorder®).

Desirable: Lifestyle and diet questionnaire

The questionnaire, sent via e-mail, combines validated questions piloted or used in previous studies. Information will be collected on factors that affect blood pressure including: smoking frequency, alcohol and salt intake, exercise and family history.

For peer review only

Intervention provision: Automated blood pressure cuff provided and POP-HT app installed (those randomised to intervention arm only)

At the end of the baseline visit, those individuals that are randomised to the intervention arm will be issued with an OMRON EVOLV® automated blood pressure cuff (validated for use in pregnancy [23]). They will also download the POP-HT smartphone app and their use will be demonstrated. The participant will then have the remainder of their stay in hospital to practice. This is to ensure all parties are confident and competent prior to discharge home, at which point the intervention will start. Participants will be able to contact the study team via telephone or email for any technical problems.

Control arm during COVID-19: During the COVID-19 pandemic, the RCOG recommends 'self-monitoring 2-3 times in the first week after discharge' for women who have had a hypertensive pregnancy. Therefore, those women allocated to the control arm, who are unable to obtain a monitor from the Oxford Women's centre/NHS service, will be provided with a validated BP home-monitoring device by the trial team to ensure they can adhere to this RCOG guidance during week one. These monitors will be provided to enable the control arm participants to monitor their own blood pressure and in turn liaise with their own GP/mid-wife to adjust their management based on their readings. The study team will not be offering remote management to the control arm or providing them with an app, interpretation of the readings and management decisions are to be taken by their own GPs/clinicians. These monitors will also be used to allow remote BP measurements during the study visits at weeks one and six.

Subsequent Visits

- During the week one and six follow-up, the research team will perform all procedures for participants in both arms. As a result of the COVID-19 pandemic, all aspects of these follow-ups will be conducted remotely via video (and/or phone call).

Visits 2 and 3

Weeks 1 and 6 (± 5 days) post-discharge: blood pressure will be measured as per the baseline visits, up-to-date anthropometry will be measured, and an ED-5D-5L questionnaire will be completed.

Visit 3 will also involve an ambulatory blood pressure monitor (to be worn for a 24-hour period)

24-hour ambulatory blood pressure monitoring will be initiated at the end of the study visit using a validated, calibrated, automated oscillometric, ambulatory devices (SPACELABS® 90217 or equivalent). Correct cuff size will be chosen based on arm circumference recorded at week 1 and 6.

Visit 4 (6 months (up to 12 months) post-partum

Participants will be invited to this final study visit. This is a more comprehensive visit with both essential and desirable components. A female chaperone will be offered and, where possible, all echocardiography will be performed by a female sonographer. In extenuating circumstances, such as COVID-19 national lockdowns, the 24hr BP monitoring and the procedures below can be conducted remotely via video call. Otherwise, these will be performed in person as described for the baseline visit.

- **Demographics and anthropometry:** Assessment will be performed as outlined above
- **Blood pressure:** Assessment will be performed as outlined above
- **Quality of Life questionnaire (EQ-5D-5L)**

For peer review only

- **Fitting of a 24 hour blood pressure monitor:** Assessment will be performed as outlined above
- **Fitting of an activity monitor:** For remote video calls, the accelerometer will be pre-programmed based on participant reported height, weight and hand dominance and then posted to the participant. For visits carried out in person, the accelerometer will be programmed during the visit, based on their height and weight recorded as part of the study visit and, fitted to their non-dominant wrist.
- **Review of medical and obstetric history and any medication side effects:** as described above

In cases where the above measures are performed remotely the additional procedures below, which cannot be done at remotely will be scheduled as soon as possible after, and within the 12-month time window defined in the protocol.

Echocardiogram cardiac ultrasound scan: will be performed as outlined above for baseline visit.
Vicorder® (Vascular Measures and Central Blood Pressure) assessment: will be performed as outlined above, but whilst in the MRI scanner, in order that the central/aortic pressure can be correlated with the aortic distensibility images obtained during the MRI scan.

Retinal imaging

Retinal photography of the right eye (3 single shot images centred on the optic disc) will be completed using a digital camera and imaging software following an established protocol.

MRI

A 3Tesla (3T) Siemens PRISMA scanner will be used to quantify brain structure and volume, followed by cardiac structure and function, cardiac mapping, measurement of aortic distensibility; and T1 maps of the kidneys.

Gadolinium contrast (optional)*:* Gadolinium will be offered as an additional optional component to the MRI to those women who are not breast feeding, as part of exploratory work that may feed into a larger future trial. The additional PIS explains the Gadolinium procedure and risks/benefits in more details. Separate informed consent will be obtained for those women who wish to participate in this aspect of the study prior to the MRI being performed.

Blood: A venous blood sample (approximately 25mls) will be taken at rest and include samples for a) whole blood, plasma and serum lipid and inflammatory marker analysis and b) analysis of biochemistry and metabolism.

Cardiopulmonary Exercise Testing with exercise echo: Desirable

Cardiac function and oxygen requirements in response to an incremental increase in workload will be measured via a cardiopulmonary exercise test (CPET). The exercise protocol is a validated incremental protocol with established use in clinical and research practice. The exercise protocol is currently utilised in ongoing ethically approved studies conducted by the Division of Cardiovascular Medicine, and is performed on a stationary bike. The test commences with resting measures of spirometry. Participants will then exercise with an incrementally increasing workload up to 40-60% of their estimated peak exercise capacity. A brief focused echo will be performed at rest and at 40% of their maximal predicted exercise intensity (whilst on the bike) to enable measurement of exercise ejection fraction.

Sub- study Visits: Circulating biomarker validation and evaluation

For peer review only

20 of the hypertensive pregnancy patients from the main trial will also have a blood test taken at baseline as part of their main study visit. For the normotensive participants, the following study procedures and visits listed below remain separate to the main study. Study procedures will be the same during both visits.
The baseline visit will be carried out on the postnatal ward, in the Women's Centre, prior to discharge. Normotensive participants will be invited back for a second visit at the John Radcliffe Hospital when they reach 6 months postpartum, and for those 20 hypertensive participants taking part in the main trial, the repeat blood test for the sub-study will be performed during the main V4/final visit when they have other study blood tests performed.

Procedures for all sub-study patients include: Demographics and anthropometry, Bed-side Blood pressure measurement and a blood test for analysis of biomarkers associated with inflammation, angiogenesis and endothelial activation as well as endothelial colony forming cells (ECFCs). Blood tests will be taken, where possible, at the same time as clinically-indicated venepuncture.

Further details and explanation of the assessments is contained in appendix G: 'Copy of the PIS'

Data Analysis

Power calculations to determine adequate sample sizes for this trial are summarised in table 2 below.

Table 2

**Primary outcome measure: 24-hour average diastolic blood pressure (mmHg) at 6-9 months post-partum as assessed by SPACELAB 90217 24hr Ambulatory blood pressure monitor**<br>**Sample size calculation**: The detection of BP differences between the 2 arms of this trial is based on the mean diastolic blood pressure difference detected in the pilot SNAP-HT study at 6 months. The mean BP difference detected between the intervention and control arm at the 6 month time-point was -4.5mmHg [7]. We have used a more conservative standard deviation (SD) of 10mmHg in each arm (in SNAP-HT the SD was 8.2mmHg in the intervention arm and 9.8 mmHg in the standard care arm) and a 10mmHg SD is in keeping with pooled SDs for ambulatory diastolic blood pressure readings from other studies. To detect a treatment effect on diastolic blood pressure of -4.5mmHg, powered to 80% at p=0.05 requires a total sample size of 158 and with 1:1 randomisation this would require 79 in each arm (total sample size of 158). During COVID-19 a Royal College of Obstetricians and Gynaecology (RCOG) guideline [38] was issued that recommended a home BP monitor be given to all women for the first week(s) after discharge. As a result of the potential dilution that self-monitoring in the control group could have, we re-calculated our sample size. One systematic review [39] concluded self-monitoring in the control arm could lead to a potential 0.42mmHg dilution of the impact of self-management on diastolic BP, when measured using 24hr ABPM. Therefore, assuming the same SDs of 10mmHg in each arm as in our original power calculation, we subtracted 0.42mmHg from the 4.5mmHg between group difference we had originally powered on. To remain powered at > 80% and we would require 95 in each group (190 total) and hence we planned to over-recruit to 220 (rather than the original 200) to allow for this.
**Secondary outcome hypothesis: Improved blood pressure control in the post-partum period in POP-HT will result in improved cardiac, vascular and cerebrovascular phenotypes at 6-12 months post-partum**
**Secondary outcome power calculations**<br>**a)** **Cardiac structure***:* Studies using echocardiography by our collaborators have compared BP and LV mass in pre-eclampsia patients and control patients, at 1 year post-partum[16, 40][16, 40][16, 39][16, 38][16, 38][16, 38][16,

38][16, 38][16, 38][15, 37][14, 36][14, 37][14, 37][14, 36][14, 35] [14-15; 26, 36]. They found that a difference in BP at 1 year of 10mmHg in diastolic BP corresponded to significant differences in LV mass. SNAP-HT appeared to achieve a 50% reduction of anticipated BP difference seen between pre-eclamptic and normotensives at 1 year by 6 months i.e. ~5mmHg. If it is assumed that the structural/phenotypic benefit results from the BP benefit, as we are hypothesising, then we must power to detect 50% of the phenotypic difference. In previous work by our group we have demonstrated significant differences in LV mass/EDV (g/ml) in a similar age and predominantly female population with similar mean diastolic BP differences between groups to that seen in SNAP-HT. The LV mass/EDV (g/ml) in the group with high normal blood pressure was 1.54g/ml vs. 1.22 g/ml in those with optimal blood pressure with a standard deviation of 0.33 and 0.27 respectively at P<0.001. Based on these assumptions, to observe a treatment effect of 0.16 (50% of the difference between 1.54g/ml and 1.22g/ml) on LV mass/EDV, requires 67 in the intervention arm and 67 in the control arm (132 total). This is calculated using the larger SD of 0.33 referenced above at a power of >80% to detect a difference between the groups at p=0.05. This number should take into account for the greater dropout rate we may see for the MRI outcomes.

b) **Brain White matter integrity***:* Work by our group, on pre-eclamptic pregnancy, showed an increased burden of temporal lobe white matter lesion volume 5-10 years after a pre-eclamptic pregnancy (23.2±13 µl) vs matched individuals who had a normotensive pregnancy (10.9 ± 11.5 µl) at p<0.05.If we again assume we can detect a 50% of the phenotypic benefit with our intervention as outlined above, we would anticipate a 50% reduction in the burden of white matter lesions i.e. 6.15 µl (50% of 23.2 – 10.9 µl) in the intervention arm. With 71 in the intervention group and 71 in the control group (142 total), this will provide >80% power at p=0.05, even using the more conservative SD of 13ul to detect a 50% improvement in white matter lesion volume between the intervention and the control group. This number should take into account for the greater dropout rate we may see for the MRI outcomes.

c) **Aortic compliance**

Several studies assessing the impact of blood pressure on aortic compliance have shown that even modest reductions in systolic/diastolic blood pressure increase aortic distensibility/compliance. One such study had a mean difference in systolic blood pressure of 4.6mmHg between the 2 drug treatment arms at 52 weeks, akin to the same mean difference in SNAP-HT at 6 months, albeit this was diastolic not systolic, although other studies have suggested diastolic BP may be even more important in influencing aortic compliance. In this study with a mean 4.6mmHg difference in systolic BP there was a treatment difference of 0.12 [(95% CI −0.35, 0.60), *P* = 0.60 in aortic compliance. Based on these assumptions, to observe a treatment effect from our intervention, with 100 in the intervention and 100 in the control arm we will be more than powered at >90% to detect a difference at P=0.05 in POP-HT. **In this study we will assess both aortic stiffness (by Vicorder PWV and AI) and aortic compliance by MRI although power calculation here is based on MRI measures of aortic compliance**

Exercise ejection fraction

Within our group, Huckstep et al compared resting and exercise ejection fractions for young adults with high normal BP vs. a normotensive cohort[41]. The cohort was well matched demographically to our planned study cohort, albeit it included both males and females. Resting ejection fraction (by Biplane Simpson's) was similar between groups but at 40%-60% of peak exercise intensity, the higher blood pressure group had a lower exercise ejection fraction than the normotensive cohort (73.9±3.25 vs. 80.0±4.54%, p<0.001) and in keeping with this, a smaller increase in ejection fraction when going from baseline to 40% exercise intensity (10.4±5.92 vs. 19.0±6.90%, p<0.001). Assuming the ~5mmHg mean BP improvement achieved in SNAP-HT again translates to a 50% phenotypic benefit, when assessing exercise ejection fraction we anticipate a 4.3% improvement in exercise stress ejection fraction in the intervention arm vs. the control arm (4.3% is 50% of the difference i.e. 50% of 10.4-19%). With 43 participants in the intervention arm and 43 in the control arm (86 total) we will be powered at >80% to detect such a difference at p=0.05. This calculation also takes account of the lower number likely to undertake the CPET at the final study visit.

Statistical Analysis Plan (SAP)

The statistical aspects of the study are summarised here with details fully described in a statistical analysis plan that will be produced in due course.

For peer review only

Description of Statistical Methods

The analysis will be carried out on the basis of intention-to-treat (ITT). This is, after randomisation, participants will be analysed according to their allocated intervention group irrespective of what treatment they actually receive. Patient demographic characteristics and other baseline information will be summarised by treatment group. Numbers (with percentages) for binary and categorical variables and mean (standard deviation), or median (interquartile or full range) for continuous variables will be presented.
A linear mixed model will be applied to compare the groups with respect to the primary outcome. The model will include baseline bedside (i.e. clinic) diastolic BP (mean of the 2nd and 3rd bedside diastolic blood pressures, randomised group and minimisation factors (gestational age at the time of presentation with pre-eclampsia/gestational hypertension (continuous) and prescription of ACE inhibitor at randomisation) as fixed effects. Participant will be included as a random effect. For all participants included in the primary outcome analysis, the mean 24 hour average diastolic blood pressure will be reported by randomised group. Adjusted mean differences between randomised groups with 95% confidence interval and p value will be estimated from the model for the following comparison: self-management (intervention) versus usual care (control) at a single time point (V4: the final study visit).

Secondary blood pressure outcomes will be analysed using the same method. Other secondary outcomes will be analysed using analysis of covariance (ANCOVA) to establish a co-variant model to examine the effect of blood pressure control in the post-partum period on cardiac structure and function, vascular function and cerebrovascular structure and function. If the model assumptions are not met and evidence of departure from normality is observed, transformations of the data will be employed or non-parametric tests will be carried out. Descriptive statistics (mean, standard deviation, standard error, range, etc.) will also be calculated for each outcome for each group. Differences in the secondary outcomes will be compared between intervention and control groups.
Mean changes in blood pressures will be compared across the population and correlated with cardiovascular endpoints including cardiac structure and function reported from cardiac MRI and echocardiogram. Demographic and physiological characteristics of the participants will be added to regression models as covariates to explore the determinants of change in blood pressure comparing intervention and control groups.

Analysis Populations

The participants that will be included in the analysis will be all of those randomised. All data will be included in the analysis as far as possible to allow full ITT analysis, though there will inevitably be the problem of missing data due to withdrawal, loss to follow-up or non-completion of questionnaire data.

Decision Points

For peer review only

There will be no formal interim analysis. The results once analysed will be reviewed by the research team, the Trial Steering Committee (TSC) and Data and Safety Monitoring Committee (DSMC) and the PI/CI and other collaborators.

The Level of Statistical Significance

Level of significance will be tested as a 5% two-sided significant level.

Procedure for Accounting for Missing, Unused, and Spurious Data

Missing data: Missing data will be reported with reasons given where available and the missing data pattern will be examined. We will explore the mechanism of missing data, though the mixed effects model implicitly accounts for data missing at random. The need for a sensitivity analysis taking into account missing data using multiple imputation will be considered and outlined further in the SAP. Spurious data will be assessed using standard editing criteria.

Procedures for Reporting any Deviation(s) from the Original Statistical Analysis Plan (SAP)

The final statistical plan will be agreed prior to final data lock and prior to any analyses taking place. Any deviation thereafter will be reported in the final trial report.

TRIAL OVERSIGHT

The trial will be conducted in accordance with the current approved protocol, GCP, relevant regulations and standard operating procedures. A risk assessment and monitoring plan are not being prepared before the study opens, as it is a low risk intervention.

Trial committees

A trial steering committee (TSC) will convene prior to the study starting and half-yearly thereafter to review and address key aspects of the study including the following:

1. Recruitment
2. Safety/adverse event
3. Withdrawals
4. Data management
5. Statistical analysis plan

The TSC will also function as a data safety and monitoring committee (DSMC) for this particular study and there will be a smaller trial management committee as outlined in the study synopsis, which will focus more on the week-to-week running of the trial and will be on a more regular basis.

Monitoring

Direct access will be granted to authorised representatives from the Sponsor within the appropriate department and host institution for monitoring and/or audit of the study to ensure compliance with

For peer review only

regulations. Following written standard operating procedures, the monitoring visits will verify that the clinical trial is conducted and data are generated, documented and reported in compliance with the protocol, GCP and the applicable regulatory requirements.

SAFETY REPORTING

Table 3: Adverse Event Definitions

Serious Adverse Event (SAE)	A serious adverse event is any untoward medical occurrence that:<br>• results in death<br>• is life-threatening<br>• requires inpatient hospitalisation or prolongation of existing hospitalisation<br>• results in persistent or significant disability/incapacity<br>• consists of a congenital anomaly or birth defect.<br>Other 'important medical events' may also be considered a serious adverse event when, based upon appropriate medical judgement, the event may jeopardise the participant and may require medical or surgical intervention to prevent one of the outcomes listed above.<br>NOTE the term "life-threatening" in the definition of "serious" refers to an event in which the participant was at risk of death at the time of the event; it does not refer to an event, which hypothetically might have caused death if it were more severe.

NB: to avoid confusion or misunderstanding of the difference between the terms "serious" and "severe", the following note of clarification is provided: "Severe" is often used to describe intensity of a specific event, which may be of relatively minor medical significance. "Seriousness" is the regulatory definition supplied above.

Procedures for Reporting Adverse Events

A serious adverse event (SAE) occurring to a participant will be reported to the REC that gave a favourable opinion of the study where in the opinion of the Chief Investigator the event was 'related' (resulted from administration of any of the research procedures) and 'unexpected' in relation to those procedures. Reports of related and unexpected SAEs should be submitted within 15 working days of the Chief Investigator becoming aware of the event, using the HRA report of serious adverse event form (see HRA website). The severity of events will be assessed on the following scale: 1 = mild, 2 = moderate, 3 = severe.

Non-serious AEs considered related to the trial intervention as judged by a medically qualified investigator or the Sponsor will be followed up once the event is considered stable. It will be left to the Investigator's clinical judgment to decide whether or not an AE is of sufficient severity to require the participant's removal from the trial. A participant may also voluntarily withdraw from the trial due to what he or she perceives as an intolerable AE. If either of these occurs, the participant must undergo an end of trial assessment and be given appropriate care under medical supervision until symptoms cease, or the condition becomes stable.

For peer review only

Events exempt from immediate reporting as SAEs

There are a number of expected admissions/consultations with healthcare providers that will be expected take place as part of the natural history of pre-eclampsia and gestational hypertension during the trial period. These will be classed as 'Foreseeable Events' exempt from reporting as SAEs and include:

- Severe hypertension;
- Maternal morbidity: visual disturbance; pulmonary oedema; respiratory failure; myocardial ischaemia; hepatic dysfunction, hepatic haematoma or rupture; and acute kidney injury;
- Postpartum haemorrhage;
- Lower genital tract bleeding;
- Sepsis;
- Admission to hospital for pre-eclampsia, monitoring of hypertension, or symptoms of low blood pressure
- Pre-planned hospitalisation;
- Diagnostic and therapeutic procedures including blood transfusion;
- Worsening pruritis;
- A pre-existing maternal condition (such as renal disease), unless it causes increased clinical concern;
- Admission for psychiatric or social reasons;
- Retained placenta;
- Extended hospital stay of the mother due to the need to keep the baby/babies in hospital;
- Neonatal care unit admission for indications unrelated to pregnancy hypertension, such as neonatal hyperbilirubinaemia or unanticipated care for a fetal anomaly; or
- Fetal congenital anomaly

This list is not exhaustive and therefore any other 'minor medical significance symptom', as judged by the CI/PI, which does not require inpatient hospitalisation/prolongation of existing hospitalisation or result in persistent or significant disability/incapacity, and is not life-threatening and, does not result in death, will not be classed as an adverse event not an SAE.

DISCUSSION

Until recently, key evidence missing from trials of self-monitoring/tele-monitoring was whether it actually led to lower BP. In 2018, the TASMIN-H4 randomised trial [42] showed that GPs using self-monitored BP to titrate anti-hypertensives, with or without tele-monitoring, achieved better BP control for patients using tele-monitoring. As with previous trials, the mechanism of action appeared to be medication optimisation. More recent work shows that patient and clinician experience was largely positive and cost-effectiveness analysis suggests that self-monitoring in this context is cost-effective by NICE criteria [43]. Self-monitoring can be combined with self-titration of medication, a process known as self-management. The SNAP-HT trial [1] demonstrated that self-management postpartum following a hypertensive pregnancy offers great promise. The purpose of the POP-HT trial is to assess whether this BP reduction can be reproduced in a larger, randomised, single-blinded study powered to detect differences in BP as the primary outcome.

Our group have studied women 10 years after hypertensive and normotensive pregnancies to characterise their cardiovascular, vascular and cerebro-vascular systems. Consistent with previous reports [18, 19, 44], women who had been through hypertensive pregnancy were more likely to have LVH, increased LV mass and impaired diastolic function. In addition, cerebrovascular

For peer review only - http://bmjopen.bmj.com/site/about/guidelines.xhtml

changes were evident including; lower grey matter volumes, and greater white matter lesion density compared to the control population [14, 45]. Vascular phenotypic changes included reduced capillary density and increased aortic stiffness [15, 46]. These phenotypic differences were not explained by differences in traditional cardiovascular risk factors at the time of assessment. It is possible such differences emerge during pregnancy and persist independent of postpartum BP variability but this is not really known[47] and, although the cardiovascular adaptations that emerge during hypertensive pregnancies reverse to some extent postpartum [17-20], an alternative hypothesis is that long term 'risk' reflects a failure of the cardiac, vascular or cerebral systems to 'normalise' after pregnancy [21, 22, 47, 48]. In POP-HT, the trial aims to determine if these phenotypic changes emerge as early as 6-9 months and if so, whether they can be mitigated by postpartum blood pressure optimization. If so, this may offer one approach to modify long-term end organ changes, in addition to any beneficial effects on blood pressure that are demonstrated in this trial.

ETHICS AND DISSEMINATION

Declaration of Helsinki

The Investigator will ensure that this trial is conducted in accordance with the principles of the Declaration of Helsinki.

PUBLICATION POLICY

The Investigators will be involved in reviewing drafts of the manuscripts, abstracts, press releases and any other publications arising from the study. Authors will acknowledge that the study was funded by the British Heart Foundation Clinical Research Training Fellowship (BHF Grant number FS/19/7/34148). Authorship will be determined in accordance with the ICMJE guidelines and other contributors will be acknowledged. The summarised results will be published in a scientific journal/s and summarised on the CCRF website for participants to read. Should participants wish to have a copy of any papers published, they merely need to contact the study team, using the contact details provided on their PIS, and the team would be happy to provide one.

Guidelines for Good Clinical Practice

The Investigator will ensure that this trial is conducted in accordance with relevant regulations and with Good Clinical Practice. The trial protocol and all accompanying documentation has been approved by the Sponsor, an external REC, the HRA (Ethics Ref: 19/LO/1901; IRAS Project ID: 273353) and OUH (the local NHS trust) trial management authority (TMA).

Consent

The participant must personally sign and date the latest approved version of the Informed Consent form(s) before any trial specific procedures are performed. Please see appendix E for details of how consent will be modified during the COVID-19 pandemic to reduce risk of the paper acting as a vector for transmission of Coronavirus. Full details on the consent process are in the fully study protocol.

For peer review only

Participant Confidentiality

The study will comply with the General Data Protection Regulation (GDPR) and Data Protection Act 2018, which require data to be de-identified as soon as it is practical to do so. Further details are outlined in the study protocol and participant information sheets.

Access to Data

Direct access will be granted to authorised representatives from the Sponsor, host institution and the regulatory authorities to permit trial-related monitoring, audits and inspections. Further detail is provided in the fully study protocol. Data will be made available, under certain circumstances, following data lock upon request to the study PI.

Data Management

The study will comply with the General Data Protection Regulation (GDPR) and Data Protection Act 2018. The University of Oxford, as sponsor will act as data controller for the study.

FUNDING

The research is being financed by a British Heart Foundation Clinical Research Training Fellowship (BHF Grant number FS/19/7/34148).

AUTHORS'S CONTRIBUTIONS

JK, AJL, RM, KT, YK, CA, AF, AC, JM LM and PL contributed to the design of the study. JK, AJL and RM and PL secured funding. JK, AJL, RM, YK, AMC, AF, WW, KS, LM, CA, AC, MS, CR, KT, WL and PL refined the overall study protocol and lead the project delivery. BT and LC have provided guidance and external refinement. JK, MS and CR designed and oversee the delivery of the intervention. JK, AMC, YK, and WW will contribute to 24hr BP data acquisition and analysis. AJL, WL, JK and RM contributed to the development of the Brain and Cardiac MRI protocols. JK, RM, MK, WL and AJL will contribute to MRI image acquisition and quality control. WL will lead brain MRI image processing and analysis. JK will lead the cardiac MRI analysis with support from AJL, MK and WW. ET and BR helped develop the renal MRI sequences and will lead on renal MRI analysis. Echocardiography acquisition and analysis will be overseen by JK and performed by blinded study investigators. Cardiopulmonary exercise testing and peripheral cardiovascular risk assessment will be overseen by JK, AJL, WW, AMC and PL. HH will oversee retinal image acquisition and analysis. AF will run the sub-study on endothelial cells and, CT will oversee analysis of circulating biomarkers within the sub-study alongside AF.

INSURANCE

The University has a specialist insurance policy in place, which would operate in the event of any participant suffering harm because of their involvement in the research (Newline Underwriting Management Ltd, at Lloyd's of London). NHS indemnity operates in respect of the clinical treatment that is provided.

For peer review only

COMPETING INTERESTS

LM is supported by the NIHR Oxford Biomedical Research Centre and is a part-time employee of Sensyne Health PLC and holds shares in this company. RJM has received BP monitors for research from Omron and is working with them to develop a telemonitoring system. Any fees / consultancy from this work are paid to his institution.

APPENDICES/SUPPLEMENTARY MATERIAL

See supplementary file for appendices referenced in the text

For peer review only

REFERENCES

1. Ananth, C.V., K.M. Keyes, and R.J. Wapner, *Pre-eclampsia rates in the United States, 1980-2010: age-period-cohort analysis.* BMJ, 2013. **347**: p. f6564.
2. Walters, B.N. and T. Walters, *Hypertension in the puerperium.* Lancet, 1987. **2**(8554): p. 330.
3. McDonald, S.D., et al., *Cardiovascular sequelae of preeclampsia/eclampsia: a systematic review and meta-analyses.* Am Heart J, 2008. **156**(5): p. 918-30.
4. Ray, J.G., et al., *Cardiovascular health after maternal placental syndromes (CHAMPS): population-based retrospective cohort study.* Lancet, 2005. **366**(9499): p. 1797-803.
5. Behrens, I., et al., *Risk of post-pregnancy hypertension in women with a history of hypertensive disorders of pregnancy: nationwide cohort study.* BMJ, 2017. **358**: p. j3078.
6. Lazdam, M., et al., *PP032. Unique features of long-term cardiovascular phenotype in young women with early-onset pre-eclampsia.* Pregnancy Hypertens, 2012. **2**(3): p. 259-60.
7. Cairns, A.E., et al., *Self-Management of Postnatal Hypertension: The SNAP-HT Trial.* Hypertension (Dallas, Tex, 2018. **: 1979). 72**(2): p. 425-432.
8. Kitt, J.A., et al., *Short-Term Postpartum Blood Pressure Self-Management and Long-Term Blood Pressure Control: A Randomized Controlled Trial.* Hypertension, 2021. **78**(2): p. 469-479.
9. Luo, D., et al., *Association between high blood pressure and long term cardiovascular events in young adults: systematic review and meta-analysis.* BMJ, 2020. **370**: p. m3222.
10. Law, M.R., J.K. Morris, and N.J. Wald, *Use of blood pressure lowering drugs in the prevention of cardiovascular disease: meta-analysis of 147 randomised trials in the context of expectations from prospective epidemiological studies.* BMJ, 2009. **338**: p. b1665.
11. Excellence, N.I.f.C., *Hypertension in Prengnacy: diagnosis and management*, NICE, Editor. 2019: https://www.nice.org.uk/guidance/ng133.
12. Webb, A.J., et al., *Response of day-to-day home blood pressure variability by antihypertensive drug class after transient ischemic attack or nondisabling stroke.* Stroke, 2014. **45**(10): p. 2967-73.
13. Webb, A.J.S., et al., *Prognostic Significance of Blood Pressure Variability on Beat-to-Beat Monitoring After Transient Ischemic Attack and Stroke.* Stroke, 2018. **49**(1): p. 62-67.
14. Siepmann, T., et al., *Long-term cerebral white and gray matter changes after preeclampsia.* Neurology, 2017. **88**(13): p. 1256-1264.
15. Boardman, H., et al., *Comprehensive multi-modality assessment of regional and global arterial structure and function in adults born preterm.* Hypertens Res, 2016. **39**(1): p. 39-45.
16. Melchiorre, K., et al., *Maternal cardiac dysfunction and remodeling in women with preeclampsia at term.* Hypertension, 2011. **57**(1): p. 85-93.
17. Melchiorre, K. and B. Thilaganathan, *Maternal cardiac function in preeclampsia.* Curr Opin Obstet Gynecol, 2011. **23**(6): p. 440-7.
18. Ghossein-Doha, C., et al., *Age-related alterations in cardiac geometry in formerly preeclamptic women and healthy parous controls: an explorative study.* Reprod Sci, 2013. **20**(1): p. 39-44.
19. Ghossein-Doha, C., et al., *Hypertension after preeclampsia is preceded by changes in cardiac structure and function.* Hypertension, 2013. **62**(2): p. 382-90.
20. Ghossein-Doha, C., et al., *Maternal cardiac adaptation to subsequent pregnancy in formerly pre-eclamptic women according to recurrence of pre-eclampsia.* Ultrasound Obstet Gynecol, 2016. **47**(1): p. 96-103.
21. Lazdam, M., et al., *Prevention of vascular dysfunction after preeclampsia: a potential long-term outcome measure and an emerging goal for treatment.* J Pregnancy, 2012. **2012**: p. 704146.
22. Lazdam, M., et al., *Elevated blood pressure in offspring born premature to hypertensive pregnancy: is endothelial dysfunction the underlying vascular mechanism?* Hypertension, 2010. **56**(1): p. 159-65.
23. Topouchian, J., et al., *Clinical accuracy of the Omron M3 Comfort((R)) and the Omron Evolv((R)) for self-blood pressure measurements in pregnancy and pre-eclampsia - validation according to the Universal Standard Protocol.* Vasc Health Risk Manag, 2018. **14**: p. 189-197.

For peer review only

24. Rai, T., et al., *Optimizing a digital intervention for managing blood pressure in stroke patients using a diverse sample: Integrating the person-based approach and patient and public involvement.* Health Expect, 2020.
25. Yu, G.Z., et al., *Association of Maternal Antiangiogenic Profile at Birth With Early Postnatal Loss of Microvascular Density in Offspring of Hypertensive Pregnancies.* Hypertension, 2016. **68**(3): p. 749-759.
26. Lewandowski, A.J., et al., *Elevated Blood Pressure in Preterm-Born Offspring Associates With a Distinct Antiangiogenic State and Microvascular Abnormalities in Adult Life.* Hypertension, 2015. **65**(3): p. 607-614.
27. Lewandowski, A.J., et al., *Right ventricular systolic dysfunction in young adults born preterm.* Circulation, 2013. **128**(7): p. 713-720.
28. Lewandowski, A.J., et al., *Preterm heart in adult life: cardiovascular magnetic resonance reveals distinct differences in left ventricular mass, geometry, and function.* Circulation, 2013. **127**(2): p. 197-206.
29. Davis, E.F., et al., *Pre-eclampsia and offspring cardiovascular health: mechanistic insights from experimental studies.* Clinical science, 2012. **123**(2): p. 53-72.
30. Aye, C.Y.L., et al., *Disproportionate cardiac hypertrophy during early postnatal development in infants born preterm.* Pediatr Res, 2017. **82**(1): p. 36-46.
31. Turbeville, H.R. and J.M. Sasser, *Preeclampsia beyond pregnancy: Long-term consequences for mother and child.* American Journal of Physiology-Renal Physiology, 2020. **318**(6): p. F1315-F1326.
32. Agatisa, P.K., et al., *Impairment of endothelial function in women with a history of preeclampsia: an indicator of cardiovascular risk.* American Journal of Physiology-Heart and Circulatory Physiology, 2004. **286**(4): p. H1389-H1393.
33. Kvehaugen, A.S., et al., *Endothelial function and circulating biomarkers are disturbed in women and children after preeclampsia.* Hypertension, 2011. **58**(1): p. 63-69.
34. Sandvik, M.K., et al., *Preeclampsia in healthy women and endothelial dysfunction 10 years later.* American journal of obstetrics and gynecology, 2013. **209**(6): p. 569. e1-569. e10.
35. Munoz-Hernandez, R., et al., *Decreased level of cord blood circulating endothelial colony-forming cells in preeclampsia.* Hypertension, 2014. **64**(1): p. 165-71.
36. Reinisch, A., et al., *Humanized large-scale expanded endothelial colony–forming cells function in vitro and in vivo.* Blood, The Journal of the American Society of Hematology, 2009. **113**(26): p. 6716-6725.
37. Yoder, M.C., *Is endothelium the origin of endothelial progenitor cells?* Arterioscler Thromb Vasc Biol, 2010. **30**(6): p. 1094-103.
38. Gynaecologists, R.C.o.O., *Coronavirus (COVID-19) Infectoin in Pregnancy*, in *RCOG*. 202. p. 67.
39. Sheppard, J.P., et al., *Self-monitoring of Blood Pressure in Patients With Hypertension-Related Multi-morbidity: Systematic Review and Individual Patient Data Meta-analysis.* Am J Hypertens, 2020. **33**(3): p. 243-251.
40. Melchiorre, K., et al., *Preeclampsia is associated with persistent postpartum cardiovascular impairment.* Hypertension, 2011. **58**(4): p. 709-15.
41. Huckstep, O.J., et al., *Physiological Stress Elicits Impaired Left Ventricular Function in Preterm-Born Adults.* J Am Coll Cardiol, 2018. **71**(12): p. 1347-1356.
42. McManus, R.J., et al., *Efficacy of self-monitored blood pressure, with or without telemonitoring, for titration of antihypertensive medication (TASMINH4): an unmasked randomised controlled trial.* Lancet, 2018. **391**(10124): p. 949-959.
43. Grant, S., et al., *Using mHealth for the management of hypertension in UK primary care: an embedded qualitative study of the TASMINH4 randomised controlled trial.* Br J Gen Pract, 2019. **69**(686): p. e612-e620.
44. Strobl, I., et al., *Left ventricular function many years after recovery from pre-eclampsia.* BJOG, 2011. **118**(1): p. 76-83.

For peer review only

45. Boardman, H., et al., *Variations in Cardiovascular Structure, Function, and Geometry in Midlife Associated With a History of Hypertensive Pregnancy.* Hypertension, 2020. **75**(6): p. 1542-1550.
46. Boardman, H., et al., *Aortic stiffness and blood pressure variability in young people: a multimodality investigation of central and peripheral vasculature.* J Hypertens, 2017. **35**(3): p. 513-522.
47. Kalafat, E. and B. Thilaganathan, *Cardiovascular origins of preeclampsia.* Curr Opin Obstet Gynecol, 2017. **29**(6): p. 383-389.
48. Thilaganathan, B. and E. Kalafat, *Cardiovascular System in Preeclampsia and Beyond.* Hypertension, 2019. **73**(3): p. 522-531.

For peer review only

FIGURE LEGENDS

Figure 1: Illustration of self-management of blood pressure for women randomised to the intervention

Figure 2: Flow Chart of Proposed Study visits

TABLE LEGENDS

TABLE 1: List of study aims and objectives with respective timepoint (s) for the measurement of each objective

TABLE 2: Summary of the power calculations used to determine the trial sample size for the primary, and key secondary outcomes

TABLE 3: Adverse Event Definitions

For peer review only

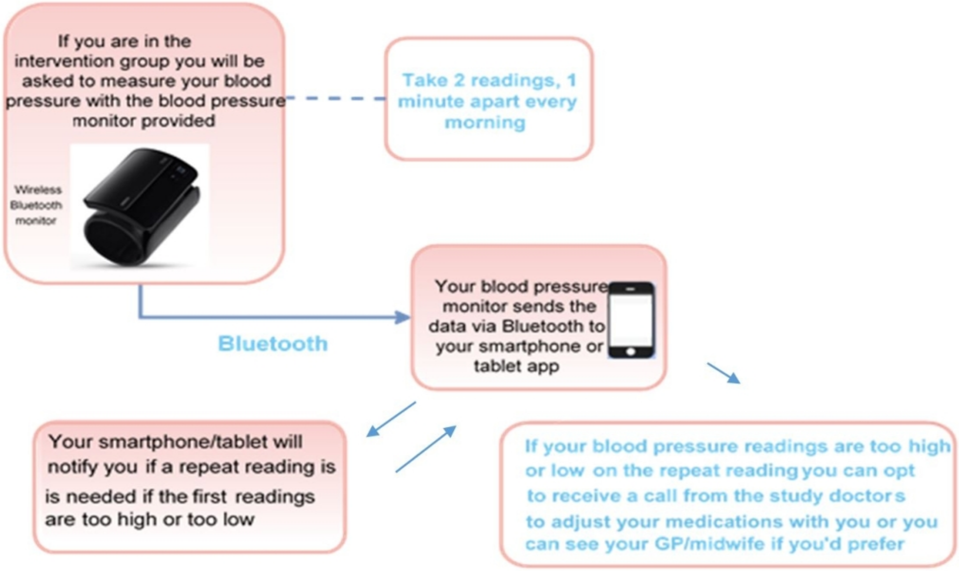

Figure 1: Illustration of self-management of blood pressure for women randomised to the intervention

152x90mm (240 x 240 DPI)

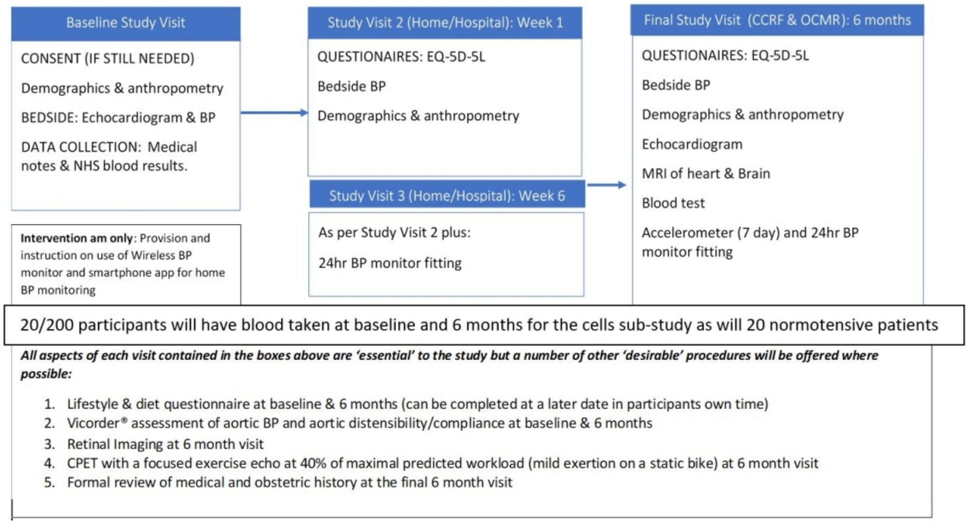

Figure 2: Flow Chart of Proposed Study visits

155x89mm (240 x 240 DPI)

APPENDIX A: TRIAL FLOW CHART

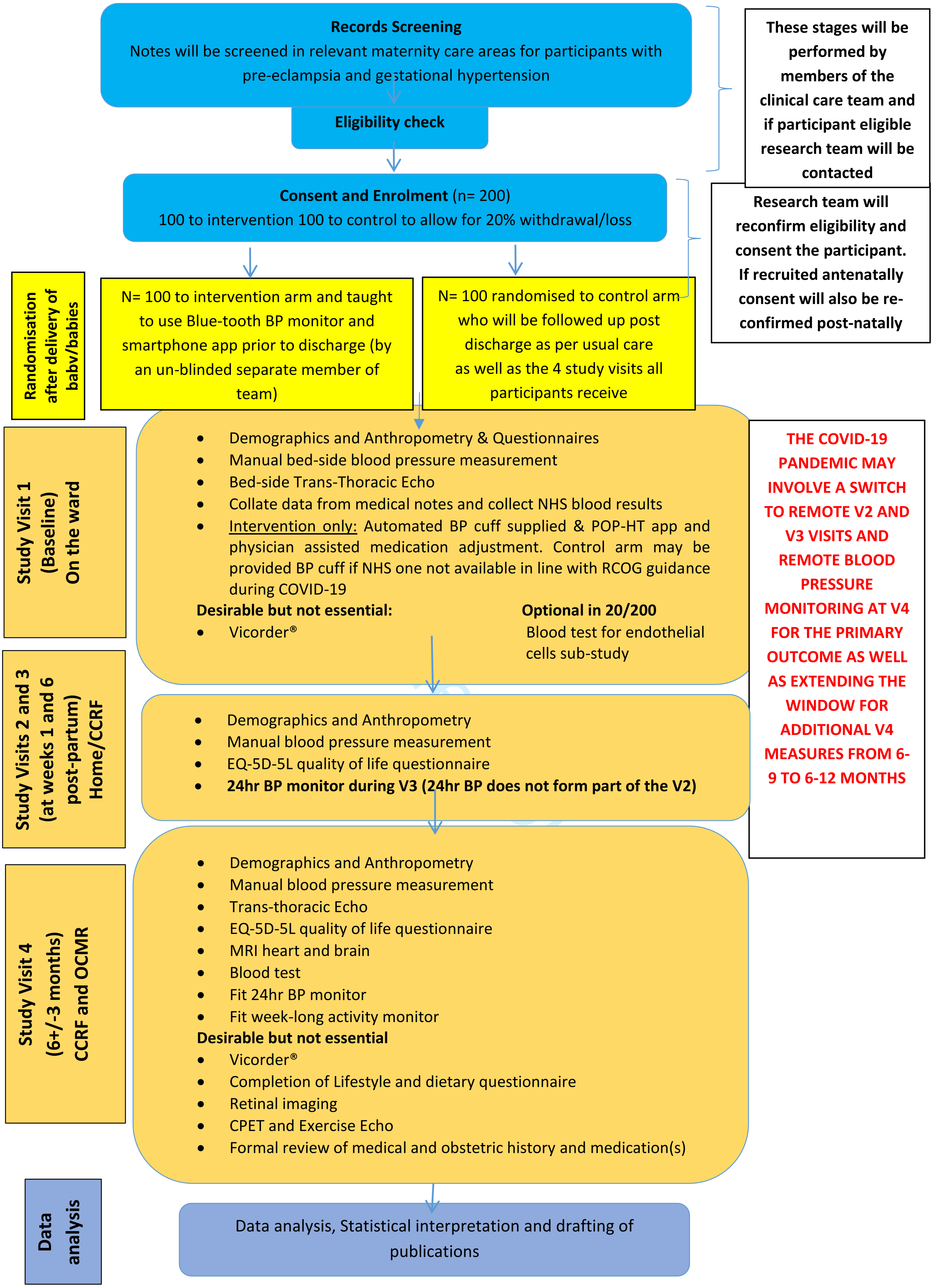

APPENDIX B: SCHEDULE OF PROCEDURES (MAIN STUDY)

**Procedures**	**Visit 0: Consent**		**Visit 1: Baseline**		**Visit 2**		**Visit 3**		**Visit 4**	
	Intervention arm	Control arm	Intervention arm	Control arm	Intervention arm	Control arm	Intervention arm	Control arm	Intervention arm	Control arm
Eligibility assessment	X	X								
Informed consent	X	X								
Clinic/Bed-side BP measurement			X	X	X	X	X	X	X	X
Demographics & anthropometry			X	X	X	X	X	X	X	X
Echocardiogram			X	X					X	X
Data collection: medical notes and NHS blood results			X	X						
Lifestyle & Diet questionnaire			X	X					X	X
Vicorder ®(vascular assessment)			X	X					X	X

For peer review only

<u>Intervention:</u> Automated BP cuff provision and Smartphone app installation			X	During pandemic cuff may be provided						
Home BP self-monitoring and physician assisted medication adjustment post hospital discharge					X		X		X	
EQ-5D-5L questionnaire					X	X	X	X	X	X
**Fitting and performing 24hr ABPM (V3 & V4 only)**							X	X	X	X
MRI of heart and brain									X	X
Blood test			X (if in cells sub-study)	X (if in cells sub-study)					X	X
Fit accelerometer									X	X
Retinal imaging									X	X

For peer review only

Review of medical and obstetric history									X	X
CPET with exercise echo at 40% workload									X	X

For peer review only

APPENDIX C: PROCEDURES FOR ENDOTHELIAL CELLS SUB-STUDY

Procedures	Visit 1: Baseline		Visit 2	
Eligibility assessment	X	X		
Informed consent	X	X		
BP measurement	X	X	X	X
Demographics & anthropometry	X	X	X	X
Blood test	X	X	X	X

For peer review only

APPENDIX D: POP-HT Tele-monitoring system software and network architecture

The software and network architecture of the POP-HT INTERVENTION can be summarised as follows (from left to right in Figure D.1 below): the participants first take BP readings using a Bluetooth-low-energy (BLE) enabled Blood Pressure device (OMRON Evolv®). The data are transmitted to their POP-HT App, available on both Android and iOS mobile devices, via BLE. The App communicates with the POP-HT web-application, available from a public domain on the internet, over encrypted HTTPS, using Representational state transfer (REST) Application Programming Interfaces (APIs), secured by the JSON Web Token protocol. The web-application runs on a web-server hosted in an authorised and secured virtual machine from Oxford University Hospitals (OUH). It includes rule-based algorithms (based on the on/off treatment BP tables below) that process the participants' BP readings and output: (i) the BP level, (ii) the participants' next action, and (iii) the suggested frequency of BP readings. The web-application uses an App notification service (Firebase, Google, USA, https://firebase.google.com) to send messages to the participants' Apps, such as missing data warnings and medication changes. An SMS gateway service (Esendex, UK, https://www.esendex.co.uk) is also used, as a backup, to send messages in case the notification service becomes temporarily unavailable. The SMS system is also responsible for sending and recovering login credentials. E-mails are sent twice daily to clinical researchers, using NHS SMPT servers, summarising the BP and medication status, including triggers to down-titrate, of all study participants in the POP-HT system. The web-application allows participants to review all of their data while e.g., only the last 2 weeks or messages and BP are displayed on the App. Finally, the web-application also allows clinical researchers to register new participants, create and manage their treatment plan, review the participants dashboard, resolve abnormal BP and missing data flags, and export pseudo-anonymised data (identified only by the study IDs) to carry out statistical analysis.

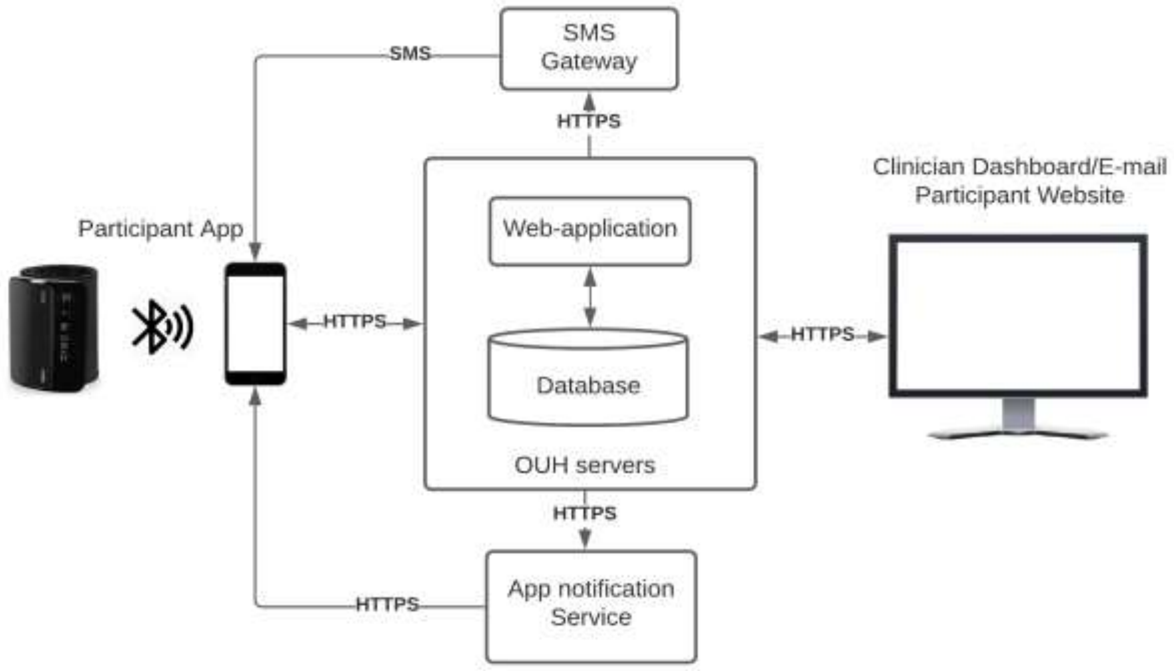

Figure D.1: Illustration of the POP-HT tele-monitoring system software and network architecture

Process of Self-Management

The decisions outlined below in Figure D.2 are based on the participant taking two consecutive BP readings (2 minutes apart), and the automated algorithm decision being made on the second reading. On this note, if more than 2 readings are taken by the participant, all readings are synchronised from the monitor and submitted to the OUH-NHS server, but the decision is made only on the last reading.

Figure D.2 Traffic light table of BP ranges, BP classification and pre-programmed actions whilst ON treatment

Colour	Level	BP	Action
Red	Very high	Sys 160 or more OR Dia 110 or more OR Symptoms	Repeat BP in 5 minutes. If this is a repeat reading: contact local maternity unit immediately for urgent assessment today.
Orange	High	Sys 150-159 OR Dia 100-109	Repeat BP in 5 minutes. If this is a repeat reading: Call from study physician 9-5pm AND to see own GP/midwife for an URGENT (same-day) appointment.* Switch to twice daily readings until back in yellow/green
Yellow	Raised	Sys 140-149 OR Dia 90-99	No action.
Green	High normal	Sys 130-139 OR Dia 80-89	No action
Blue	Low normal	Sys 100-129 AND Dia < 80	Switch to twice daily readings and if in this zone for 2 consecutive days, medication titration will be signed off by study doctor and instructions sent via app to participant
Purple	Low	Sys < 100 AND Dia < 80	Repeat BP in 5 minutes. If this is a repeat reading: option of opting for a call from study physician 9-5pm vs. opting to see own GP/midwife for an URGENT appointment*. Switch to twice daily readings until back in yellow/green

*During COVID-19, NHS clinicians may not see the patient in person the same day but a tele-medicine review at the minimum will be advised.

If the participant has no 3G/4G signal or Wifi when they synchronise the OMRON readings to the phone, and/or there is an error in the transmission of the readings to the OUH-NHS server, the readings are deemed as 'valid' by the App for 4 hours. After 4 hours, if the participant enters the 'Enter my readings' section and tries to re-sync with the server, they will be asked to take new readings with the OMRON. Once the synchronisation with the server is re-established, all readings, regardless of their timestamp will be sent to the server. The SMS system is also a backup to allow the participants to notify the study doctors of their readings. The messages used are essentially the same, small changes only being required to map the same App functionality to the more "limited" SMS system.

The frequency of readings requested of the participant via the app will be adjusted according to whether the readings are high, low normal or low. If they are in the orange, blue or purple

zones above (high, low normal or low), they will be asked by the app to submit readings twice daily.

If the reading is abnormal, as outlined in the table they will be asked to 'Repeat the BP in 5 minutes'.

If the repeat reading submitted is in the red zone i.e.>160/110mmHg they will be sent a notification asking them to contact their local maternity unit immediately for urgent assessment (see 'Safety Netting' section below for more detail).

If the repeat reading is in the orange or purple BP ranges above, participants will be notified via an app notification and will have the option of:

- Selecting via the app to be called by a 'physician' (who will be a clinician within the research team) to discuss and make medication adjustment over the telephone,
- And seeing their GP/Midwife/maternity unit on an urgent basis to have their medications adjusted, which they will then update on the app.

Medication titration will be done in line with the updated NICE guidance NG133 in the intervention arm. During COVID-19 given the difficulties in seeing their own GP/mid-wives, it is expected more women will want remote physician support. The software will send a notification to the participant using more 'participant friendly' wording as agreed in several rounds of PPI and as tested on real-life volunteers as part of the PPI process: For example, if the blood pressure reading submitted is 152/100mmHg it will trigger this notification:

'*Your blood pressure reading is a little higher than we would ideally like it and the repeat reading was also a little high. Select* ***Yes*** *to be called by a specialist doctor to have your medication adjusted or* ***No*** *to see your own GP/midwife/maternity unit urgently?*

They then have the option of selecting **'YES'** or **'No'** on the app which triggers the following actions to be taken

- **Yes**: The study team will call them the same working day (if out of hours, at the beginning of the next working day) to review their symptoms and to adjust their medications. If the doctor calling has any concern or if the participant feels unwell prior to the call then they will be aware of the need to see a health professional from the information sheet, the app and/or the call, as appropriate. During the call, they will be advised of the need to have blood tests checked if this reading is in the 1st 2 weeks' post-partum in the orange zone. This should be done over the next 24 hours by the GP/local maternity unit. They will also be asked to monitor twice daily until 2 sets of readings are back in the normal (yellow/green zones). The medication schedule will be updated on the web-platform after the call and synchronise to the app so it will be written down for the participant too. The next day the participant will be asked to confirm their new medication schedule on the app with a YES/NO as a safety check. If it is NO they will be asked to enter, what they are taking and any discrepancy will be clarified with the research team over the phone.
- **No**: The app will notify them that urgent midwife/GP assessment is advised and the team will call the same day to check this is in progress and enquire about any medication adjustment. They will also be asked to monitor twice daily until 2 sets of readings are back in the normal (yellow/green zones).

'Safety netting' built into the system

The app has been tested for in detailed simulation scenarios e.g. high BP, low BP, fluctuant BP, non-compliers, anxious and for all eventualities based on our experience from SNAP-HT [7] and it has proved robust in these 'test scenarios'. Automatic e-mail alerts will be triggered

For peer review only

to the clinical members of the study team and flags will generate on the website next to that participant's study ID, for those BP readings 'out of target range', and/or if readings are consistently not being recorded and/or uploaded via the app by a particular participant. These secure e-mails will be sent (as explained further in appendix E) so that appropriate action can be taken in a timely fashion to adjust medications for those readings out of range as explained above. For those readings in the red zone, a phone call will also be made that same working day (9-5pm) to ensure arrangements have been made for assessment by an NHS provider, and in the event they cannot be contacted, their GP/midwife will be notified.

Once participants have switched to once weekly readings i.e. they have been off-treatment for 5 days with readings in the normal range, and have been notified by the app to make this switch, motivational reminders will be sent on a weekly basis. If the readings go up or down outside of the normal range whilst doing once weekly readings i.e. if they are not in the green or blue zones, they will be asked to repeat the reading and if the repeat reading is also outside of these zones, the relevant actions will be triggered as outlined below in figure D.3

Figure D.3 Traffic light table of BP ranges classification and pre-programmed actions whilst OFF treatment

Colour	Level	BP	Action
Red	Very high	Sys 160 or more<br>OR<br>Dia 110 or more	Repeat BP in 5 minutes.<br>If this is a repeat reading*: contact your local maternity unit immediately for urgent assessment today. Switch to once daily readings
Orange	High	Sys 140-159<br>OR<br>Dia 90-109	Repeat BP in 5 minutes.<br>If this is a repeat reading and the value is in this range for 2 or more days in a row option of opting for a call from study doctors between 9-5pm vs. opting to see own GP/midwife in next 48hrs. Switch to once daily readings until further notification
Green	Normal	Sys < 140<br>OR<br>Dia < 90	No action. Continue weekly readings
Blue	Low normal	Sys 100-129<br>AND<br>Dia < 80	No action unless symptoms (report via the app/website) e.g. light-headed/dizzy/faint in which case please notify study team who will call you within 48 hours
Purple	Low	Sys < 100<br>AND<br>Dia < 80	Repeat BP in 5 minutes.<br>If this is a repeat reading and the value is in this range for 2 or more days in a row option of opting for a call from study doctors between 9-5pm vs. opting to see own GP/midwife in next 48hrs. Switch to once daily readings until further notification

*If the repeat reading is 'Red' a flag/notification will also be sent to the study team to call the participant to check they have followed the action just as for the on-treatment algorithm. For orange and purple zones, the research team will also be notified and the participants will be asked to switch back to daily readings. If the readings remain outside of the green/blue zones for >2 days the app will offer the option of being contacted shortly by the study team or opting

to see their own GP/midwife, as per the pathways for the on-treatment algorithm. It is unlikely that women will need to-restart treatment based on the pilot data from SNAP-HT [4], where only 1/91 women needed to re-start medication after stopping.

In the case of failure to submit readings, automatic 'motivating' notifications will be sent at 24hours to the participants, and notifications are also sent to the study team after 36 hours without a reading during the first 2 weeks following discharge. This is to prompt a call to the participant to discuss any problems. If the participant repeatedly fails to submit a reading for >36 hours during the first 2 weeks, then they may be withdrawn from the study at the discretion of the PI/CI. Their GP will also be called and mailed; and the participant will be called, messaged and e-mailed advising urgent medical review.
Similar 'safety alerts' will be sent out if a participant records any side-effects/SAEs (which can be accessed and reviewed by the study team via a secure log-on portal). If there is anything deemed to require further action the PI/CI, GP and participant will be notified urgently.

APPENDIX E: MODIFICATIONS TO STUDY DESIGN TO MITIGATE THE IMPACT OF COVID-19

On March 17th 2020, the COVID-19 pandemic meant that all research across Oxford University Hospitals (OUH) NHS Foundation Trust had to be halted. A minor amendment to perform remote V2 and V3 visits allowed us to continue the follow up of 18/200 women already recruited as of 06/05/2020. A further two amendments allowed the POP-HT RCT to restart safely during the COVID-19 pandemic as the study received a Stage 3 exception from OUH NHS Foundation Trust, due to its contribution to the clinical care of these women. Recruitment to the trial recommenced in early June 2020.
The following changes are explained in more detail below to explain how the study will run safely and in line with local COVID-19 guidance:
Remote study visits were established early to allow entirely remote follow up visits for week 1 and week 6, equivalent to visits 2 and 3 (non-substantial amendment 2.0 25/03/20201). The 1st 18 participants' recruited pre-COVID had been followed up remotely successfully, demonstrating the technique to be both effective and feasible for the remaining 182/200. None of the 1st 18 participants required face-face contact for review of their medications and obstetric history, or for use the POP-HT app and the solving of any technical issues for those in the intervention arm. Remote blood pressure measurement (both clinic and ambulatory 24hr blood pressure monitoring) was achieved successfully for all of the 1st 18 participants recruited pre-COVID 19 using ZOOM® or MS TEAMS®. During this period of follow up, no new baseline visits were performed during the 1st wave of the COVID-19 pandemic.
When the study restarted recruitment in June 2020 the following amendments were made to the baseline visit with re-design of the recruitment, consent and enrolment process to minimise direct patient contact and risk of virus transmission:

a. Provision of documents: PIS, flyer and additional information sheet provided by the clinical team to the participant on a Tablet/Ipad® (sterilised with CLINELL® wipes). The participants can then review them in this format (and a copy e-mailed to them for their records once consent has been obtained and they are enrolled).
b. Consent: Consent forms will be placed in wipe down wallets, which will be handed to the participant for signing wearing sterile gloves. Once signed the form will be photocopied whilst wearing gloves. The copy will then be placed

For peer review only - http://bmjopen.bmj.com/site/about/guidelines.xhtml

back into a sterile wallet for the participant and the original will be placed in a second wallet in their notes. Both will be wiped down with CLINELL® wipes and the research teams' copy will be kept securely in a wipe-down file/ring-binder in 'quarantine' before moving them to CCRF after 48hours.

c. The Vicorder® was made an optional measurement to reduce the amount of time in direct patient contact

d. The Echo and Vicorder® will be performed by a single investigator at the bedside. PPE will be worn (the level of which will be in line with hospital policy). Adequate training in donning and doffing of PPE has been undertaken by the study team via OXSTAR®. This will not affect OUH trust protocols for female chaperones, which can still be provided if needed by existing clinical staff on the ward.

e. In line with RCOG guidance, produced during COVID-19 in June 2021, the control arm will also provided with a home monitor on discharge for the first 'few weeks'

f. The V2 and V3, at weeks 1 and 6 respectively, will be continued remotely for participant 19 onwards once recruitment restarted.

g. V4 can also be done remotely for the primary outcome measures if a national lockdowns necessitate such a step to be taken.

APPENDIX F: AMENDMENT HISTORY

All amendments have been submitted to; and approved by SPONSOR, the REC and HRA, the local hospital (OUH) trial management authority; and other relevant parties are notified where needed.

Amendment No.	Protocol Version No.	Date issued	Author(s) of changes	Details of Changes made
01 (minor)	N/A	21/01/2020	J Kitt	Correction to wording of consent form clauses<br><br>Correction of version listed in flyer footer<br><br>IRAS form updated to list PI for OUH site
02 (minor)	V2.0	19/03/2020	P Kemp	Add option for Visit 2 (Weeks 1) and Visit 3 (Week 6) to be conducted remotely by video call.<br><br>Add the option of sending a sterile OMRON EVOLV BP monitor to participants prior to Visit 3 (Week 6).<br><br>Make the "fitting of a home blood pressure monitor" procedure optional for Visit 3 and add the option

				of conducting this at a later time point.
03 (minor)	V3.0	22/04/2020	J Kitt and P Kemp	Changes to visits during the COVID-19 pandemic:<br>Add provision of BP monitor to control arm;<br><br>Consent process to be modified to reduce risk of transmissions of COVID-19;<br><br>Extend the time point for Visit 4 to 6-12 months from 6-9;<br><br>Change the randomisation and blinding process so that only one member of staff (wearing PPE where necessary) is present at Visit 1;<br><br>Minimise participant contact during the baseline visit via use of tablets/iPads for reviewing the PIS/flyer;<br><br>Vicorder is now an optional measurement during the baseline visit.
04 (minor)	V4.0	06/10/2020	A Frost and P Kemp	Add blood test to baseline visit in both control and intervention hypertensive groups.<br><br>Include a sub population of 20 normotensive postnatal women for a new blood validation sub study.
05 (minor)	V5.0	05/01/2021	J Kitt and P Kemp	Add option for Visit 4/Final visit to be performed remotely by video call for the primary study outcome and BP based secondary outcome measures
06 (major)	V6.0	08/03/2021	J Kitt and P Kemp	Addition of an extra sequence during the MRI scan at 6-12months to allow the kidneys to be evaluated at the same time as the heart and brain sequences are being performed. Scan duration will still remain under 60 minutes

For peer review only

APPENDIX G: COPIES OF PARTICIPANT INFORMATION SHEET (PIS) AND INFORMED CONSENT FORMS (ICF)

Oxford University Hospitals NHS Foundation Trust

UNIVERSITY OF OXFORD

CCRF Cardiovascular Clinical Research Facility

POP-HT

Division of Cardiovascular Medicine
Radcliffe Department of Medicine &
Nuffield Department of Primary Care Health Sciences,
Oxford Heart Centre, John Radcliffe Hospital,
Oxford, OX3 9DU Tel: 01865 572833
Email: jamie.kitt@cardiov.ox.ac.uk

Physician Optimised Post-partum Hypertensive Treatment (POP-HT) Study

Participant Information Sheet (PIS): Information about a study you are invited to join

We would like to invite you to take part in a research study. Joining is entirely up to you. This information sheet explains why the research is being conducted and what it would involve if you did decide to take part. If you have any questions, please do not hesitate to ask. Please feel free to talk to others about the study.

Summary of the study

- **High blood pressure occurs in ~1 in 10 women during pregnancy** and **remains elevated in 50% even after the baby is born requiring medication at home after discharge.**
- This **study** is **investigating** whether, **'self- management' of blood pressure (BP) at home**, **after discharge**, can improve your blood pressure control and reduce the longer-term impact on your heart and brain.
- **50%** of participants (**the self-management group**) will be asked to **measure their blood pressure at home** using a **Wireless monitor** and **upload the readings via** a **smart-phone/tablet app.** If readings are abnormal then a specialist doctor can guide your medication adjustment accordingly (you will still have option of seeing your own GP/midwife if you prefer).
- **50%** (**the control group**) **will be looked after by their GP/ midwife/other NHS services as normal**. The group you are in will be decided by random. During the COVID-19 pandemic a BP monitor will also be provided to the control arm to allow home readings to be measured as per updated national guidelines. See page 11 for other amendments during the COVID-19 pandemic.
- The **study starts after giving birth** and **runs up-to 12 months** after discharge from hospital and **both the intervention and control group will have 4 study visits** as part of the trial.
- The team are aware that you will have a new born baby/babies so **study visits will be very flexible** and can stop-start around feeding, nappy changes and other important baby needs.

N.B. All women will continue to receive routine NHS care throughout.

Chief Investigator:	Prof Paul Leeson	Version/date:	6.0 23.03.2021
Study Short Title:	POP-HT STUDY	Ethics Ref:	19/LO/1901
		IRAS ID:	273353
Document:	Participant Information Sheet		

Why we are doing this study?

High blood pressure disorders in pregnancy are associated with an increased risk of high blood pressure, heart attack and stroke in later life. The risks associated with high blood pressure in pregnancy can be mitigated by early recognition and treatment of raised blood pressure (and other traditional risk factors e.g. lack of regular exercise, an unhealthy diet. This trial is looking at the impact that blood pressure control has on these long-term risks. We plan to assess whether blood pressure self-management can improve blood pressure control and whether this reduction in blood pressure in the months after birth can reduce the long-term effects these conditions have on your heart, brain and blood vessels, in turn reducing the risk of the events listed below, which include:

1 in 5 women having another hypertensive pregnancy

1 in 7 women having another pre-eclamptic pregnancy

A 4x increase in risk of having long term high blood pressure

A 2x increase in risk of experiencing both cardiovascular death or a heart attack

A 1.5x increase in risk of having a stroke

(Figure adapted from NICE NG 133: Hypertension in pregnancy July 2019)

Why have I been invited?

If you have pre-eclampsia or gestational hypertension and still require medication after delivering your baby/babies at the time of discharge.

Do I have to take part?

No, this is a voluntary study, and it is your decision to participate. You are free to withdraw from the study at any time without giving a reason. This would not affect the standard of care you receive. If you decide that you no longer wish to continue with the study, we would still retain any data already obtained from you unless you request otherwise.

What will happen if you take part in the POP-HT study:

Both groups receive the same number of study visits i.e. 4 visits for all participants.

Visit 1 (90 minutes) will take place in the first days after giving birth whilst you remain on the post-natal ward in the Women's centre. We will **measure your blood pressure**, **scan your heart (using an ultra-sound scan like you had of the baby), take a blood test and review your medical notes and blood tests** and e-mail a **questionnaire about your lifestyle and diet,** which can be completed later to

Chief Investigator:	Prof Paul Leeson	Version/date:	6.0 23.03.2021
Study Short Title:	POP-HT STUDY	Ethics Ref:	19/LO/1901
		IRAS ID:	273353
Document:	Participant Information Sheet		

For peer review only

shorten the visit**. At the end of visit 1** you will either be **allocated at random** to the **intervention group** or **the control group.** If allocated to the **intervention** group a separate member of the team will come and provide you with the blood pressure monitor and install the app on your smartphone/tablet and teach you how to use it. You will have plenty of time to practice whilst still in hospital!

Visits 2 and 3 (30 minutes) take place at **weeks 1 and 6 weeks after discharge respectively** and are to measure your blood pressure, take some simple measurements e.g. waist, left arm and hip circumference, and to complete a brief questionnaire. These can be done as a **home visit**, or as a visit to us in the Cardiovascular Clinical Research Facility (CCRF) at the **John Radcliffe Hospital**. At the end of **Visit 3** (week 6) there will be a 24 hour blood pressure monitor fitted, programmed to be silent to minimise disruption to you and your baby, and we will provide you with a stamped, addressed envelope to post it back to us at CCRF.

At 6-12 months there will be a **slightly longer visit to our research facility** (CCRF) in the **John Radcliffe Hospital. Visit 4** (up to 4 hours) will involve measuring your blood pressure again, doing another scan of your heart by ultra-sound, doing an MRI (magnet scan) of your heart and brain, and taking a blood test. There will also be a brief review of your medical and obstetric history and medications, and a few other tests (not absolutely mandatory), which include: taking photos of the blood vessels in the back of your eyes, and doing some gentle exercise on a bike (akin to walking up a hill at a fast pace) during which we measure your heart rate, blood pressure and, scan your heart briefly with the ultra-sound machine. The study finishes with another period of 24hr blood pressure monitoring and a wrist-watch (accelerometer) you wear for 1 week. ***The additional PIS you have also been given provides more detail specifically about this 4th visit. We will run through this again at the end of your 3rd visit at 6 weeks so you can have a chance to discuss any questions and we can help plan child care for the 4th and final longer visit at the John Radcliffe Hospital.***

We would like to follow you up for up to 10 years. For this longer term follow up, the research team in Oxford will ask for information about your health from NHS Digital (we will send your name, date of birth, NHS number and postcode to NHS Digital and ONS (or other central NHS bodies) who can link this information to your centrally held records to allow your blood pressure records to be reviewed if needed. We will access these records so that we can assess long term health outcomes and in particular monitor your long term blood pressure control in line with one of the study objectives.

We may also want to measure your blood pressure again in the future (up to 10 years from enrolment) as a home visit/visit to the hospital. This will be an extension to this study and we would like to contact you again to ask you to consider further participation.

Further details of the individual study procedures over the 6-month period are as follows:

For peer review only - http://bmjopen.bmj.com/site/about/guidelines.xhtml

1. Bed-side blood pressure measurement (10 minutes): Three blood pressure readings will be taken at intervals of 1 minute from your left arm (unless there is a medical reason not to use the left) using an automated blood pressure monitor. This will require you sitting at rest for 5 minutes prior to doing any measurements.
2. Echocardiogram scan (15 minutes): We will perform an ultrasound (echocardiogram/echo) of your heart. This is a safe and painless procedure and you will be asked to lie on a couch on your left side. A probe is placed on your chest and lubricating jelly is used so the probe makes good contact with the skin. Ultrasound waves then create images of your heart on the scanner monitor. It normally takes 15 minutes to acquire these images. A female sonographer/scanner will be provided wherever possible and if not available, a female chaperone will be available.
3. Vicorder® (Vascular Measures and Central Blood Pressures, 10 minutes): This involves lying flat on a couch and having two blood pressure cuffs fitted, one to the right arm and one to the right leg. These are inflated and deflated three times at 1-2 minute intervals. This is now an optional measure during the COVID-19 pandemic.
4. Lifestyle and diet questionnaire (25 minutes): The questionnaire combines validated questions used in previous studies. Information will be collected on factors that affect blood pressure including: smoking frequency, alcohol and salt intake, exercise and family history. Questionnaires can be completed either during a study visit or at a later date and posted back to the study team (pre-paid envelopes will be provided). Some of these questions may not seem relevant as they are taken from validated questionnaires used across a range of ages and in both males and females. The team will explain any such questions.
5. EQ-5D-5L Quality of Life questionnaire (5 minutes): You will be provided with an EQ-5D-5L questionnaire, a widely used and validated way of assessing quality of life. A trained study investigator will run through the structured questionnaire during the visit with you.
6. MRI of the Heart, Brain, aorta and kidneys (1 hour including break): As part of this study you will have an MRI scan of your heart and brain. The MRI scanner is shaped like a polo mint, the hole inside measuring about 60 centimetres wide. MRI is safe and non-invasive and does not involve any ionising radiation (x-rays). However, because they use a large magnet to work, MRI scans are not suitable for everybody. Because of this, you will be asked pre-screening safety questions to help determine if you are able to take part. More detail about the MRI scan is provided on the *Supplementary Information Sheet* given to you before the 4th study visit. This additional information sheet also provides more detail about the optional sub-study of having gadolinium given (a commonly used contrast drug for MRI) to acquire an extra few images if you are no longer breast-feeding at that time-point. Additional consent will be sought and obtained for this sub-study prior to the scan being performed.
7. Blood test (10 minutes): The equivalent of 5 teaspoons of blood will be collected by a trained member of staff. We will try our best to time it with blood tests requested by your clinical care team whilst you are an inpatient. You do not need to be fasted for this test. We use very small

Chief Investigator:	Prof Paul Leeson	Version/date:	6.0 23.03.2021
Study Short Title:	POP-HT STUDY	Ethics Ref:	19/LO/1901
		IRAS ID:	273353
Document:	Participant Information Sheet		

'butterfly' needles to minimise any discomfort. You may experience some bruising and discomfort at the site where you have your blood taken. Our staff are highly trained in blood taking and we will make sure you are as comfortable as possible.

8. **Fitting of a home 24 hour blood pressure monitor (5 minutes, to be worn for 24 hours) will be done only during V3 at week 6 and during the final visit (V4). The activity monitor (to be worn for 7 days and nights) is only fitted during V4**:
   The 24hr monitor consists of a blood pressure cuff, which will be fitted on the left arm (right if a specific medical reason precludes use of the left) and a BP monitor. A small bag will also be provided that is worn around the waist or shoulders, in which the monitor is placed. You will be shown how to re-attach the cuff to the monitor e.g. after a bath/shower. The BP monitor will be silent and automatically inflate hourly during the day and every other hour at night to minimise inconvenience at this busy time. Participants will be asked to wear the monitor for 24 hours. We provide you with an information sheet about how to use the monitor and deal with frequent problems and this also has a brief blood pressure monitoring diary on the back for you to detail the time you went to sleep/woke up and any periods of activity that may have put your blood pressure up e.g. running for the bus/cycling to work. The activity monitor (wrist-watch) is waterproof and shock-proof and will be worn continually on the wrist for one week. Stamped, addressed envelopes will be provided to return both devices after use.
9. Retinal imaging (10 minutes): Photos will be taken of the back of your eye just like at an optician. More information about this is provided in the *Supplementary Information Sheet*. To help keep your information confidential, your images will be 'de-identified' and assigned a study code. However, your retinal images are unique to you so they can never be completely anonymous.
10. Cardiopulmonary Exercise Testing (CPET) with exercise echo (30 minutes): This involves gentle cycling on a stationary bicycle and doing a short ultrasound (echocardiogram) of your heart whilst exercising. More information about this is provided in the *Supplementary Information Sheet*.

The Self-management (intervention) group: Taking your blood pressure at home

This section only applies to participants who are allocated to the intervention group and an additional intervention group information sheet will be provided for you as a paper copy, on the app and on the website.

You will be asked to **start measuring your blood pressure** on the **day of discharge** from hospital using the OMRON Evolv monitor provided. This will mean taking **2 readings, 1 minute apart** every morning after discharge. **We will ask you to do this every morning, until you have had 5 consecutive days**

For peer review only - http://bmjopen.bmj.com/site/about/guidelines.xhtml

with blood pressure readings in the normal range (off medication). We anticipate this to take approximately **2-3 weeks following discharge** based on our experience in our pilot study.

After you measure your blood pressure, you will **open up the POP-HT app** on your smartphone/tablet, confirm it was you that took the blood pressure readings and **click 'SYNC'.** The app will then 'synchronise' with the OMRON EVOLV® monitor to **upload the reading to the app and the secure study website** hosted on the hospital's secure intranet (*not the internet*). **See figure one below for an illustration of how this is done**

Your smartphone/tablet will notify you if your readings are too high, too low or in the normal range. If they are high or low the app will ask you to repeat one more reading. If the reading is still high/low the app will notify you and the specialist study doctors. **You can then opt to receive a call from them to help adjust your medication and will be advised to make an urgent/same-day appointment with your own GP/midwife if necessary** (further detailed information about this is available in the self-management information sheet). Medication will be gradually titrated down until you are off all medication.

Once you have 5 days in a row with readings in the normal range (off medication) you will be sent a notification/message reading "Thank you. Your blood pressure readings have all been normal since stopping treatment. Please **change to once weekly readings** for the remainder of the study." This will be **until 6 months** after the delivery of your baby. The **reason** for the **longer period of weekly monitoring** is to **ensure you do not have a late rise in your blood-pressure readings** that requires further treatment.

Figure 1: Illustration of how self-monitoring will be performed in the intervention group

For peer review only

Chief Investigator:	Prof Paul Leeson	Version/date:	6.0 23.03.2021
		Ethics Ref:	19/LO/1901
Study Short Title:	POP-HT STUDY	IRAS ID:	273353
Document:	Participant Information Sheet		

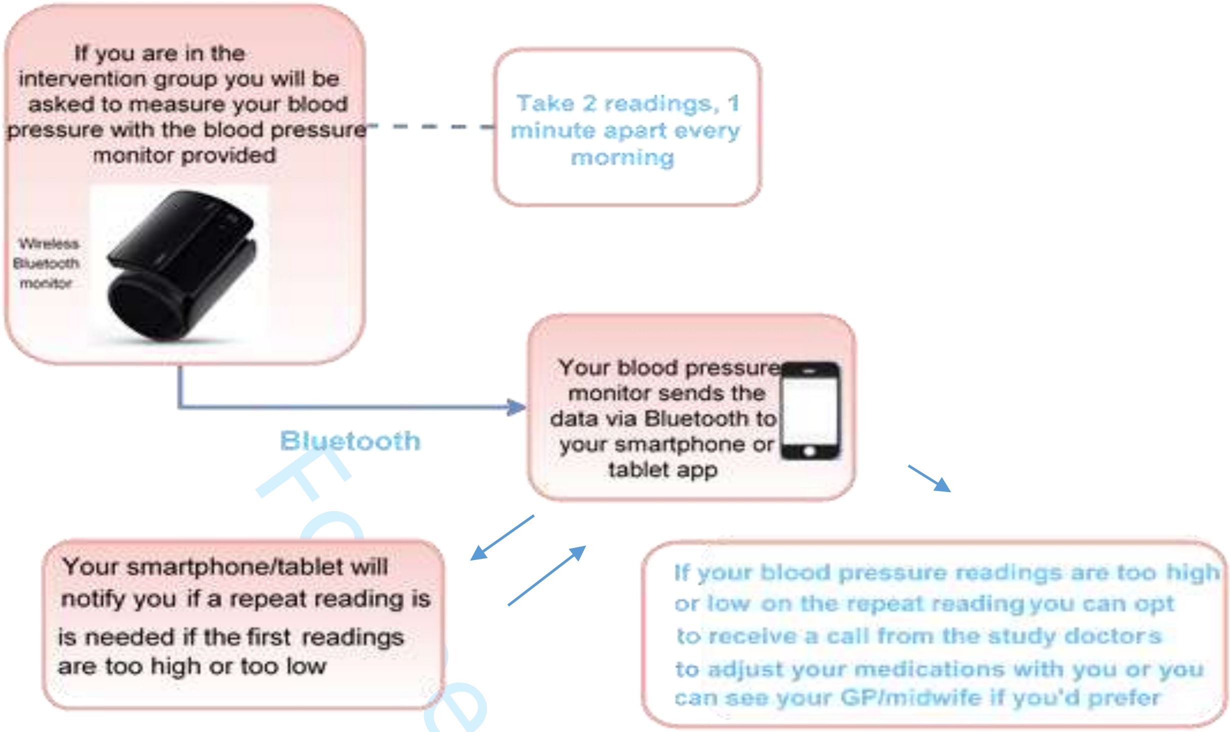

The control group

If you are allocated to the control group, you will be looked after by your GP/midwife/Health visitor as per usual NHS care in addition to the 4 study visits described above. **You will also be given a blood pressure monitor for the first few weeks, during the COVID-19 pandemic, as per updated national guidance**. This is to facilitate care from your GP and mid-wife who may not be able to do home visits as they normally would during the pandemic. Instead, your self-monitored readings can be reviewed by a tele-medicine appointment (when a face-face review is not possible due to COVID-19) and your medications adjusted as needed.

What will I need to do if I want to take part?

You will have plenty of time to consider your participation after reading this. If you decide you want to take part, the first step is to contact the research team who will arrange your first study visit.

Contact: Dr Jamie Kitt or Mrs Yvonne Kenworthy Tel: 01865 572833 or study mobile 07713 782185

E-mail: Jamie.kitt@cardiov.ox.ac.uk or Yvonne.Kenworthy@cardiov.ox.ac.uk

You can contact us by calling/SMS/e-mail to arrange a study visit, or we will contact you after giving you at least one hour to consider this document. If you are willing to participate in the study, then we will take your written consent.

Chief Investigator:	Prof Paul Leeson	Version/date:	6.0 23.03.2021
Study Short Title:	POP-HT STUDY	Ethics Ref:<br>IRAS ID:	19/LO/1901<br>273353
Document:	Participant Information Sheet		

For peer review only

What should I consider?

You will not be able to take part in this study if the chief investigator deems your taking part would be unsafe and will already have been screened against strict exclusion criteria. Should you have any concerns however, please discuss this with the study team. Due to the fact that the nature of this research study involves home visits, all members of the research team have undertaken the relevant and stipulated safe-guarding training.

Are there any possible disadvantages or risks from taking part?

Your decision on whether you will participate or not **will not affect in any way your clinical care** now or in future. All of the study procedures/assessments are safe but as with any medical procedure, there are some minor risks. More details on the risks associated with certain procedures e.g. cardiac MRI and blood taking are detailed in the ***Supplementary Information Sheet provided with this main PIS***. At all times, an experienced study investigator will be with you and will address any issues that may arise. As per routine clinical advice women in both arms of the trial should continue to monitor their babies for drowsiness, lethargy, pallor, cold peripheries or poor feeding when discharged home.

What are the possible benefits of taking part?

This study is designed to test whether self-management can improve blood pressure control in the period immediately after birth. As part of the study all participants receive 4 additional visits above and beyond usual NHS care and so will be more closely monitored than you otherwise would be. This may result in earlier access to treatment should any abnormal BP readings be detected.

Will my taking part in this study be kept confidential?

Yes. The data collected from the study will be de-identified so that you will be known only by a unique study specific ID. You would not be identifiable from this. Responsible members of the University of Oxford, Oxford University Hospitals NHS Trust and Regulatory Authorities may be given access to data for monitoring and/or audit of the study to ensure that the research is complying with applicable regulations. As part of our commitment to maximise participant involvement in research, participants can give consent (optional) for their contact details to be retained (see section below). As mentioned above, details will be shared with NHS digital to allow longer-term follow up of your blood pressure up to 10 years. NHS digital is regulated by the same strict criteria as this study. In the event that you lose capacity to consent whilst taking part in the study you will be withdrawn from the study and no further data will be collected nor any further assessments/procedures undertaken. Any data that has already been collected will be retained.

What will happen to the samples I give?

Blood samples collected will be analysed for this study, but your samples may also be used for other studies with appropriate ethical approval in the future. Samples collected will be de-identified and stored in secured facilities within the University of Oxford. If you withdraw from the study for any reason, we will retain any

For peer review only - http://bmjopen.bmj.com/site/about/guidelines.xhtml

blood samples and data collected up to that point for use in research as detailed in this participant information sheet. If you agree to your samples being used in future research, your consent form will be held until the samples have been depleted or destroyed.

Will my General Practitioner/family doctor (GP) be informed of my participation?

Your GP will be notified of your study participation and will be provided with a letter or study information sheet. There may also be instances where GPs will be contacted to follow up incidental findings that may be of clinical significance or if you withdraw/are withdrawn from the study.

What will happen to my data?

Data protection regulation requires that we state the legal basis for processing information about you. In the case of research, this is 'a task in the public interest.' The University of Oxford is the data controller and is responsible for looking after your information and using it properly. We will be using information from you, your medical notes and NHS Digital (and other central NHS bodies) in order to undertake this study and will use the minimum amount of personally-identifiable information possible. We will store any research documents with personal/traceable data, such as consent forms and your retinal images, securely at the University of Oxford for 10 years after the end of the study as part of the research record. If you have consented to your samples being retained for future research, a copy of your consent is retained for the duration of sample storage. We keep any other identifiable information about you for up to 12 months after the study is finished. All documents containing personal information such as your informed consent form will be stored securely and only accessible by study staff and authorised personnel only. The Oxford University Hospitals NHS Foundation Trust will use your name, NHS number, date of birth and contact details (address and telephone number) to contact you about the research study, to make sure that the relevant information about the study is recorded for your care, and to oversee the quality of the study. They will keep identifiable information about you from this study for up to 12 months after the study has finished. As part of your participation in the study, in addition to the information you provide about your health, the research team in Oxford will ask for information about your health from NHS Digital (including, but not limited to, NHS Digital). We will send your name, date of birth, NHS number and postcode to NHS Digital (or other central NHS bodies) who can link this information to your centrally held records. Data protection regulation provides you with control over your personal data and how it is used. When you agree to your information being used in research, however, some of those rights may be limited in order for the research to be reliable and accurate. Further information about your rights with respect to your personal data is available at http://www.admin.ox.ac.uk/councilsec/compliance/gdpr/individualrights. You can find out more about how we use your information by contacting jamie.kitt@cardiov.ox.ac.uk.

Chief Investigator:	Prof Paul Leeson	Version/date:	6.0 23.03.2021
Study Short Title:	POP-HT STUDY	Ethics Ref:	19/LO/1901
		IRAS ID:	273353
Document:	Participant Information Sheet		

For peer review only

Will I be reimbursed for taking part?

If you visit us at CCRF we will reimburse travel and parking expenses to and from the CCRF at the John Radcliffe Hospital site, if you provide receipts and/or mileage details. **You will also receive £30 thank-you** for your participation in the study after the final study visit. The app notifications and usage are free when in WIFI zone but if using 3G/4G they may be charged depending on your network-provider. If this is the case, then any cost incurred will be reimbursed to you on production of the relevant bill.

What will happen if I don't want to carry on with the study?

You can withdraw from the study at any time without giving a reason. If you decide you no longer wish to take part in our study, you can phone, write to, or e-mail Dr Jamie Kitt using the contact details listed in the header on page one. Should you wish to withdraw, please let us know if we can keep the information we have collected about you so far as we may be unable to destroy the data if it has already been de-identified, as outlined in the confidentiality section. Data and samples already collected would not be used in the final study analysis except where analysis of their data or samples has already been integrated into interim results.

What will happen to the results of the study?

Summarised results will be published in scientific journal/s and also summarised on our website, after completion of the study, for you to read: https://www.rdm.ox.ac.uk/about/our-clinical-facilities-and-mrc-units/cardiovascular-clinical-research-facility/ongoing-clinical-studies.

What if we find something unexpected?

If your blood pressure readings are very high (**above 160/110mmHg**), we will inform you of this finding immediately with instructions on what to do next and we will also call you to inform you to seek medical assistance. In the unlikely event that we detect any structural abnormalities during the scan of your heart (echocardiogram) or MRI of your heart or brain, then with your permission we will refer you for assessment by contacting your GP and/or a specialist hypertension clinic, who can arrange instigate any necessary investigations and treatment.

What if there are any problems?

The University of Oxford, as Sponsor, has appropriate insurance in place in the unlikely event that you suffer any harm as a direct consequence of your participation in this study. NHS indemnity operates in respect of the clinical treatment which is provided. If you wish to complain about any aspect of the way in which you have been approached/treated during this study, you should contact, the Chief Investigator, Prof Paul

For peer review only - http://bmjopen.bmj.com/site/about/guidelines.xhtml

Leeson on +44 (0)1865 572846 or e-mail: paul.leeson@cardiov.ox.ac.uk. You may contact the University of Oxford Clinical Trials and Research Governance (CTRG) office on 01865 616480, or the head of CTRG, email ctrg@admin.ox.ac.uk.

How have patients and the public been involved in this study?

Members of the public, who have been through similar pregnancy related medical problems, have been involved in the design of this study, testing and refining of the intervention arm being trialled and several of the documents including the poster, logo and this patient information leaflet.

Who is organising and funding the research?

This study has been designed and organised by investigators of the University of Oxford, Division of Cardiovascular Medicine and Nuffield Department of Primary Care Health Sciences (namely Prof Paul Leeson, Dr Jamie Kitt, Professor Richard McManus, Dr Lucy Mackillop and Dr Adam Lewandowski). If you wish to know more about any aspect of the study, please contact Jamie Kitt on 01865 (5)72833 or jamie.kitt@cardiov.ox.ac.uk. Dr Jamie Kitt is conducting this research as part of his doctoral studies and results from this study may be used in anonymous form to support this. The research is being financed by the British Heart Foundation. The sponsor of the study is the University of Oxford.

Who has reviewed the study?

All research in the NHS is looked at by an independent group of people called a Research Ethics Committee, to protect your safety, rights, wellbeing and dignity. This study has been reviewed by London Surrey REC [19/LO/1901].

Participation in future research:

If you consent to be considered for future studies, a copy of this consent and your contact details will be kept securely and independently of the study records in a separate, secure database in order that we can contact you if further research in this area is being under-taken. You are under no obligation to consent to being contacted again and we understand you may want to participate only in this study in which case your details will not be kept as explained in the data confidentiality section above. You have the right to ask for your personal information to be removed from this database at any time.

Further information and contact details:

Lead Study Investigator: Dr Jamie Kitt; Tel: 01865 572833 Email: jamie.kitt@cardiov.ox.ac.uk

Chief Investigator: Prof Paul Leeson; Tel: 01865 572846 Email: paul.leeson@cardiov.ox.ac.uk

Amendments during COVID-19 pandemic

Chief Investigator:	Prof Paul Leeson	Version/date:	6.0 23.03.2021
Study Short Title:	POP-HT STUDY	Ethics Ref:	19/LO/1901
		IRAS ID:	273353
Document:	Participant Information Sheet		

For peer review only

During the COVID-19 pandemic, this PIS and the flyer will be provided electronically via a tablet, which the clinical team will give you, although there will be less time to consider this than normal due to expedited discharge processes during the pandemic. Consent forms will be placed in wipe down wallets, which will be handed to you for signing and then photocopied whilst wearing gloves. The copy will be placed back into a sterile wallet for you and the original will be placed in a second wallet. Both will be wiped down with CLINELL® wipes and our copy will be kept securely in quarantine before moving them to CCRF. The baseline visit has been adjusted to reduce the amount of direct patient contact to the blood pressure measurements, the ultra-sound (echo), and the Vicorder test is now optional. There will not be a second female chaperone from the research team (this will not affect normal hospital chaperone rules). Questionnaires are e-mailed out and can be completed on a tablet/computer at a later date. All direct contact will be done in PPE where necessary in line with hospital policy. The control arm will also be provided with a blood pressure monitor, when NHS monitors are not available prior to discharge to allow home monitoring by NHS GPs/mid-wives in line with updated RCOG guidance. The study will be performing remote follow up visits for week 1 and week 6 (visits 2 and 3). The final (4th visit) is being extended from 6-9 months, to 6-12 months by which time 'normality' will hopefully have been resumed to allow the final visit to take place at the John Radcliffe Hospital. In extenuating circumstances, such as COVID-19 national lockdowns some part of the V4 will also be done remotely, the 24hr BP monitor fitting, the accelerometer/wrist-watch fitting, the review of the medical history, demographics, questionnaires, and manual blood pressure measurements may also be conducted remotely via video or phone call. The remaining procedures of the final visit will be done when it is safe to do within the 12 month time-window.

For peer review only

Tel: 01865 572833
Email: jamie.kitt@cardiov.ox.ac.uk
Fax: +44(0)1865 572840

Study Code:			*Participant identification number:*		
P	O	—			

Physician Optimised Post-partum Hypertensive Treatment (POP-HT) Study

CONSENT FORM

Name of Researcher:

Participant Name:

If you agree, please initial each box

1. I confirm that I have read the information sheet dated V3.0 06/05/20 for this study. I have had the opportunity to consider the information, ask questions and have had these answered satisfactorily.	
2. I understand that my participation is voluntary and that I am free to withdraw at any time without giving any reason, without my medical care or legal rights being affected.	
3. I understand that relevant sections of my medical records and data collected during the study may be looked at by individuals from University of Oxford, hosting NHS organisations and regulatory authorities, where it is relevant to my taking part in this research. I give permission for these individuals to have access to my records.	
4. MRI/Echocardiography/other research tests: I understand that these are research scans/tests that are not useful for medical diagnosis, and that scan/test results are not routinely looked at by a doctor. If a concern is raised about a possible abnormality on my scan/research test, I will only be informed if a doctor thinks it is medically important such that the finding has clear implications for my current or future health.	
5. I agree for my GP to be informed of any results of medical tests performed as part of the research that may be important for my health care.	
6. If I withdraw/I am withdrawn from the study we will contact your GP to inform them, in order to ensure that any on-going care you require is reinstated.	
7. I agree that the information held and maintained by NHS Digital/ Office for National Statistics (ONS) may be used to provide information about my health	

Chief Investigator:	Prof Paul Leeson	Version/date:	6.0 23.03.2021
Study Short Title:	POP-HT STUDY	Ethics Ref:	19/LO/1901
		IRAS ID:	273353
Document:	Participant Information Sheet		

For peer review only

status. I understand that my name, NHS number, date of birth and postcode may be shared securely to obtain such information and allow contact for blood pressure, and other relevant measurements over the next 10 years.		
8. I agree to donate blood samples. I consider these samples a gift to the University of Oxford and I understand I will not gain any direct personal or financial benefit from them.		
9. I understand that retinal images will be taken as part of the study and will be stored in a de-identified format on the high compliance (secure) server of the University of Oxford for up to 10 years		
10. I agree to take part in this study		
Additional:	Yes	No
11. I agree for my anonymised samples to be used in future research, here or abroad, which has ethics approval. I understand this research may involve commercial organisations.		
12. I agree to be contacted about ethically approved research studies for which I may be suitable. I understand that agreeing to be contacted does not oblige me to participate in any further studies.		
13. I have been approached about the optional additional measure: Vicorder ® assessment of the aortic stiffness and aortic blood pressure		
14. I have been approached about the optional additional measure: Laser Speckle Tracking of the skin on my fore-arm and agree to partake in this additional measure (being done in 48 participants out of 200)		

Name of Participant *Date* *Signature*

Name of Person taking Consent *Date* *Signature*

**** For researchers please tick (✓) to document:***

The original signed form will be placed in the medical notes and a further copy will be retained at the trial site and one given to the participant ***(   )***

Chief Investigator:	Prof Paul Leeson	Version/date:	6.0 23.03.2021
		Ethics Ref:	19/LO/1901
Study Short Title:	POP-HT STUDY	IRAS ID:	273353
Document:	Participant Information Sheet		

For peer review only

For peer review only

Chief Investigator:	Prof Paul Leeson	Version/date:	6.0 23.03.2021
Study Short Title:	POP-HT STUDY	Ethics Ref:	19/LO/1901
		IRAS ID:	273353
Document:	Participant Information Sheet		

SPIRIT

STANDARD PROTOCOL ITEMS: RECOMMENDATIONS FOR INTERVENTIONAL TRIALS

SPIRIT 2013 Checklist: Recommended items to address in a clinical trial protocol and related documents*

Section/item	Item No	Description
**Administrative information**		
Title	1	A Randomised control trial of post-partum blood pressure self-management following hypertensive pregnancy: Physician Optimised Post-partum Hypertension Treatment (POP-HT) trial protocol paper
Trial registration	2a	NCT04273854 at Clinicaltrials.gov
	2b	All items from the World Health Organization Trial Registration Data Set
Protocol version	3	V6.0 08/03/2021
Funding	4	BHF Grant number FS/19/7/34148
Roles and responsibilities	5a	See title page and section on authors contributions
	5b	See title page
	5c	See section titled Publication Policy: Role of study sponsor and funders, if any, in study design; collection, management, analysis, and interpretation of data; writing of the report; and the decision to submit the report for publication, including whether they will have ultimate authority over any of these activities
	5d	See section on Trial Monitoring: Composition, roles, and responsibilities of the coordinating centre, steering committee, endpoint adjudication committee, data management team, and other individuals or groups overseeing the trial, if applicable (see Item 21a for data monitoring committee)
**Introduction**		
Background and rationale	6a	See Introduction section
	6b	
Objectives	7	See Hypotheses section

For peer review only - http://bmjopen.bmj.com/site/about/guidelines.xhtml

Trial design	8	See section on Study design within METHODS

Methods: Participants, interventions, and outcomes

Study setting	9	See Section titled 'Study design'
Eligibility criteria	10	See Section titled 'Eligibility and recruitment
Interventions	11	SEE METHODS AND APPENDICES A-E
Outcomes	12	See Section titled 'Study aims and objectives'
Participant timeline	13	See FIGURE 1 within METHODS and APPENDICES A-C
Sample size	14	See Section titled 'DATA ANALYSIS'
Recruitment	15	See METHODS

Methods: Assignment of interventions (for controlled trials) :

Sequence generation	16a	See Section titled 'Randomisation'. More detail in full study protocol and SAP
Allocation concealment mechanism	16b	See Section titled 'Randomisation'. More detail in full study protocol and SAP
Implementation	16c	See Section titled 'Randomisation'. More detail in full study protocol and SAP

Methods: Data collection, management, and analysis: See Sections titled 'Assessments during study visits', 'Data analysis' and 'SAP'

Data collection methods	18a	Plans for assessment and collection of outcome, baseline, and other trial data. DATA collection is all electronic using CASTOR® EDC.
	18b	See section on PPI and SAP and further detail in the full study protocols

Data management	19	See section titled 'Data Management'. Full plans for data entry, coding, security, and storage are detailed in the full study protocol and summaries in the participant information sheet (PIS)
Statistical methods	20a	See SAP summary within the protocol paper. Full SAP to be produced
	20b	Full SAP to be produced imminently
	20c	Full SAP to be produced imminently
**Methods: Monitoring: See sections titled 'TRIAL OVERSIGHT' and 'SAFETY REPORTING'**		
Data monitoring	21a	See 'Trial Committees'
	21b	Description of any interim analyses and stopping guidelines, including who will have access to these interim results and make the final decision to terminate the trial
Harms	22	See ' Trial oversight and Safety Reporting sections'
Auditing	23	See ' Monitoring' section
**Ethics and dissemination: See Section titled 'Ethics and Dissemination' and Supplementary Material Appendices E and F**		
Research ethics approval	24	19/LO/1901 is the RE/HRA approval reference
Protocol amendments	25	See Supplementary materials: appendices E and F
Consent or assent	26a	Detailed in full trial protocol in more detail but summarised in Section 'Eligibility and Recruitment' and 'Ethics and Dissemination'
	26b	See Appendices section below re consent for biological specimens
Confidentiality	27	See section on 'Participant Confidentiality'
Declaration of interests	28	See section titled 'Competing interests'
Access to data	29	See full trial protocol for section on data access, storage and management
Ancillary and post-trial care	30	See section titled Insurance
Dissemination policy	31a	See section titled Publication policy
	31b	See section titled Publication policy
	31c	See section titled Publication policy

For peer review only - http://bmjopen.bmj.com/site/about/guidelines.xhtml

**Appendices:**		**Appendices are referenced in the main body of the text where relevant and are uploaded in the Supplementary file.**
Informed consent materials	32	See 'Appendix G' in the supplementary file
Biological specimens	33	See relevant sections of the consent forms and PIS in Appendix G of the supplementary file. Further details are also contained in the full study protocol

*It is strongly recommended that this checklist be read in conjunction with the SPIRIT 2013 Explanation & Elaboration for important clarification on the items. Amendments to the protocol should be tracked and dated. The SPIRIT checklist is copyrighted by the SPIRIT Group under the Creative Commons "Attribution-NonCommercial-NoDerivs 3.0 Unported" license.

For peer review only
